# Heterodimeric GW7604 Derivatives: Modification of the Pharmacological Profile by Additional Interactions at the Coactivator Binding Site

**DOI:** 10.1021/acs.jmedchem.0c02230

**Published:** 2021-04-27

**Authors:** Alexandra K. Knox, Christina Kalchschmid, Daniela Schuster, Francesca Gaggia, Ronald Gust

**Affiliations:** † Department of Pharmaceutical Chemistry, Institute of Pharmacy, CMBI − Center for Molecular Biosciences Innsbruck, University of Innsbruck, CCB − Center for Chemistry and Biomedicine, 6020 Innsbruck, Austria; ‡ Department of Pharmaceutical and Medicinal Chemistry, Institute of Pharmacy, Paracelsus Medical University, 5020 Salzburg, Austria

## Abstract

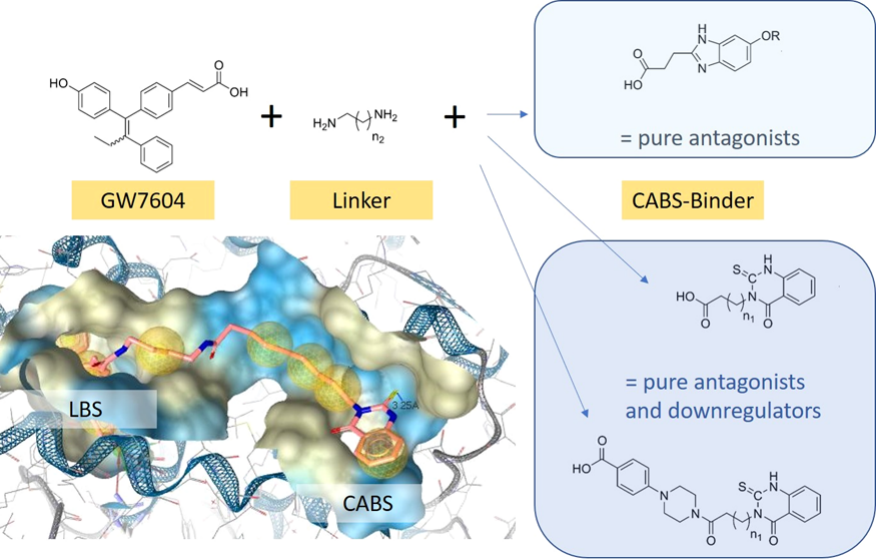

(*E*/*Z*)-3-(4-((*E*)-1-(4-Hydroxyphenyl)-2-phenylbut-1-enyl)phenyl)acrylic acid (GW7604) as a derivative of (*Z*)-4-hydroxytamoxifen (4-OHT) was linked by diaminoalkane spacers to molecules that are known binders to the coactivator binding site (benzimidazole or thioxo-quinazolinone scaffolds). With this modification, an optimization of the pharmacological profile was achieved. The most active thioxo-quinazolinone derivative **16** showed extraordinarily high affinity to the estrogen receptor (ER) β (RBA = 110%), inhibited effectively the coactivator recruitment (IC_50_ = 20.88 nM (ERα) and 28.34 nM (ERβ)), acted as a pure estradiol (E2) antagonist in a transactivation assay (IC_50_ = 18.5 nM (ERα) and 7.5 nM (ERβ)), and downregulated the ERα content in MCF-7 cells with an efficacy of 60% at 1 μM. The cytotoxicity was restricted to hormone-dependent MCF-7 (IC_50_ = 4.2 nM) and tamoxifen-resistant MCF-7TamR cells (IC_50_ = 476.6 nM). The compounds bearing a thioxo-quinazolinone moiety can therefore be assigned as pure E2-antagonistic selective ER degraders/downregulators. By contrast, the benzimidazole derivatives acted solely as pure antagonists without degradation of the ER.

## 1 Introduction

Breast cancer is still the most common cancer in women worldwide, affecting one in eight women in high-income countries, and the incidence is further increasing.^1^ The majority of all types of mammary carcinomas (MCs) are, at least initially, hormone-dependent.^2,3^ Endocrine therapy, including aromatase inhibitors or selective estrogen receptor modulators (SERMs)/selective estrogen receptor downregulators (SERDs), consequently represents an indispensable treatment opportunity. Unfortunately, acquired endocrine resistance is an inevitable issue, which manifests after prolonged therapy.^4,5^

With regard to genomic alterations, endocrine-resistant breast tumors are divided into four groups: (i) tumors bearing estrogen receptor alpha (ERα) gene alterations (ESR1), (ii) tumors harboring lesions in the mitogen-activated protein kinase pathway, (iii) tumors with mutations in the transcriptional factors, and (iv) tumors with undiscovered resistance mechanisms.^6,7^ ESR1 alterations represent, with approximately 20%, the largest category.^6,8^ These ESR1 point mutations, for instance, are particularly frequently located in the ligand binding domain (LBD) and thus require the search for new antiestrogenic drugs to overcome this kind of resistance.^9^

**Figure 1:**
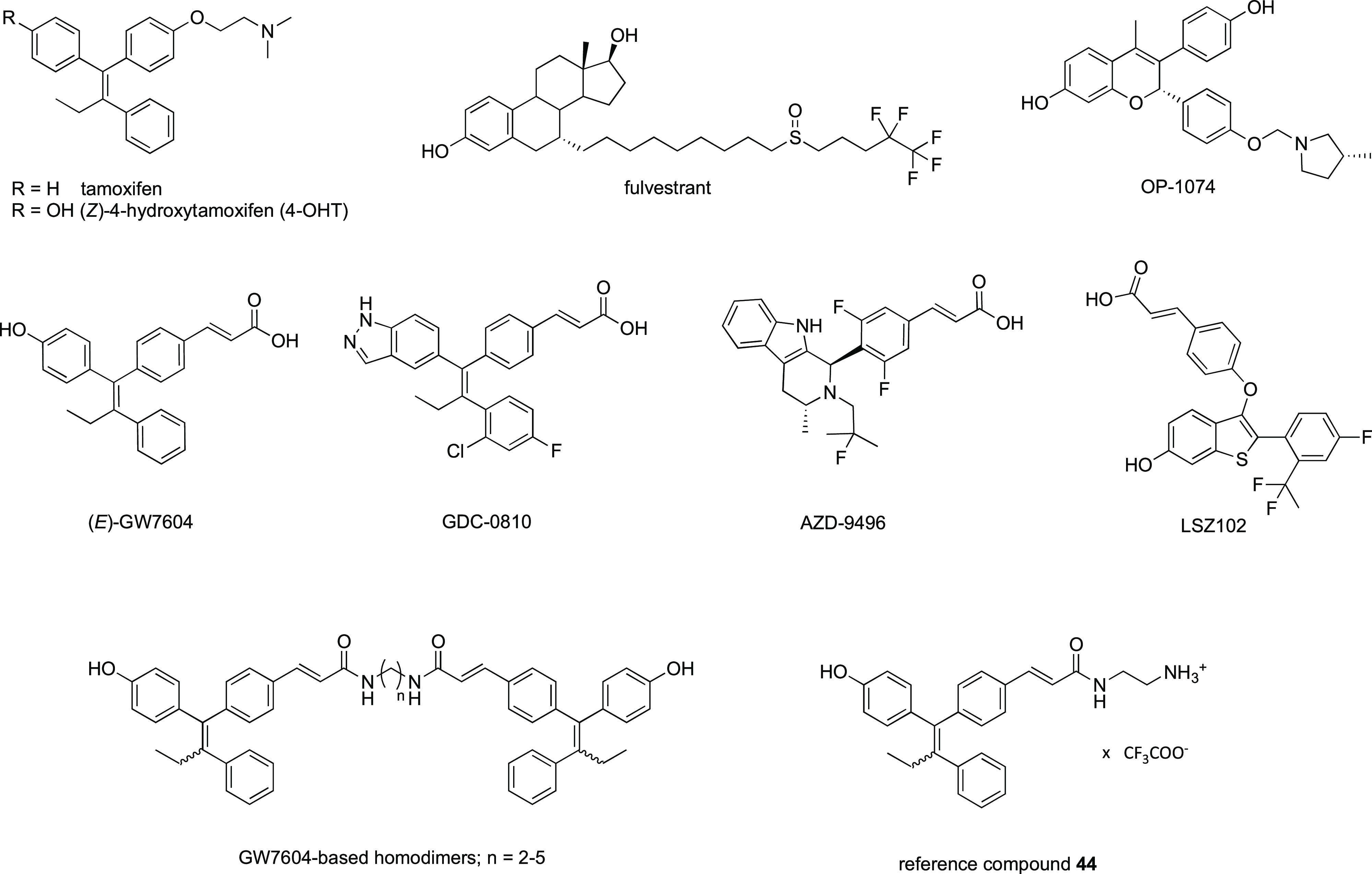
SERMs: tamoxifen and 4-OHT. SERDs: fulvestrant and OP-1074. SERM-SERDs: (*E*)-GW7604, GDC-0810, AZD-9496, and LSZ102. GW7604-based homodimers and reference compound **44**.

One possibility to impede estrogen-mediated pathways, in general, is to induce specific conformations of the estrogen receptor (ER) upon drug binding, which prevents coactivator recruitment and ultimately gene transcription in hormone-dependent MC cells. The SERM tamoxifen (Figure 1), as the first targeted anti-breast-cancer therapeutic agent,^10^ acts *via* this mode of action. Upon attachment of an agonist at the ligand binding site (LBS), Helix 12 (H12) is oriented over the LBD. With parts from helices H3, H4, H5, and H12, and the turn between helices H3 and H4, a hydrophobic groove (activation function 2 (AF2)) that accommodates an LXXLL motif is formed. The AF2 is essential for coactivator binding. In the case of (*Z*)-4-hydroxytamoxifen (4-OHT), which is the active metabolite of tamoxifen, H12 is repositioned, AF2 is not formed, and interactions with coactivator peptides are blocked in hormone-dependent tumor cells, preventing their growth.^11,12^ Nevertheless, in some other target tissues (endometrium, bones), activation is possible due to an insufficient shielding of the charge at Asp351 (at ERα) by the basic side chain. It is noteworthy that in one-third of patients acquired resistance against tamoxifen occurs within 15 years.^13^ In this case, the use of aromatase inhibitors (e.g., anastrozol) or SERDs (fulvestrant (Figure 1)) is indicated.^14^

Fulvestrant disrupts ER dimerization and nuclear localization. Furthermore, it accelerates receptor degradation. This mode of action is based on unusual conformational change of the ER upon drug binding. It sterically prevents H12 from forming any type of interaction with residues of the ER. Consequently, H12 is destabilized, hydrophobic surfaces at the ER are exposed, and the receptor is submitted to downregulation by the ubiquitin-proteasome pathway.^12,15^ Consequently, the ER-mediated transcriptional activity is completely blocked, leading to an effective growth reduction of hormone-dependent tumors. OP-1074 (Figure 1), declared as a pure antiestrogen and SERD, displays the same mechanism as fulvestrant.^16^ To derive an alternative after failure of tamoxifen treatment, 4-OHT was structurally modified in a way where the basic side chain was exchanged by a carboxylate bearing moiety. (*E*/*Z*)-3-(4-((*E*)-1-(4-Hydroxyphenyl)-2-phenylbut-1-enyl)phenyl)acrylic acid (GW7604) (Figure 1), the cinnamic acid analogue of 4-OHT, showed diminished hormonal effects, e.g., induction of ER expression and cell-growth-stimulating effects at low concentrations.^17,18^ This is ascribed to the repulsion of the carboxylate and the amino acid Asp351 (ERα). The conformational change disrupts the surface charge around this amino acid required for coactivator binding in the 4-OHT/ER complex.^19^ Unlike 4-OHT, the binding of GW7604 even causes downregulation of the receptor. Since H12 is not totally destabilized as found for the fulvestrant-bound receptor and less hydrophobic sites are exposed, degradation occurs, but to a low extent.^18^ Further modifications mainly include the change in the LBS-binding core. Examples are GDC-0810,^20^ AZD-9496,^21^ and LSZ102^22^ (Figure 1), referred to as SERM-SERDs^16^ or as SERM/SERD hybrids.^23^

In our group, we used another approach and developed compounds in such a way that besides the LBS, the coactivator binding site (CABS) is targeted simultaneously. We evaluated the consequences on the receptor binding affinity and the intracellular responses.

In a first study, homodimers of GW7604 and of the related cyclofenilacrylic acid were designed, because an X-ray crystal structure revealed a hydrophobic groove at the CABS suitable to bind 1,1-diaryl- or 1,1,2-triarylalkenes (see chapter 2.1). Alkyl spacers of different lengths between the molecules should guarantee sufficient flexibility to reach two different pockets within this exposed surface, which emerge as potential binding areas.^24^ As proposed, it was possible to increase the binding affinity and to inhibit ER transactivation.

**Chart 1:**
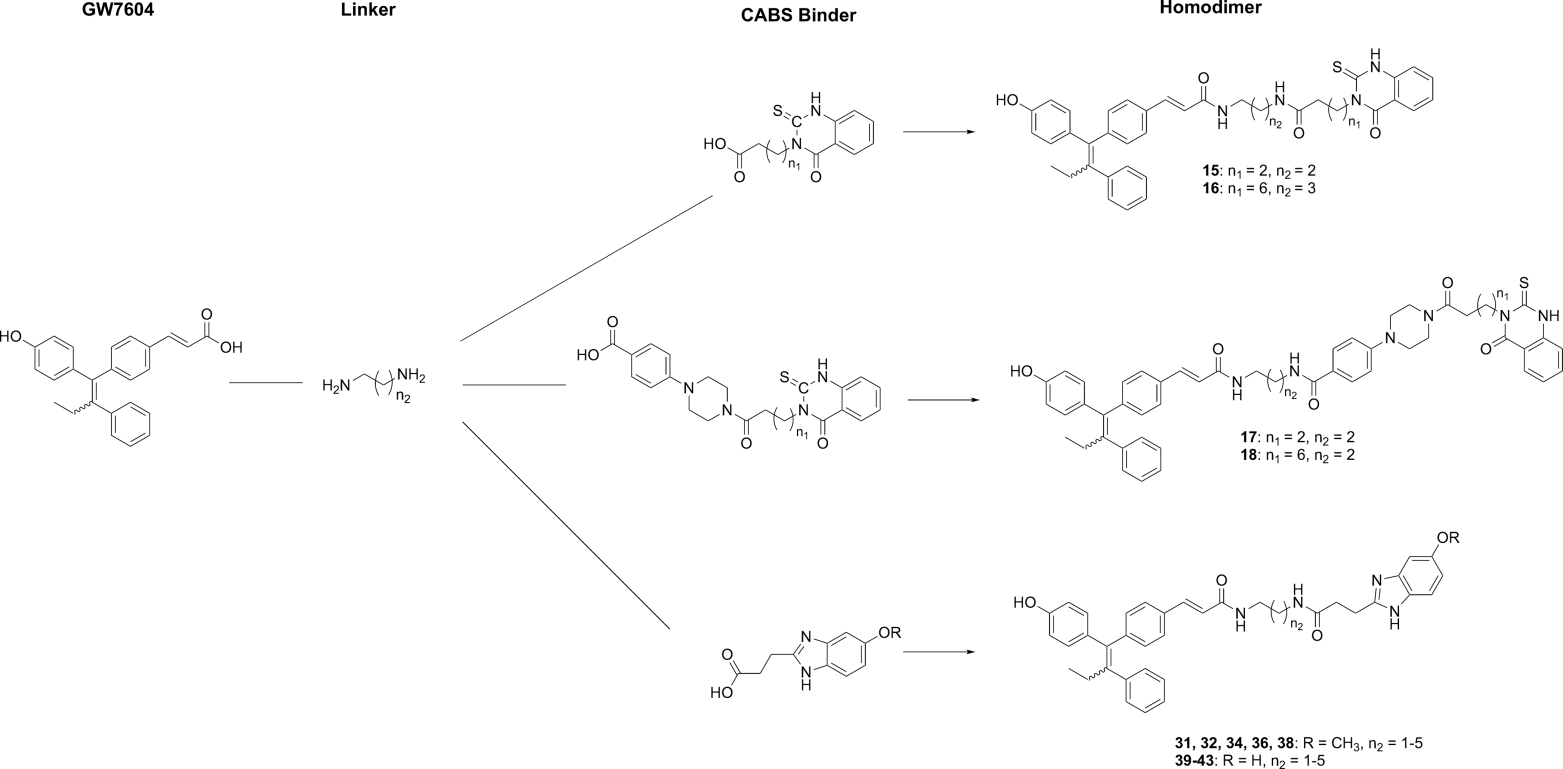
Design of Heterodimeric ER Ligands

In continuation of this structure–activity relationship (SAR) study, we tried to optimize the CABS-binding properties. 1,1-Diarylalkene derivatives can bind at the ER surface, but steric repulsion might render accessibility to the proposed binding pockets. Therefore, GW7604 was linked to molecules, which were already described as CABS binders. We selected thioxo-quinazolinone derivatives (e.g., 4-(4-(4-(4-oxo-2-thioxo-1,4-dihydroquinazolin-3(2*H*)-yl)butanoyl)piperazin-1-yl)benzoic acid or 4-(4-oxo-2-thioxo-1,4-dihydroquinazolin-3(2*H*)-yl)butanoic acid^25^) and the 3-(5-methoxy-1*H*-benzo[*d*]imidazol-2-yl)propanoic acid (Chart 1) to be connected *via* the diaminoalkane spacer to GW7604. The 5-hydroxybenzimidazole scaffold was also investigated regarding their H-bond formation within the pockets at the ER surface.

The binding affinities of the compounds at the isolated LBD, their cellular responses such as inhibition of gene activation, ER downregulation, and antiproliferative effects in hormone-dependent/-independent as well as in tamoxifen-resistant tumor cells (resistance group (ii) as mentioned above) were evaluated. Furthermore, quantitative cellular uptake studies were performed to rationalize the influence of compound accumulation on the cellular activity.

## 2 Results and Discussion

### 2.1 Docking Studies

Previously, we described a theoretical model to evaluate the binding of bivalent molecules at the ER. It is based on the crystal structure of the ERβ-LBD (PDB entry 2FSZ)^26^ cocrystallized with two 4-OHT molecules.^24^ The first one is attached at the LBS and the second one at the CABS. Both can formally be connected by an alkyl spacer, enabling a view on possible binding modes of homodimeric compounds.

GW7604-based bivalent derivatives (Figure 1) were already synthesized and tested for ER interactions. It is postulated that the GW7604 moiety binds in the LBS of ERβ forming H-bonds to Arg346, Glu305, and one water molecule in a classic manner,^27,28^ while the terminal drug molecule interacts at the hydrophobic surface. These interactions were considered as a prerequisite for being a valid docking pose.

As a further development, GW7604 is linked to scaffolds of known CABS binders (formation of heterodimeric compounds). A suitable one represents the 4-(4-oxo-2-thioxo-1,4-dihydroquinazolin-3(2*H*)-yl)butanic acid. The use of various diaminoalkyl spacers (*n*_2_ = 2, 3, Chart 1) allows binding to side pockets along the identified hydrophobic channel at the CABS. In addition, the influence of alkanoic acid (*n*_1_ = 2, 6, Chart 1) at the thioxo-quinazolinone core was studied.

**Figure 2:**
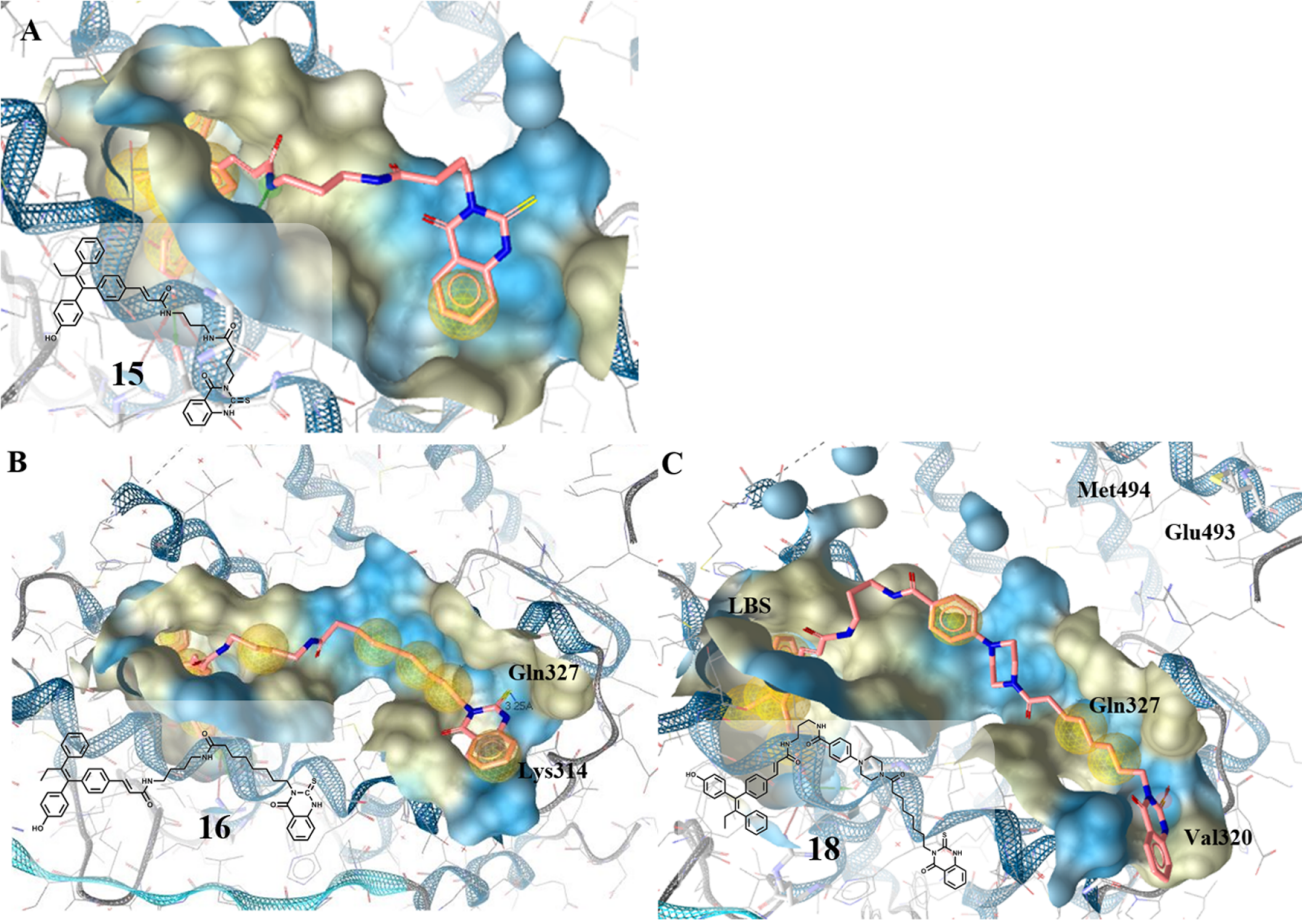
Ligand binding pocket of the ERβ-LBD (PDB entry 2FSZ)^26^ with (A) **15**, (B) **16**, and (C) **18** depicted in rose. Hydrophobic protein–ligand interactions are shown as yellow spheres and H-bonds by red and green arrows.

The best docking results revealed the combination of an *N*-butanoic acid chain (*n*_1_ = 2) with a C3 spacer (*n*_2_ = 2: compound **15**, Chart 1). In this case, the thioxo-quinazolinone core reached the hydrophobic groove 9 Å away from the nitrogen atom of the GW7604 amide (Figure 2A). The second binding region, which is about 18–20 Å apart, was targeted employing an octanoic acid residue (*n*_1_ = 6) and a 1,4-diaminobutane chain (*n*_2_ = 3: compound **16**, Chart 1). In this pocket, cation−π interaction with Lys314 in addition to multiple hydrophobic contacts is possible. The distance to Gln327 is 3.25 Å, and there might be a possibility to render further interactions (Figure 2B).

In a second attempt, we used 4-(4-(4-(4-oxo-2-thioxo-1,4-dihydroquinazolin-3(2*H*)-yl)butanoyl)piperazin-1-yl)aryl derivatives initially discovered as CABS binders by Sun et al.^25^ Introduction of a 4-COOH group at the aryl ring makes the connection to GW7604 *via* a diaminoalkane spacer (1,3-diaminopropane (*n*_2_ = 2)) possible. The best results provided **17** (*n*_1_ = 2), allowing an attachment comparable to that postulated by Sun et al.^25^ To assess the influence of higher flexibility and to reach areas at the ER comparable to **16**, the alkanoic acid chain at the thioxo-quinazolinone was elongated (*n*_1_: 2 → 6).

Compound **18** (*n*_1_ = 6) adapted indeed a similar position at the ER (compare Figure 2B,C). The position of the thioxo-quinazolinones differed from that described by Sun et al.,^25^ whereby the piperazinylbenzoate moiety was pulled away from the position close to charge clamp residues Glu493 and Met494 more toward the LBS, whereas the thioxo-quinazolinone ring remained in the area near Lys314 and Gln327. This orientation enabled an H-bond between the C=S group and Gln327 and diminished the interaction between the heteroaromatic amine group and Val320.

**Figure 3:**
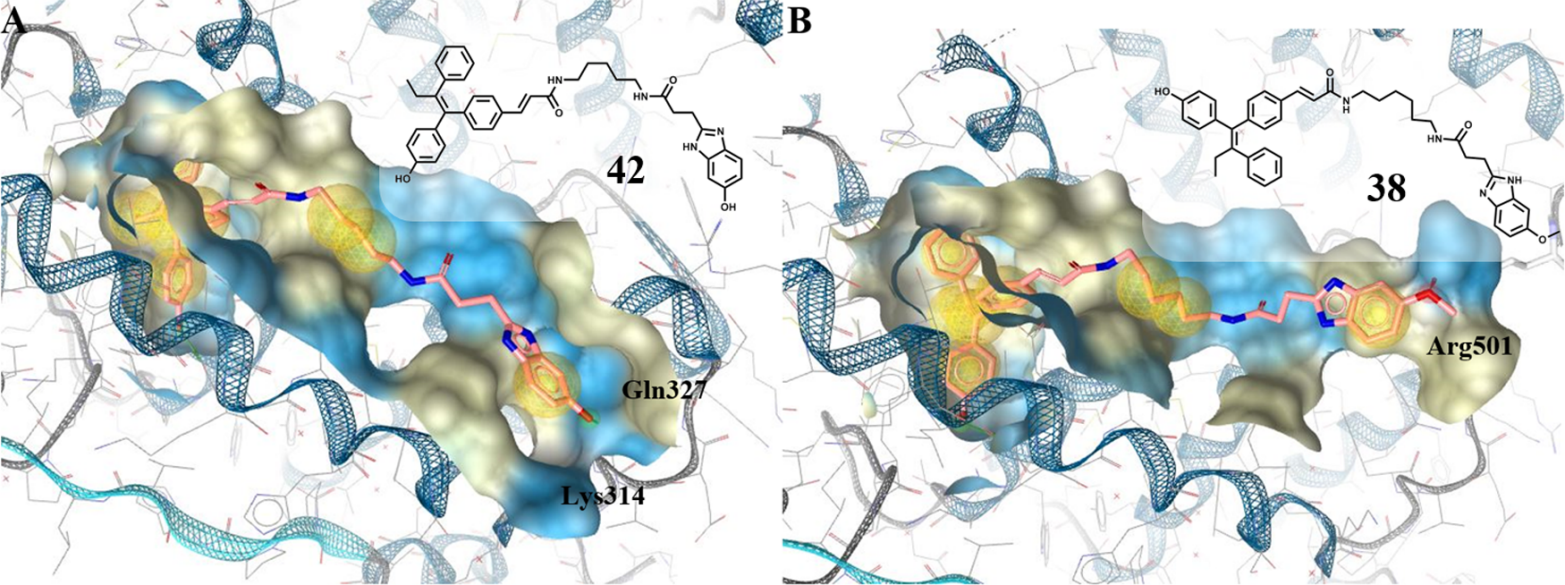
Ligand binding pocket of the ERβ-LBD (PDB entry 2FSZ)^26^ with (A) **42** and (B) **38** in rose.

To further reduce the steric demand of the CABS binder, and to occupy the hydrophobic pocket closer to the LBS, the 3-(5-methoxy/hydroxy-1*H*-benzo[*d*]imidazol-2-yl)propanoic acid moiety was introduced. C-alkylated benzimidazole scaffolds have already been recognized as CABS binders at the ER,^29^ but were also used as essential cores for the design of partial peroxisome proliferator-activated receptor γ (PPARγ) agonists.^30,31^ The conjugation to GW7604 was again performed by diamide formation (*n*_2_ = 1–5). Interestingly, the 5-methoxybenzimidazole derivatives **31** and **32** with a short C2 or C3 linker (*n*_1_ = 1, 2) mainly targeted a hydrophobic pocket close to the LBS, while longer chains allowed the attachment as discussed above. Upon ether cleavage, the resulting 5-hydroxybenzimidazole derivatives with an extended alkyl chain (C5: **42**; C6: **43**) caused H-bond formation with Gln327, which is also in close vicinity (4–5 Å) to the charge clamp residue Lys314 (Figure 3A).

This interaction was prevented in 5-methoxybenzimidazoles. But surprisingly, an H-bond with Arg501 located in H12 was possible, causing reorientation in the binding cleft, inducing a similar position of both long-chained heterodimers in the LBD (Figure 3B).

In summary, the theoretical investigations document the accessibility of the proposed binding areas within the CABS using thioxo-quinazolinone and benzimidazole scaffolds. Hydrophobic contacts as well as H-bonding with either Lys314 or Gln327 depend on the spacer length and the used heteroaromatic scaffold. Based on the above-described theoretical investigations, a selection of compounds was synthesized to investigate the predicted interactions and to explore the assignability of those to the ERα.

### 2.2 Chemistry

#### 2.2.1 Synthesis

**Scheme 1:**
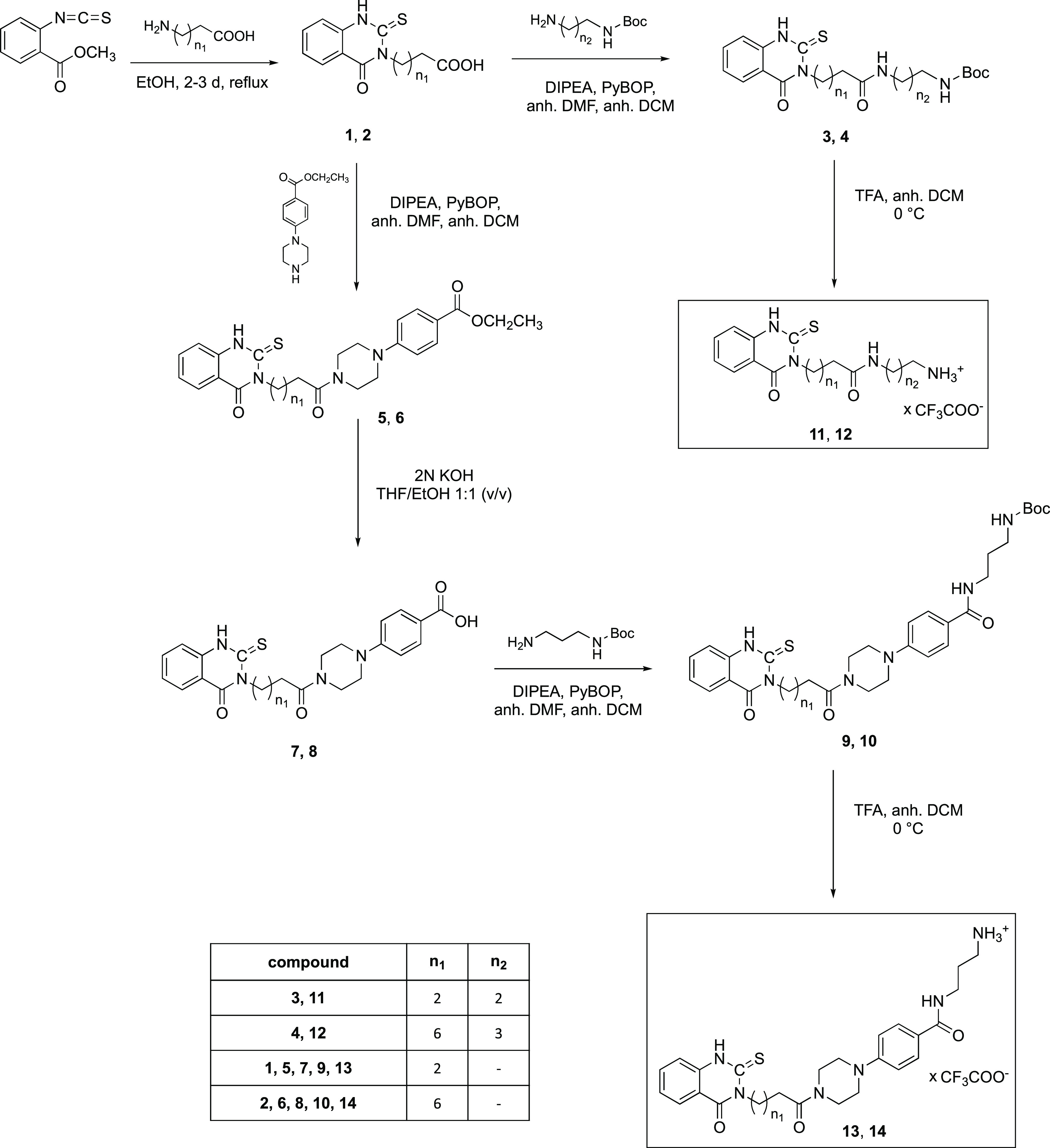
Synthesis Pathway for the Thioxo-quinazolinone Trifluoroacetate Building Blocks **11**–**14**

The thioxo-quinazolinone-based CABS-binding motifs were obtained from building blocks **1** and **2**, which differ in their alkyl chain (*n*_1_ = 2, 6). The carboxylate was derived in cases of **11** and **12** with a simple diaminoalkane chain (*n*_2_ = 2, 3), while in **13** and **14** a piperazinylbenzoate scaffold was used to combine the 1,3-diaminopropane spacer and the COOH group of **1** and **2***via* amide bonding (Scheme 1).

According to the published procedure by Sun et al.,^25^ the thioxo-quinazolinone ring closure to **1** and **2** occurred in ethanol (EtOH) under reflux, however, without the need for additional KOH. In fact, KOH led to a partial decomposition of the scaffold. ^1^H Nuclear magnetic resonance (NMR) spectroscopy and high-resolution mass spectrometry (HR-MS) confirmed the successful ring formation (see the Supporting Information).

The amide syntheses (**3**, **4**, **5**, **6**, **9**, **10**) utilized the coupling reagent benzotriazol-1-yloxytripyrrolidinophosphonium hexafluorophosphate (PyBOP) and the auxiliary base diisopropylamine (DIPEA) in dry dichloromethane (DCM) and dimethylformamide (DMF).^32−35^ The workup under acidic conditions (pH 3–4) guaranteed removal of basic byproducts. Three out of the four piperazinylbenzoate containing compounds (**5**, **6**, and **9**) precipitated from the reaction mixture as a result of their poor solubility in DCM/DMF. Generally, the yields were good to excellent, ranging from 54 to 92%. Compound **10** was separated from unwanted side products by column chromatography.

The carboxylic acids **7** and **8** as educts for the syntheses of **9** and **10** were obtained from esters **5** and **6** by ester cleavage with KOH in tetrahydrofuran (THF) and ethanol 1:1 (v/v). The cleavage of the *tert*-butyloxycarbonyl (Boc) protecting group from **3**, **4**, **9**, and **10** with trifluoroacetic acid (TFA) in dry DCM^34,36^ yielded the trifluoroacetate salts **11**–**14** in quantitative yield.

**Scheme 2:**
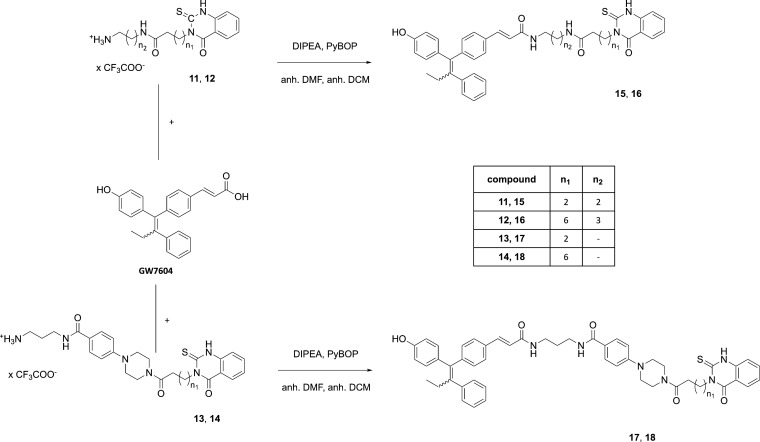
Reaction of Thioxo-quinazolinone Trifluoroacetate Building Blocks with GW7604

Finally, the reaction of GW7604 with amines **11**–**14** using PyBOP/DIPEA gave the heterodimeric products **15**–**18** (Scheme 2). In each case, the purification by column chromatography led to a loss of compound (yields: **15** (29%), **16** (26%), **17** (54%), and **18** (48%)).

**Scheme 3:**
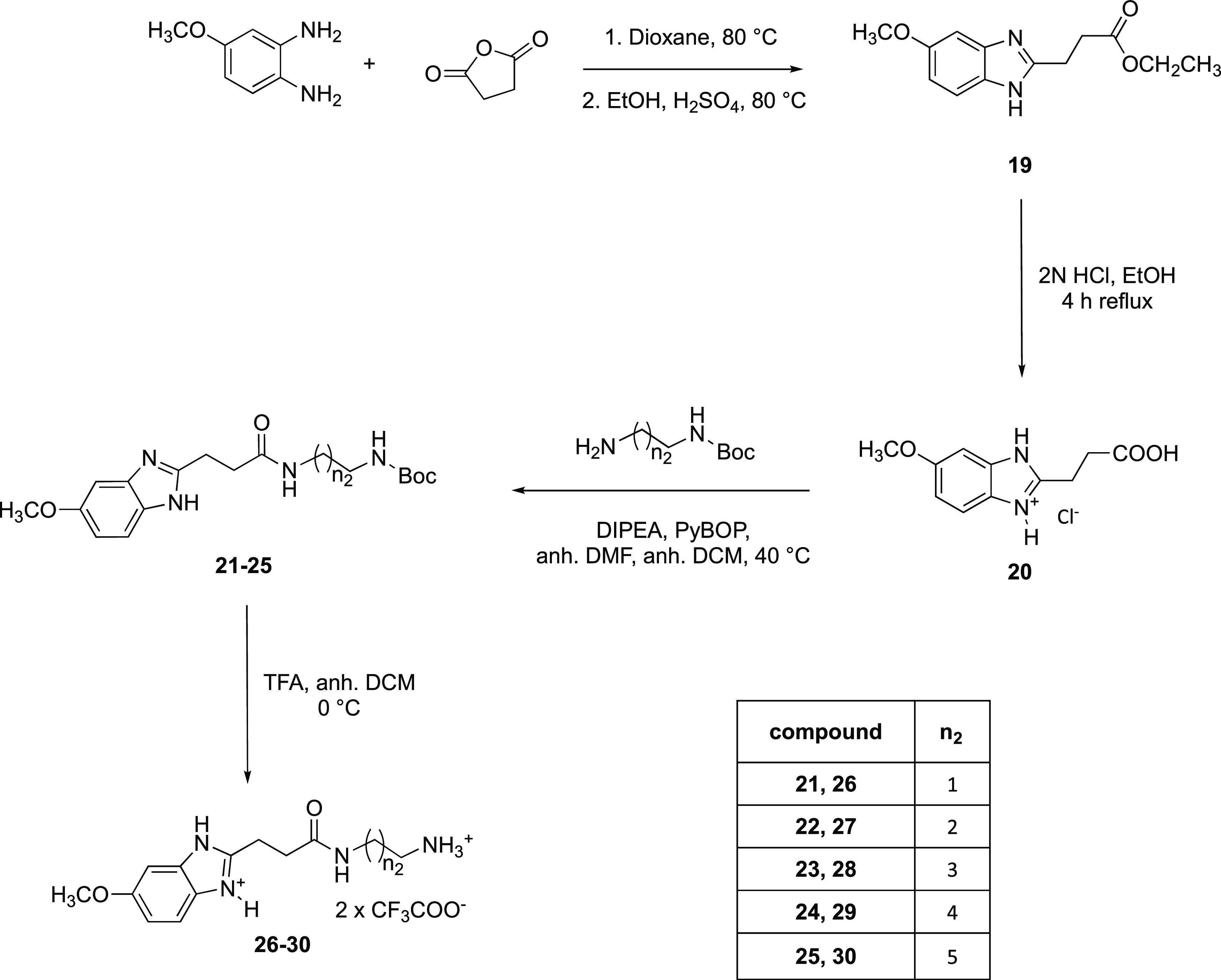
Synthetic Pathway for the 5-Methoxy-1*H*-benzo[*d*]imidazole Bis(trifluoroacetate) Building Blocks

The 3-(5-methoxy-1*H*-benzo[*d*]imidazol-2-yl)propanoic acid (**20**) acted as an educt for the syntheses of dimers **31**, **32**, **34**, **36**, and **38**. To obtain this synthon (Scheme 3), 4-methoxyphenylenediamine was reacted with succinic anhydride to the benzimidazole, followed by esterification with EtOH (→**19**) as already described by Zeng et al.^37^ The last step was necessary for a better separation from side products. Subsequently, **19** was hydrolyzed under acidic conditions (2 N HCl in EtOH^38^), yielding **20** as a hydrochloride in quantitative yields.

Amide coupling (PyBOP/DIPEA) of **20** with *N*-Boc-protected diaminoalkanes gained the respective amides **21**–**25** (yields: 43–87%), which were then deprotected (→**26**–**30**) with TFA in quantitative yields.

**Scheme 4:**
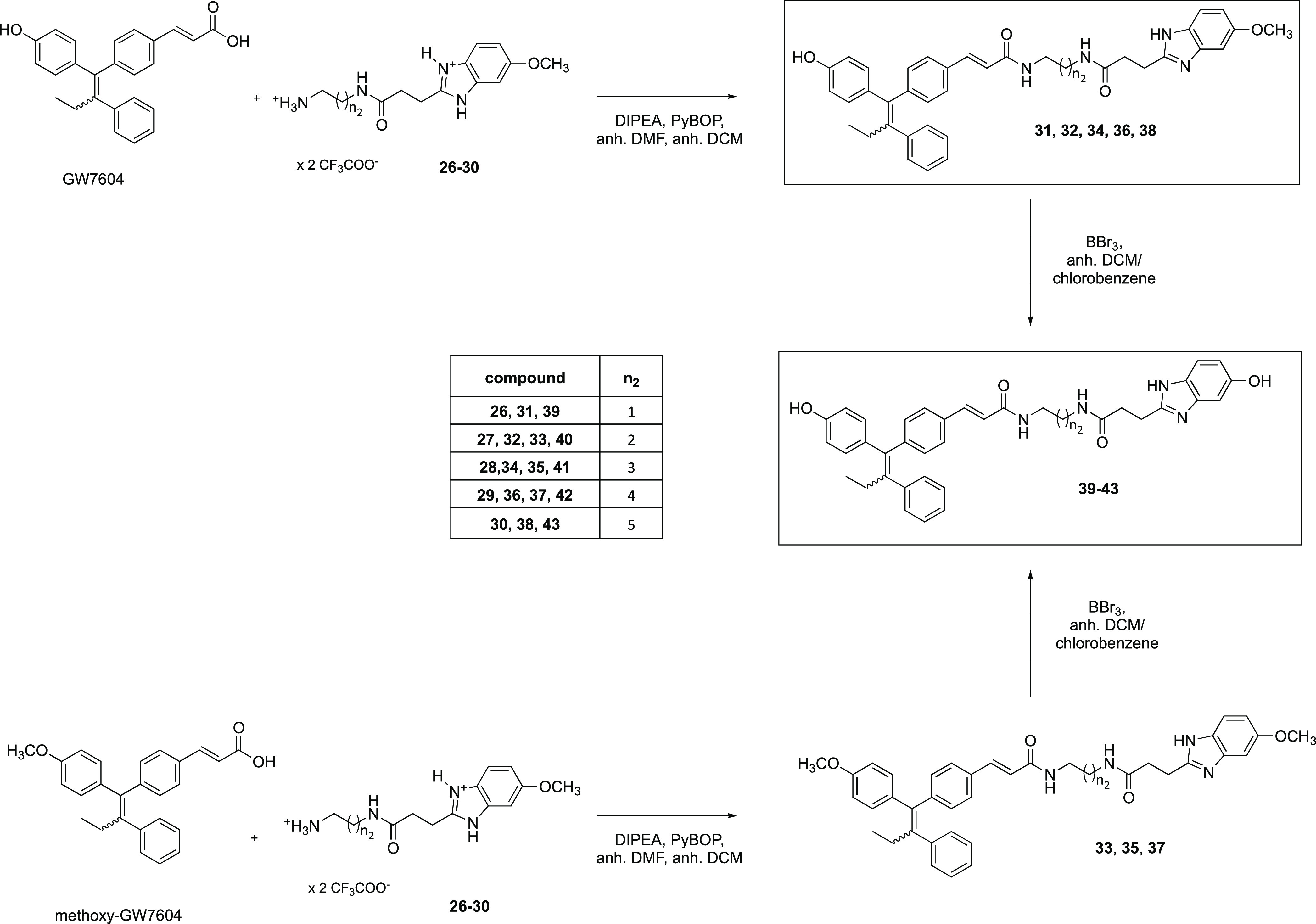
GW7604-Benzimidazole Formation

An analogous reaction of GW7604 with the 5-methoxybenzimidazoles **26**–**30** yielded **31**, **32**, **34**, **36**, and **38** (Scheme 4). Methoxy-GW7604 was used to obtain the methoxy series **33**, **35**, and **37**. The yields for deprotection of compounds **31**–**38** were strongly dependent on their solubility in DCM or chlorobenzene and differed considerably among the homologues (12–81%).

Subsequent cleavage of the methoxy groups with BBr_3_ in dry DCM or chlorobenzene resulted in **39**–**43**.

All final compounds **15**–**18**, **31**, **32**, **34**, **36**, and **38**–**43** were characterized by ^1^H NMR and ^13^C NMR as well as HR-MS. Two-dimensional NMR spectra were recorded to assign the respective isomers. The purity was assessed by high-performance liquid chromatography (HPLC). The syntheses and the characterization of the intermediates as well as 2D NMR spectra of compound **17** as a representative can be found in the Supporting Information (Figures S4–S7).

#### 2.2.2 Stability Studies

The heterodimeric compounds were synthesized from (*E*/*Z*)-GW7604 or (*E*/*Z*)-methoxy-GW7604. The use of isomerically pure educts is pointless, because fast isomerization of the double bond within the 1,1,2-triarylalkene core takes place in solution.^39^ Such a reaction was already observed in the case of GW7604-based homodimers.^24^ Among the new compounds, the benzimidazole derivatives **31**, **32**, **34**, **36**, **39**–**43** as well as **16** were isolated in a 50:50 ratio, while for the others, the *Z* isomer predominated: **15** (*E*/*Z* = 30:70), **17** (*E*/*Z* = 12:88), and **18** (*E*/*Z* = 20:80).

For the interpretation of the biological results, it is necessary to obtain information about the isomerization by simulating physiological conditions. Therefore, we incubated **17** and **36** as examples in a mixture of methanol (MeOH) and 2× phosphate-buffered saline (PBS) (75:25, v/v) and analyzed the *E*/*Z* isomerization by HPLC using an RP18 column and acetonitrile (ACN)/water (TFA, 0.1% or Na_2_SO_4_, 20 mM (pH 3), respectively) gradients.

**Figure 4:**
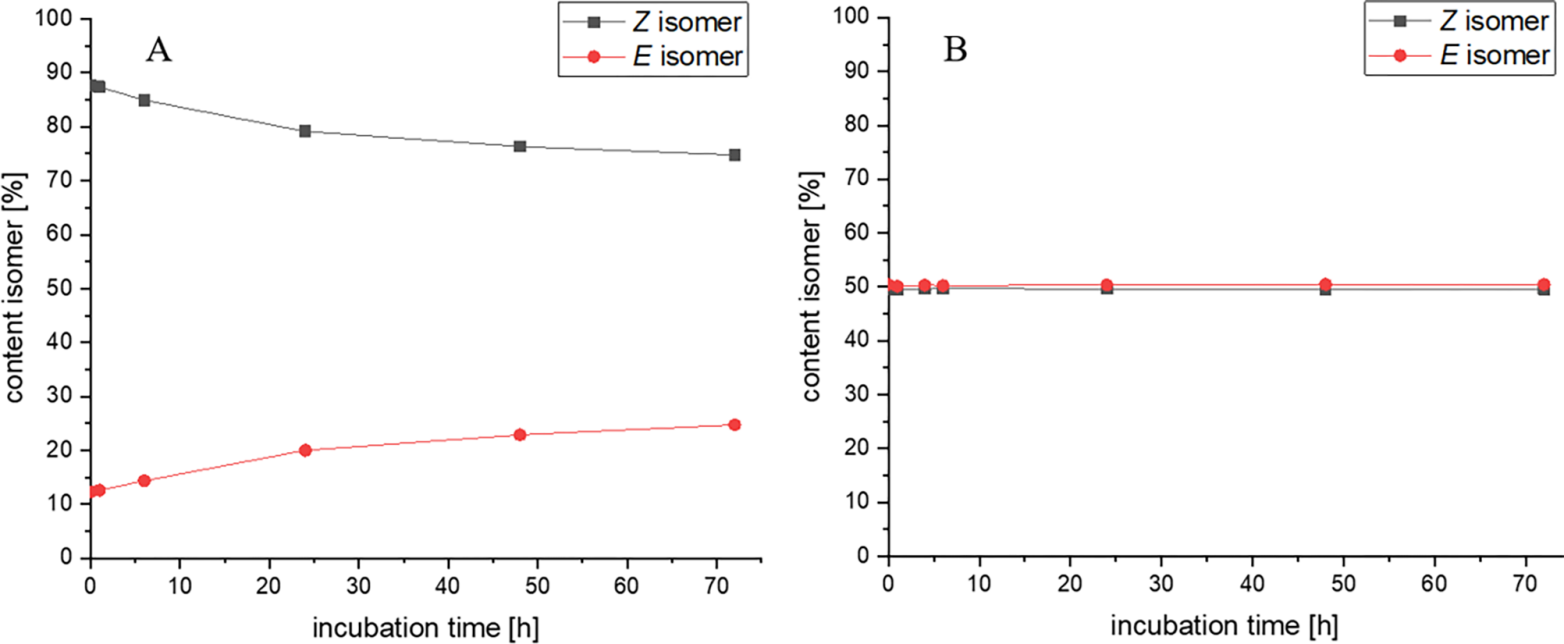
Time-dependent determination of the isomer ratio of **17** and **36** (0.5 mM) in methanol and 2× PBS (75:25, v/v) at 37 °C by HPLC using an RP18 column as the stationary phase. (A) **17** (ACN/water (0.1% TFA) gradient; flow rate: 1.6 mL/min; oven temperature: 30 °C; 254 nm) and (B) **36** (ACN/water (Na_2_SO_4_, 20 mM (pH 3)) gradient; flow rate: 1.2 mL/min; oven temperature: 30 °C; 281 nm).

The *E*/*Z* ratio of **17** (*E*/*Z* = 12:88) built during the reaction course was confirmed. Incubation at 37 °C for 72 h increased the amount of the *E* isomer to 25% (Figure 4A). Compound **36**, incubated under the same conditions, held its *E*/*Z* distribution of 50:50 during the whole experiment (Figure 4B).

These results correspond to studies on the isomerization of (*Z*)-4-OHT to the less active (*E*)-4-OHT. In these experiments, 4-OHT of different isomeric ratios was incubated in various media for 72 h. In each case, a stable ratio of 30% of the *E* isomer was obtained.^40^ In long-time experiments of up to 6 months, 4-OHT isomerized to *E*/*Z* = 50:50, regardless of the applied conditions and solvents.^41^

### 2.3 Biological Evaluation

#### 2.3.1 Ligand Binding Affinity

The relative binding affinity (RBA; compared to estradiol (E2): 100%) was determined with a time-resolved fluorescence resonance energy transfer (TR-FRET) competitive binding assay using the isolated LBDs of ERα and ERβ. GW7604, fulvestrant, and 4-OHT were applied as references.

The affinity of 4-OHT and GW7604 indicates the relevance of the side chain in the β-channel. The RBA values of 4-OHT (RBA(ERα) = 14.7%; RBA(ERβ) = 60.7%) decreased upon the exchange of the dimethylaminoethoxy group by an acrylic acid moiety (→GW7604) to 6.2% (ERα) and 27.1% (ERβ).

The binding affinities listed in Table 1 clearly document that the kind of CABS binder contributes to the affinity of the ERs. All compounds possess equal or higher RBA to ERα than GW7604. The results at ERβ are somewhat sophisticated.

**Table 1:** *In Vitro* Competitive Binding Assay

	compound	TR-FRET^a^ ERα	RBA^b^ ERα	TR-FRET^a^ ERβ	RBA^b^ ERβ
		IC_50_ [nM]	[%]	IC_50_ [nM]	[%]
thioxo-quinazolinones	**15**	4.01 ± 1.90	8.2	12.6 ± 4.8	5.9
	**16**	1.41 ± 0.46	23.4	0.82 ± 0.07	110
	**17**	4.63 ± 1.74	7.1	18.5 ± 4.8	4.0
	**18**	4.23 ± 1.12	7.8	6.81 ±1.86	10.9
5-methoxybenzimidazole	**31**	2.75 ± 1.33	12.0	4.88 ± 2.65	15.2
	**32**	4.11 ± 1.88	8.0	3.01 ± 4.00	24.7
	**34**	2.22 ± 0.76	14.9	5.26 ± 3.10	14.1
	**36**	1.56 ± 0.63	21.1	7.84 ± 0.25	9.5
	**38**	2.61 ± 2.25	12.6	5.22 ± 0.65	14.3
5-hydroxybenzimidazole	**39**	1.45 ± 0.37	22.8	1.99 ± 0.88	37.4
	**40**	2.72 ± 1.12	12.1	3.78 ± 1.54	19.7
	**41**	1.62 ± 0.50	20.4	2.84 ± 1.31	26.2
	**42**	1.28 ± 0.58	25.7	3.81 ± 2.35	19.5
	**43**	3.41 ± 2.07	9.7	7.8 ± 2.30	9.5
references	E2	0.33 ± 0.19	100	1.02 ± 0.50	100
	GW7604	5.35 ± 1.39	6.2	3.77 ± 1.06	27.1
	fulvestrant	2.85 ± 0.83	11.6	10.2 ± 2.5	10.0
	4-OHT	2.24 ± 0.89	14.7	1.68 ± 1.00	60.7

a Displacement of fluorescent-labeled E2 from the LBD of ERα or ERβ by heterodimeric compounds and references.

b Relative binding affinity (RBA) compared to E2 (100%).

The long and flexible spacer in **16** allows an effective attachment of the linked CABS binder at the proposed binding caves, especially at the surface of ERβ (Figure 2B). The cation−π contacts of the aromatic ring with the charge clamp residues Lys314 and Gln327 (Figure 2B) seem to be the key interaction responsible for the high binding affinity of 110%. Such a binding appears to be also relevant at ERα (RBA = 23.4%). The predicted attachment of **15** (Figure 2A) is less effective. The short alkyl chain mediates only nonspecific van der Waals interactions within the hydrophobic pocket closer to the LBS, resulting in distinctly lower RBA values (RBA(ERβ) = 5.9%; RBA(ERα) = 8.2%). Compound **17** bearing the 4-(4-(4-(4-oxo-2-thioxo-1,4-dihydroquinazolin-3(2*H*)-yl)butanoyl)piperazin-1-yl)phenyl residue caused an RBA at ERβ of 4.0%, indicating that this moiety is not able to reach its essential binding cave.^25^ Elongation of the spacer between piperazine and 2-thioxo-1,4-dihydroquinazoline from C3 to C7 also failed to increase the ER affinity (**18**: RBA(ERβ) = 10.9%). A binding pose as depicted in Figure 2C seems not to be suitable to strengthen the attachment at the ER.

The RBA values of 7.1–8.2% determined for **15**, **17**, and **18** at ERα indicate comparable interactions at both subtypes. Only the RBA of **16** is lower at ERα (23.4%) compared to ERβ (110%). However, the binding affinity to ERα is still higher than that of 4-OHT and GW7604.

The terminal benzimidazole represents an efficient CABS binder. The 5-methoxy derivatives **31**, **34**, and **38** showed relatively high binding affinities without subtype selectivity (RBA(ERα) = 12.0–14.9%; RBA(ERβ) = 14.1–15.2%). In contrast, **32** more effectively bonded to ERβ (RBA = 24.7%) than to ERα (RBA = 8.0%). The effect of compound **36** is controversial. The RBA amounts to 21.1% (ERα) and 9.5% (ERβ). These findings contradict in part the results of the theoretical considerations, which documented a preference of the compounds with the C6 spacer (**38**) at ERβ.

The 5-hydroxybenzimidazoles **39** (RBA = 37.4%), **41** (RBA = 26.2%), and **42** (RBA = 19.5%) possessed higher receptor bindings to ERβ than their 5-methoxy derivatives. As predicted, the additional H-bond formation to Gln327 (Figure 3A) increased the affinity. **40** (RBA = 19.7%) and **43** (RBA = 9.5%) were slightly less potent after ether cleavage. The same trend was observed at ERα. The highest RBA values were observed for **39** (22.8%), **41** (20.4%), and **42** (25.7%).

In conclusion, the subtype selectivity regarding the interaction with the isolated receptor of the heterodimeric GW7604 derivatives was low. At ERα, only **36** (ERα/ERβ = 2.22) as well as **16** and **32** at ERβ (ERβ/ERα = 4.7) possessed higher than a 2-fold selectivity. It is noteworthy that the binding compared to GW7604 was improved for ERα, while it was the same (with the exception of **16**) for ERβ.

#### 2.3.2 Coactivator Recruitment

To assess the ability of the heterodimeric ligands to induce or inhibit coactivator binding, the interaction of the modified peroxisome proliferator-activated receptor-γ coactivator 1 (PGC-1) peptide with the LBD of ERα was studied by a TR-FRET assay with **16**, **36**, and **42** as examples, which possess the highest binding affinity.

E2 as a positive control caused maximum coactivator recruitment already at the concentration of 10 nM (for details, see the Supporting Information; Figure S33). In contrast, none of the compounds showed *per se* agonistic effects up to a concentration of 1 μM.

The ability to prevent the activating effects of E2 at ERα was evaluated in a competition experiment (E2: 4 nM, representing the concentration that activated the coactivator recruitment to 80% of the maximum; drug concentrations: 5 nM to 3 μM) after an incubation time of 10 or 30 min.

**Figure 5:**
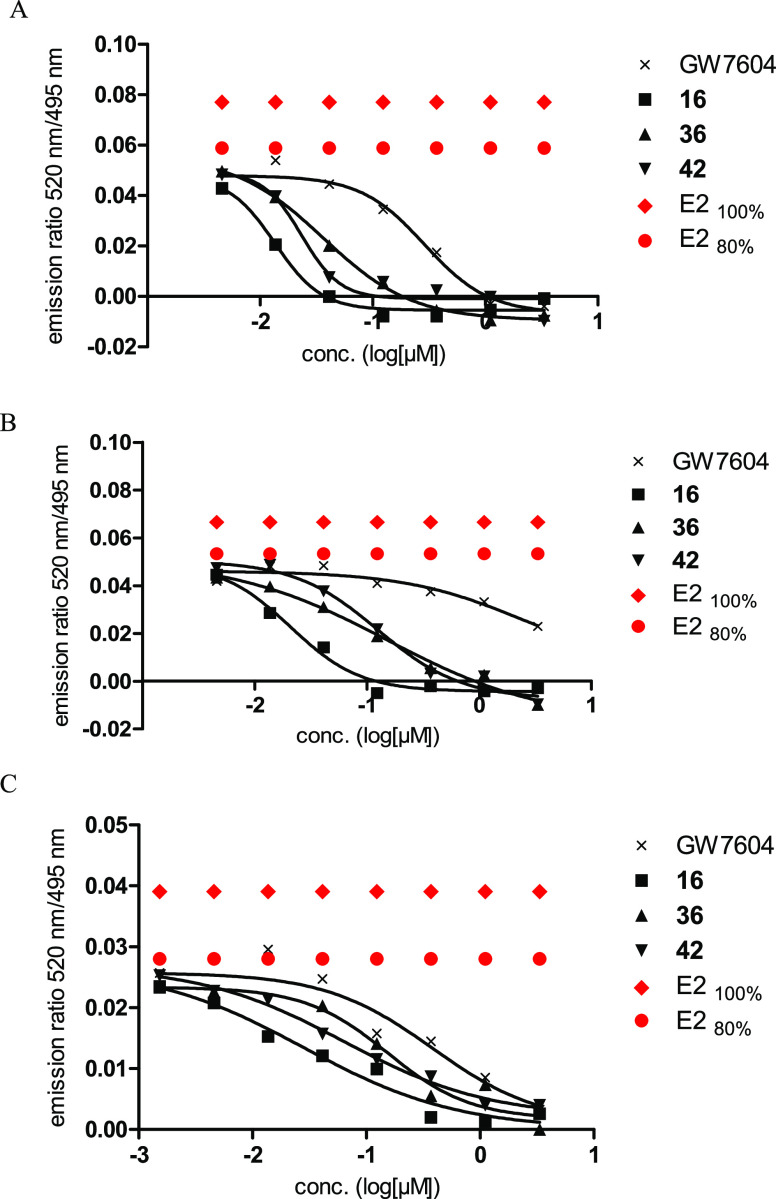
Inhibition of coactivator recruitment by concomitant administration of compounds **16**, **36**, and **42** with E2 (ERα: 4 nM; ERβ: 34 nM). (A) ERα, incubation time: 10 min; (B) ERα, incubation time: 30 min; and (C) ERβ, incubation time: 30 min.

GW7604 (1 μM) completely reduced the PGC-1 binding at ERα after 10 min of incubation. This effect was strongly diminished after 30 min. Even at 5 μM, merely 45% coactivator binding was observed (Figure 5).

Compounds **16**, **36**, and **42** effectively prevented coactivator recruitment. The activity after 10 min at ERα increased in the series GW7604 (IC_50_ = 228.4 nM) < **36** (IC_50_ = 36.33 nM) < **42** (IC_50_ = 23.34 nM) < **16** (IC_50_ = 13.54 nM) (Figure 5A). The derivatives completely circumvented the attachment of PGC-1 even after a prolonged incubation of 30 min GW7604 (IC_50_ = 2433 nM) < **36** (IC_50_ = 153.1 nM) < **42** (IC_50_ = 125.3 nM) < **16** (IC_50_ = 20.88 nM) (Figure 5B). The thioxo- quinazolinone dimer **16** showed the strongest effects after 10 and 30 min. In contrast to the other compounds, its effect only marginally diminished with time. Furthermore, it was 100-fold more active than GW7604. This might be the consequence of the additional addressing of the CABS, which seems to be more effective in the case of the thioxo-quinazolinone than in the case of terminal benzimidazoles.

The inhibition of coactivator recruitment was also investigated at ERβ (E2-concentration: 34 nM) (Figure 5C). In contrast to the experiment with ERα, GW7604 caused a complete repression of PGC1 binding at 3 μM after an incubation time of 30 min (IC_50_ = 382.9 nM). **16** (IC_50_ = 28.34 nM) and **36** (IC_50_ = 154.4 nM) showed comparable effects at both subtypes, while the inhibitory potency at ERβ strongly increased to IC_50_ = 63.97 nM in the case of **42**.

These findings point out that the dimers were capable of inhibiting the coactivator binding to the receptor not only by the blockage of the LBS but also, more importantly, by the spatial isolation of the CABS.

#### 2.3.3 Solubility and Cellular Uptake

Prior to discussing the effects in cellular systems, the solubility of the compounds at relevant concentrations was estimated. The inherent fluorescence of the cinnamide scaffold is suitable for quantification not only in water but also in cellular systems by fluorometric measurements.

The solubility in aqueous solutions is, with the exception of **36** (∼10 μM), higher than 20 μM: thioxo-quinazolinones: **15**: >40 μM; **16**: >40 μM; **17**: 22.3 μM; **18**: >40 μM; 5-methoxybenzimidazoles: **31**: 17.3 μM; **32**: 25.2 μM; **34**: 25.2 μM; **38**: 25.9 μM; 5-hydroxybenzimidazoles: **39**–**43**: >40 μM. It is worth mentioning that the solubility of fulvestrant is distinctly lower (11.1 μM).

Based on their fluorometric properties, **15**, **34**, and **41** were chosen as representatives for the cellular uptake studies.

The compounds were incubated at a concentration of 10 μM with either MCF-7 or COS-7 cells, and the intracellular amount, determined by fluorometry, was related to the protein content.^42^

**Figure 6:**
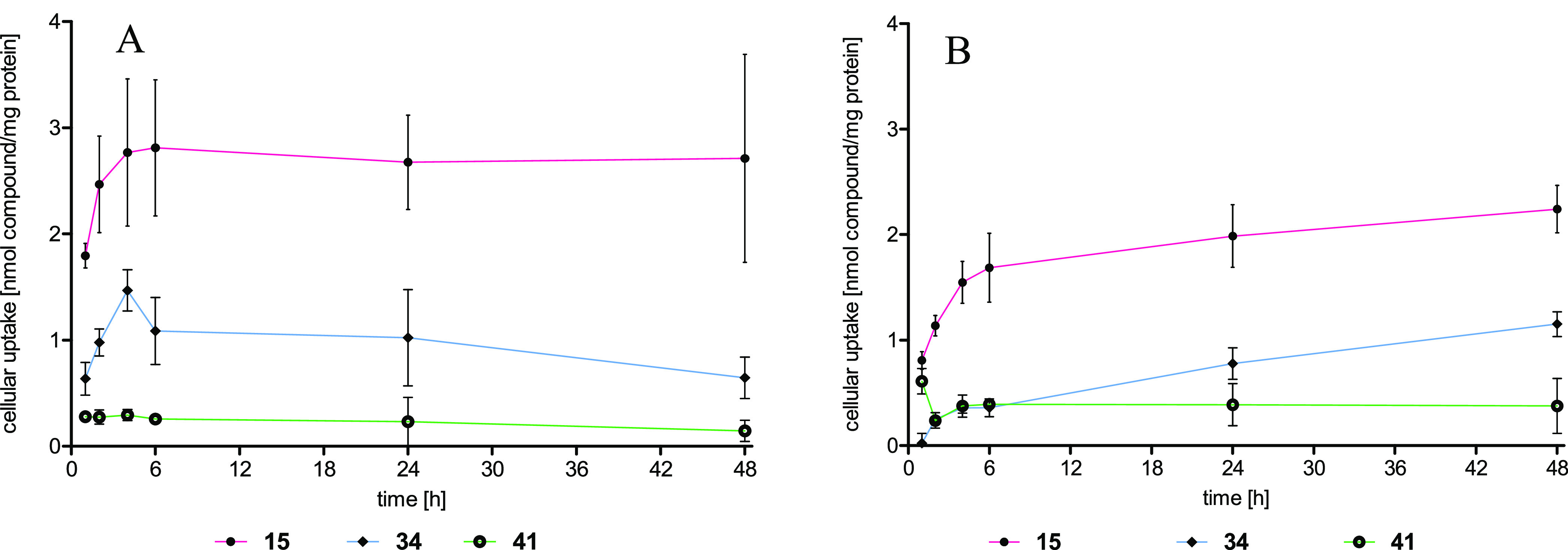
Cellular uptake expressed as nmol compound/mg protein into (A) MCF-7 cells and (B) COS-7 cells after 1, 2, 4, 6, 24, and 48 h of incubation. Data points represent the mean ± SD of ≥3 independent experiments.

The uptake in MCF-7 cells reached saturation already after 4 h (**15** (2.8 nmol/mg), **34** (1.5 nmol/mg), and **41** (0.3 nmol/mg)), which remained constant for the duration of 48 h of incubation (Figure 6A). In COS-7 cells, a comparable kinetic was observed (Figure 6B). However, the uptake of **34** (0.4 nmol/mg) after 4 h was reduced, whereas **15** (1.6 nmol/mg) and **41** (0.4 nmol/mg) accumulated to the same extent as in MCF-7 cells. Furthermore, it is obvious that ether cleavage strongly reduced the intracellular amount, which can be attributed to the higher polarity of the CABS binder. The comparison with GW7604 is impossible because its relative fluorescence intensity is too weak to perform uptake studies.

#### 2.3.4 Inhibition of Transactivation

The interaction of the heterodimeric compounds with the ERs in cellular systems was studied in a luciferase-based reporter gene assay. Thereto, U2OS osteosarcoma cells were transiently transfected with a receptor plasmid (pSG5-ERα or pSG5-ERβ), a firefly reporter plasmid (p(ERE)2-luc^+^), and a renilla plasmid (pRenilla-CMV) for standardization.^24^ Depending on the experimental setting, it is possible to define the agonistic and the antagonistic activity.

At concentrations of 0.1 and 1 μM, none of the compounds caused agonistic effects (Figure S36; Supporting Information). Applied at increasing concentrations (1 nM–10 μM) together with E2 (0.03 nM at ERα, 0.3 nM at ERβ), the antagonistic potency was quantified and expressed as IC_50_ values (Table 2).

**Table 2:** Inhibition of E2-Induced Transactivation Determined in a Luciferase Reporter Gene Assay (ERα and ERβ) Using U2OS Cells, Transiently Transfected with Plasmids pSG5-ERα or pSG5-ERβ and the Reporter Plasmid p(ERE)2-luc^+^

	compound	transactivation ERα	transactivation ERβ
		IC_50_^a^ [nM]	IC_50_^a^ [nM]
thioxo-quinazolinones	**15**	30.2 ± 14.3	4.1 ± 0.6
	**16**	18.5± 1.8	7.5 ± 1.3
	**17**	104 ± 14	48.0 ± 12.0
	**18**	914 ± 50	157± 22
5-methoxybenzimidazole	**31**	9.7 ± 8.9	6.7 ± 2.7
	**32**	21.5 ± 10.7	26.9 ± 5.7
	**34**	34.5 ± 3.5	11.6 ± 5.5
	**36**	9.2 ± 3.2	8.9 ± 4.0
	**38**	9.4 ± 2.7	9.0 ± 3.3
5-hydroxybenzimidazole	**39**	26.0 ± 6.6	20.2 ± 8.6
	**40**	80.8 ± 16.4	18.7 ± 8.3
	**41**	107 ± 4	15.9 ± 1.7
	**42**	30.9 ± 8.7	46.5 ± 20.7
	**43**	20.0 ± 3.2	16.1 ± 7.0
references	GW7604	238 ± 74	154 ± 53
	fulvestrant	n.d.^b^	n.d.^b^
	4-OHT	2.3 ± 1.3	1.0 ± 1.3
	**44**	5.3 ± 3.2	3.6 ± 1.4

a IC_50_ values represent the means ± SD of ≥3 independent experiments,.

b n.d., not defined.

The suitability of the test was verified on the effects of 4-OHT. It reduced the E2-induced luciferase expression with IC_50_ = 2.3 nM (ERα) and 1.0 nM (ERβ), comparable to data from the literature.^43^ Fulvestrant completely inhibited the E2-stimulated luciferase expression even at the lowest concentration (0.05 nM) due to its extraordinarily high ER-downregulation potency (see below). Hence, no IC_50_ calculation was possible.

The reference GW7604 was distinctly less active than 4-OHT with IC_50_ = 238 nM (ERα) and 154 nM (ERβ). All heterodimers, except for **18**, possessed a higher antagonistic activity at both subtypes than GW7604.

Within the thioxo-quinazolinone series, compound **16** was the most potent antagonist at ERα with IC_50_ = 18.5 nM. At ERβ, **15** and **16** showed IC_50_ values of 4.1 and 7.5 nM, respectively, which point to effects independent of the diaminoalkane spacer length (**15**: C3; **16**: C7). On the other hand, the bulky phenylpiperazine moiety strongly reduced the transactivation activity (**17**: ERα: IC_50_ = 104 nM; ERβ: IC_50_ = 48.0 nM; **18**: ERα: IC_50_ = 914 nM; ERβ: IC_50_ = 157 nM).

The most active compounds of the 5-methoxybenzimidazole series, and in general, were **31**, **36**, and **38** inhibiting the stimulating effects of E2 at both ER subtypes with IC_50_ of 6.7–9.7 nM. **32** (ERα: IC_50_ = 21.5 nM; ERβ: IC_50_ = 26.9 nM) and **34** (ERα: IC_50_ = 34.5 nM; ERβ: IC_50_ = 11.6 nM) were less active. Among these derivatives, only **34** possessed a slight subtype selectivity for ERβ.

The data revealed that the diaminoalkane spacer plays a subordinate role in the attachment of the 5-methoxybenzimidazole moiety at the ER. The contacts at ERα seem to be mainly of a hydrophobic nature, because ether cleavage reduced the antagonistic effects. IC_50_ values of **39**, **42**, and **43** increased to 20.0–30.9 nM. **40** and **41** were still less active with IC_50_ = 80.8 and 107 nM.

At ERβ, only **39** (IC_50_ = 20.2 nM) and **42** (IC_50_ = 46.5 nM) slightly lost their antagonistic activities. H-bridges to amino acids, e.g., Gln327 or Lys314 as proposed for **42** and **43** by theoretical studies (Figure 3A), appear to be part of the binding mode and strengthened the attachment to the CABS.

This interpretation has to be handled with care in view of the fact that hydroxy-substituted derivatives accumulated in cells to a lesser amount compared to the related methoxy-bearing compounds (as demonstrated with the examples of **34** and **41**). This explains the weaker antitransactivation potency despite a similar inhibition of coactivator recruitment in the case of benzimidazoles. The thioxo-quinazolinones (e.g., **15**) showed an even higher uptake than benzimidazoles (Figure 6). However, compound **16**, which displayed the strongest inhibition of coactivator recruitment and a binding affinity in the low nanomolar range, did not reflect this inhibitory potency in the reporter gene assay.

#### 2.3.5 Estrogen Receptor Downregulation

**Figure 7:**
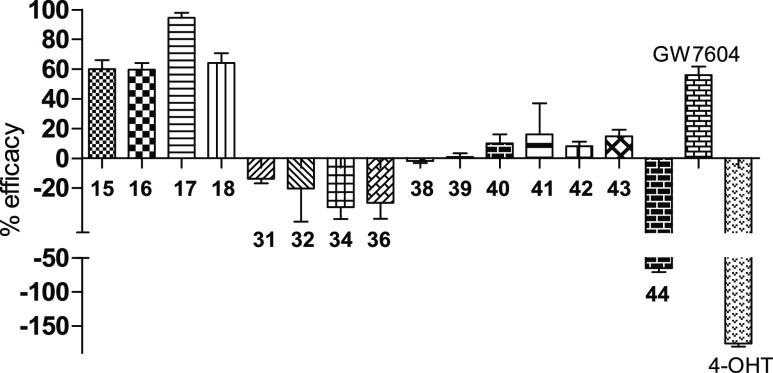
Percent efficacy related to fulvestrant. Negative values indicate upregulation. Values represent the means ± SE of ≥3 independent experiments.

To evaluate the SERM-/SERD-like properties of the heterodimers, their impact on the ERα levels in MCF-7 cells after 24 h of incubation at 1 μM was quantified using an In-Cell Western immunoassay (Table 3 and Figure 7).

The SERD fulvestrant caused an almost complete ER destabilization/degradation (efficiency at 1 μM was set to 100%), while 4-OHT as mixed agonist/antagonist^44,45^ significantly upregulated the ERα content to 263% compared to the untreated control (100%). As already discussed in a previous paper,^24^ the exchange of the dimethylaminoethoxyphenyl group by a cinnamic acid moiety (GW7604) increased the downregulatory properties to an efficiency of 56% (Table 3).

**Table 3:** ERα Levels in MCF-7 Cells Determined by the In-Cell Western Immunoassay

	compound	% ERα remaining^a^ [1 μM]	% efficacy^b^ [1 μM]
thioxo-quinazolinone	**15**	39.9 ± 8.5	60
	**16**	40.3 ± 8.6	60
	**17**	5.5 ± 5.0	95
	**18**	35.7 ± 11.3	64
5-methoxybenzimidazole	**31**	113.8 ± 4.3	c
	**32**	120.3 ± 31.6	c
	**34**	132.9 ± 14.1	c
	**36**	130.0 ± 15.1	c
	**38**	101.8 ± 1.9	c
5-hydroxybenzimidazole	**39**	99.2 ± 3.5	1
	**40**	90.0 ± 10.4	10
	**41**	83.8 ± 29.2	16
	**42**	92.0 ± 5.5	8
	**43**	85.2 ± 6.2	15
references	GW7604	44.3 ± 12.1	56
	fulvestrant	0	100
	4-OHT	263.0 ± 9.0	c
	**44**	165.0 ± 4.0	c

a ERα levels compared to the solvent control (dimethyl sulfoxide (DMSO)). Values represent the mean ± SD of ≥3 independent experiments.

b Efficacy, calculated as the downregulation of ERα compared to the efficacy of the reference compound fulvestrant.

c Upregulation.

This effect was attributed to the change of a positively charged side chain in the case of 4-OHT to a negative one in GW7604. The side chain of 4-OHT interacts with the surface amino acid Asp351 of ERα in an ionic attraction, allowing the binding of coactivators that are necessary for expression of the receptor. The carboxylate of GW7604 generates a strong repulsion of Asp351, which disrupts the surface charge around this amino acid required for coactivator binding in the 4-OHT/ER complex.^19^

The relevance of the positively charged side chain documented the derivation of the carboxylic group of GW7604 with a 1,2-diaminoethane chain. Compound **44** increased the ERα expression to about 165%, resembling the biological profile of the partial agonist 4-OHT.^18,24,46,47^

The binding of the long side chain at the steroidal core of fulvestrant to the surface of the receptor effectively destabilizes the protein, leading to ubiquitination and degradation. Accordingly, in the In-Cell Western immunoassay, a complete downregulation of ERα in MCF-7 cells (efficacy 100%) is visible.

Exceptional results provided the testing of the thioxo-quinazolinones. All of them were more active downregulators than the parent compound GW7604, although they do not bear a negative charge at the side chain. Furthermore, based on their structure, they cannot change the conformation of the ER as it is caused by the side chain of fulvestrant. Nevertheless, **15**, **16**, and **18** reduced the ERα content to about 40%, while **17** (efficacy: 95%) was almost as active as fulvestrant.

Compounds out of the benzimidazole series only marginally influenced the ERα content in the cells. Upon incubation with the 5-methoxy derivatives at a drug concentration of 1 μM, the level slightly increased (100–130%), while the hydroxy-substituted ones showed a trend toward downregulation (83–100%) (Figure 7).

The mode of downregulation with compounds bearing the thioxo-quinazolinone scaffold has not yet been completely clarified.

#### 2.3.6 Antiproliferative Effects

**Figure 8:**
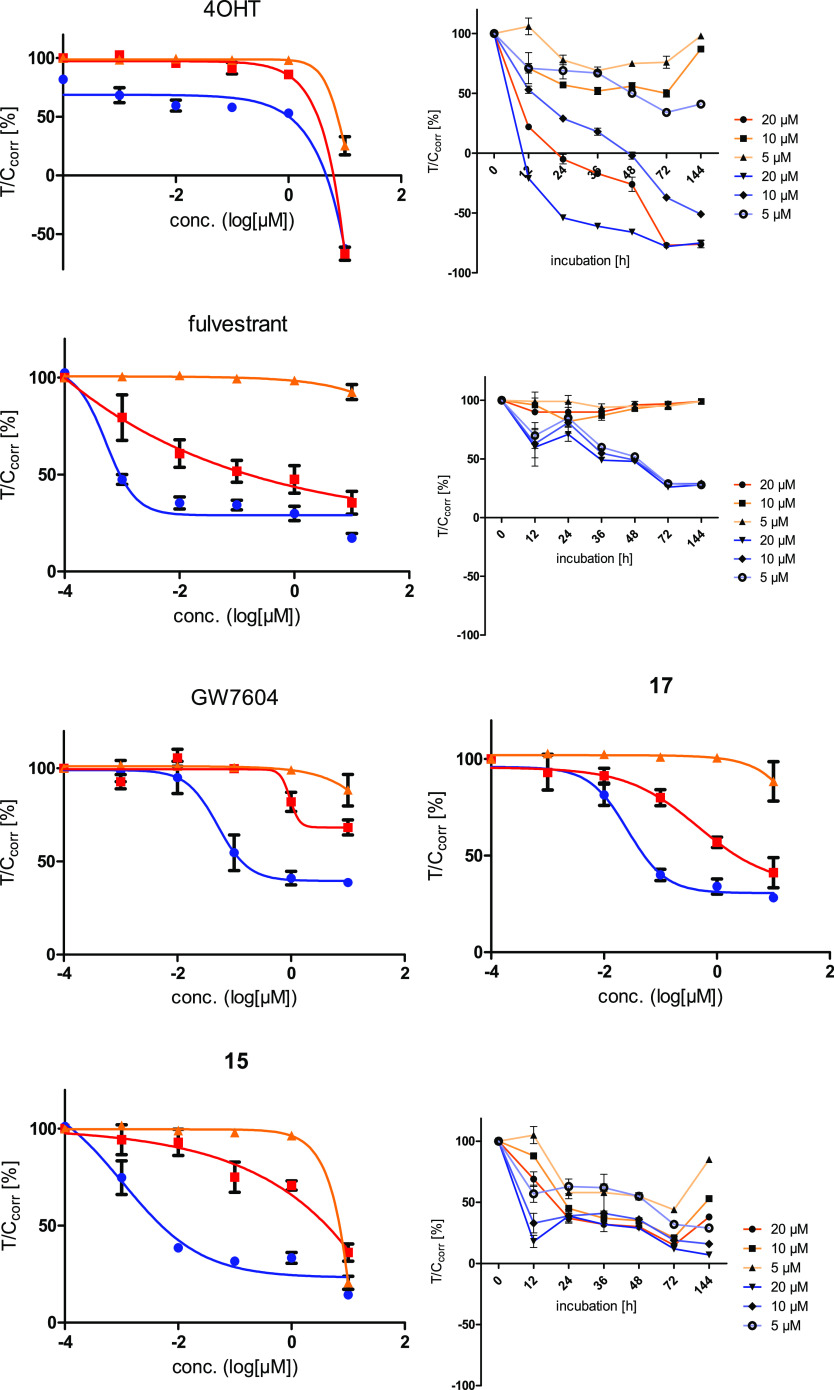

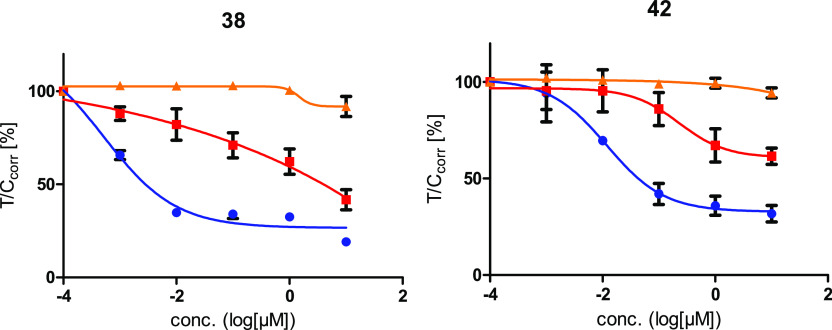
Antiproliferative effects against ER-positive MCF-7 (blue), tamoxifen-resistant MCF-7TamR (red), and ER-negative MDA-MB-231 (orange) breast cancer cells. Values represent the mean ± SD of ≥3 independent experiments. For further graphs, see the Supporting Information (Figures S34 and S35).

Whether the ER interactions discussed above consequently influence the proliferation of hormone-dependent tumor cells was assessed *via* the crystal violet assay.^48^ The ER-positive MCF-7, the tamoxifen-resistant MCF-7TamR,^49^ as well as the hormone-independent MDA-MB-231 breast cancer cell lines were used to visualize these effects. Representative concentration–activity and time–activity curves are depicted in Figure 8. All calculated IC_50_ values are listed in Table 4.

**Table 4:** Antiproliferative Effects: IC_50_ Values at ER-Positive MCF-7, Tamoxifen-Resistant MCF-7TamR, and ER-Negative MDA-MB-231 Breast Cancer Cells

	compound	MCF-7	IC_50_ [nM]^a^	MDA-MB-231
			MCF-7TamR	
thioxo-quinazolinone	**15**	1.9	2 296	>1 μM
	**16**	4.2	476.6	n.a.
	**17**	27.8	550.8	n.a.
	**18**	12.6	1 208	n.a.
5-methoxybenzimidazole	**31**	2.8	n.d.	n.a.
	**32**	2.4	n.d.	n.a.
	**34**	4.1	486.3	n.a.
	**36**	1.8	68.7	n.a.
	**38**	0.5	n.d.	n.a.
5-hydroxybenzimidazole	**39**	10.6	531.0	n.a.
	**40**	4.5	543.7	n.a.
	**41**	9.8	1 711	n.a.
	**42**	11.5	235.7	n.a.
	**43**	5.6	325.1	>1 μM
references	GW7604	52.0	956.9	n.a.
	fulvestrant	0.58	1.8	n.d
	4-OHT	n.d	n.d	n.d

a Values represent means of ≥3 independent experiments; n.d., not determined; n.a., not active.

The compounds were incubated at concentrations between 1 nM and 10 μM for 120 h, and the remaining adherent growing cells were stained by crystal violet.^24^ The photometric measurement after extraction of the dye from the chromatin with ethanol allows the quantification of the cell mass. Comparing the treated wells with the solvent-treated control wells results in % *T*/*C*_corr_ values, from which conclusions can be drawn about the extent of the cytotoxicity. *T*/*C*_corr_ > 80% is considered nonantiproliferative/nontoxic. *T*/*C*_corr_ values between 80 and 20% are classified as antiproliferative. Below 20%, the compounds are cytostatic. A cytocidal effect is defined as a *T*/*C*_corr_ value below 0%.

To obtain an insight into the kinetic of cell death, MCF-7 and MDA-MB-231 cells were further incubated at concentrations of 5, 10, and 20 μM for 12, 24, 36, 48, 72, and 144 h.

4-OHT did not influence the cells up to a concentration of 1 μM. At 10 μM, cytostatic and cytocidal effects occurred and reduced the *T*/*C*_corr_ values of MDA-MB-231, MCF-7, and MCF-7TamR cells to 10, −55, and −57%, respectively. Time-dependent experiments at increased concentrations also indicated cytocidal effects against the MCF-7 cell line after 24 h at 20 μM and after 48 h at 10 μM. The growth of MDA-MB-231 cells was only reduced at 20 μM, reaching cytocidal effects after 24 h (Figure 8).

Fulvestrant was completely inactive in the MDA-MB-231 cell line, while it had strong effects in MCF-7 cells (IC_50_ = 0.58 nM). The time–activity curve (Figure 8) indicates no unspecific cytotoxicity but identical curves at concentrations of 5, 10, and 20 μM with a maximum of activity of *T*/*C*_corr_ = 26% after 72 h. The proliferation of MCF-7TamR cells was reduced, too (IC_50_ = 1.8 nM).

It should be mentioned that the concentration–activity curves showed only antiproliferative effects. The maximum activity is in the range of *T*/*C*_corr_ = 30–35%. The compounds displayed no cytostatic or cytocidal effects at the applied concentrations.

GW7604 did not influence MDA-MB-231 cells but reduced the growth of MCF-7 cells with IC_50_ = 52.0 nM. Against MCF-7TamR only at 10 μM, a marginal effect of *T*/*C*_corr_ = 68% was observed. Treatment of MDA-MB-231 cells with the heterodimeric compounds did not affect the proliferation up to a used concentration of 1 μM. At higher concentrations (10 μM), only **15** and **43** reduced the cell growth unspecifically to *T*/*C*_corr_ = 20 and 63%. This effect seems to be hormone/ER-independent, because the time-dependent experiment documented, e.g., for **15** at concentrations higher than 1 μM identical effects against MDA-MB-231 and MCF-7 cells (Figure 8).

The activity in the MCF-7 cell line depended on the used CABS binder and the kind of linkage to the GW7604 core. The thioxo-quinazolinone derivatives **15** and **16** caused IC_50_ values of 1.9 and 4.2 nM, while the piperazinylbenzoate bearing compounds **17** and **18** showed decreased cytotoxicity with IC_50_ = 27.8 and 12.6 nM.

The 5-methoxybenzimidazoles **31**–**38** reduced the cell growth of MCF-7 cells independent of the spacer length. The IC_50_ values were in the range of 0.5–4 nM, only marginally higher than the IC_50_ of fulvestrant (IC_50_ = 0.58 nM). **38** (IC_50_ = 0.50 nM) was even as active as fulvestrant. Ether cleavage diminished the cytotoxicity to IC_50_ = 4.5–11.5 nM. The most active compound was **40** with an IC_50_ of 4.5 nM.

It should be mentioned that none of the compounds stimulated the proliferation of hormone-dependent MCF-7 cells, indicating the absence of partial estrogenic properties.

All compounds were active against the MCF-7TamR cell line, showing a flat concentration–activity curve. The maximum effect at 10 μM was in most cases higher than *T*/*C*_corr_ = 50%. Only **15**, **17**, **38**, and **43** reached a *T*/*C*_corr_ of about 40%. All compounds were more active than GW7604. Sigmoid curves allowed in some cases the calculation of IC_50_ values as a parameter of antiproliferative potency. From these data, it is obvious that the compounds were less potent than fulvestrant. The most active compound was **36** with an IC_50_ of 68.7 nM.

## 3 Conclusions

In this SAR study, GW7604 was linked to known CABS binders to evaluate the possibility of increasing ER binding and to inhibit coactivator recruitment. The biological activities were investigated at the isolated receptors and in cellular systems. The thioxo-quinazolinones mediated high binding affinity, with extraordinary RBA values of 23.4 and 110% measured for compound **16** at ERα and ERβ, respectively. The 5-methoxy/hydroxybenzimidazole increased the affinity, too. All derivatives showed higher affinity to ERα than GW7604, while only compound **16** and the 5-hydroxybenzimidazole derivative **39** exceeded the affinity of the reference to ERβ. The coactivator recruitment was also effectively inhibited at both receptor subtypes, as demonstrated for **16**, **36**, and **42**, with 10- to 100-fold higher inhibitory potency compared to GW7604. Consequently, the heterodimeric compounds inhibited the E2-induced transactivation in the luciferase reporter gene assay more effectively. None of them stimulated the ER expression as 4-OHT.

ER downregulation showed the thioxo-quinazolinones derivatives. **17** caused nearly complete receptor degradation (94.5% efficacy). In contrast, the receptor content remained almost unchanged in the case of benzimidazole-bearing species.

The influence on cell growth is based on the interference in hormonal pathways. The compounds did not reduce the proliferation of hormone-independent MDA-MB-231 cells. Only compound **15** showed unspecific effects at concentrations higher than 1 μM. Against hormone-dependent MCF-7 cells, all heterodimers were more active than GW7604. Compound **38** (IC_50_ = 0.5 nM) reduced the proliferation as effectively as fulvestrant. Also, the growth of the tamoxifen-resistant subline MCF-7TamR decreased upon treatment, however, with a flat concentration−activity relation.

In conclusion, the attempt to modify the pharmacological profile of GW7604 by linking the cinnamic acid moiety by a diaminoalkane spacer to the CABS binder was successful. The compounds showed the profile either of pure antiestrogens (5-methoxy/hydroxybenzimidazoles) or of pure antiestrogens with ER-degradation potency (thioxo-quinazolinones).

## 4 Experimental Section

### 4.1 Chemistry

#### 4.1.1 General

All reagents were purchased from Sigma-Aldrich, TCI, VWR, or Alfa Aesar and were used without further purification. All solvents were distilled before usage. Anhydrous solvents were obtained by distillation under argon over an appropriate drying agent. Reactions were performed under an inert argon atmosphere using oven-dried glassware, septa, and syringes for the addition of substances. Chromatography purification was performed employing either classic standard procedures or on a Biotage Isolera 1 Flash purification system. Silica gel 60 Å was used in both cases. ^1^H NMR and ^13^C NMR spectra were obtained on a Varian Gemini-200 (now Agilent), 400 MHz Avance 4 Neo (Bruker), 500 MHz Direct Drive 2 (Agilent), 600 MHz Avance II (Bruker), or 700 MHz Avance 4 Neo (Bruker) spectrometer. As solvents for NMR, deuterated chloroform (CDCl_3_), dimethyl sulfoxide (DMSO-*d*_6_), acetone ((CD_3_)_2_CO), and methanol (CD_3_OD) were used. The chemical shifts (δ) were referenced to tetramethylsilane (TMS) or the solvent peak and are given in parts per million (ppm). Coupling constants (*J*) are given in Hertz (Hz).

The purity of final compounds **15**–**18**, **31**, **32**, **34**, **36**, **38**, and **39**–**43** as well as the stability of compounds **17** and **36** was measured by HPLC (Shimadzu, LaChrom) on a 250 mm reversed-phase C18 column as the stationary phase (KNAUER) equipped with a diode-array detector (for HPLC spectra and methods, see the Supporting Information). HR-MS spectra were obtained from an Orbitrap Elite MS (Thermo Fisher Scientific). All final compounds were >95% pure.

#### 4.1.2 Computational Design of the Heterodimers

The ligands were docked analogously to previous studies.^24^ The 2D and 3D chemical structures were generated with the ChemDraw14 package.^50^ GOLD suite v 5.2^51^ was employed for docking experiments and the results were examined in LigandScout 4.1 alpha4.^52^

#### 4.1.3 Synthesis of the Final Compounds

##### 4.1.3.1 General Procedure for Amide Formation

First, DIPEA (6.0 equiv) and then PyBOP (1.1 equiv), dissolved in dry DCM, were added to a solution of GW7604 or methoxy-GW7604 (1.1 equiv) in dry DMF at 0 °C under an argon atmosphere. The solution was stirred for 5 min followed by the dropwise addition of the respective trifluoroacetate salt (1.0 equiv) in dry DMF. The reaction mixture was stirred initially at 0 °C for 30 min and then at 40 °C for 20 h. Subsequently, the solvents were concentrated and the residue was dissolved in ethyl acetate (EA). Water was added (pH = 8–9, adjusted with 2N NaOH if needed), and the aqueous phase was extracted twice with EA. The combined organic layers were washed with brine, dried over anhydrous Na_2_SO_4_, and filtered. Purification was achieved by column chromatography.^32−35^

##### 4.1.3.2 Syntheses of the Thioxo-quinazolinones **15**–**18**

Diamide coupling was carried out according to the general procedure for heterodimer formation, yet at rt instead of at 40 °C and performing an acidic workup with 1 N HCl at pH = 2–3.

##### 4.1.3.2.1 *N*-[3-((*E*)-3-(4-((*E*/*Z*)-1-(4-Hydroxyphenyl)-2-phenylbut-1-enyl)phenyl)acrylamido)propyl]-4-[4-oxo-2-thioxodihydroquinazolin-3-yl]butanamide (**15**)

**15** was synthesized following the general procedure described above, applying 55 mg of GW7604 (0.15 mmol) in 0.5 mL of dry DMF, 0.16 mL of DIPEA (0.94 mmol), and 85 mg of PyBOP (0.16 mmol) in 1 mL of dry DCM. The trifluoroacetate salt **11** (1.1 equiv, 71 mg, 0.17 mmol) in 1.0 mL of dry DMF was added dropwise. Purification was carried out by flash column chromatography with a petroleum ether (PE):EA:MeOH gradient (EA: 30 → 100% and then MeOH: 0 → 5%) followed by column chromatography with a DCM and MeOH gradient (98:2 → 95:5). **15** was obtained as a colorless powder (28 mg, 0.038 mmol, 26%). Purity: 98.2% (HPLC: 1.0 mM in MeOH, 254 nm). ^1^H NMR: (700 MHz, CD_3_OD, *E*/*Z* = 30:70): δ 0.92 (t, ^*3*^*J* = 7.4 Hz, 3H, CH_2_C*H*_*3*_), 1.71 (p, ^*3*^*J* = 6.8 Hz, 0.6H, NHCH_2_C*H*_*2*_CH_2_NH, *E* isomer), 1.76 (p, ^*3*^*J* = 6.8 Hz, 1.4H, NHCH_2_C*H*_*2*_CH_2_NH, *Z* isomer), 2.07–2.15 (m, 2H, NCH_2_C*H*_*2*_CH_2_CONH), 2.28–2.65 (m, 2H, NCH_2_CH_2_C*H*_*2*_CONH), 2.47 (q, ^*3*^*J* = 7.4 Hz, 1.4H, C*H*_*2*_CH_3_, *Z* isomer), 2.51 (q, ^*3*^*J* = 7.4 Hz, 0.6H, C*H*_*2*_CH_3_, *E* isomer), 3.20 (t, ^*3*^*J* = 6.7 Hz, 0.6H, NHCH_2_CH_2_C*H*_*2*_NH, *E* isomer), 3.24 (t, ^*3*^*J* = 6.7 Hz, 1.4H, NHCH_2_CH_2_C*H*_*2*_NH, *Z* isomer), 3.33–3.38 (2 × t, 2H, 2 × NHC*H*_*2*_CH_2_CH_2_NH), 4.54–4.61 (2 × t, 2H, 2 × NC*H*_*2*_CH_2_CH_2_CONH), 6.36–6.46 (m, 1.6H, Ar*H* + CH=C*H*CONH), 6.60 (d, ^*3*^*J* = 15.8 Hz, 0.7H, CH=C*H*CONH, *Z* isomer), 6.66 (d, ^*3*^*J* = 8.6 Hz, 1.4H, Ar*H*, *Z* isomer), 6.78 (d, ^*3*^*J* = 8.5 Hz, 0.6H, Ar*H*, *E* isomer), 6.87 (d, ^*3*^*J* = 8.2 Hz, 0.6H, Ar*H*, *E* isomer), 7.03 (d, ^*3*^*J* = 8.5 Hz, 0.6H, Ar*H*, *E* isomer), 7.07–7.20 (m, 5.7H, Ar*H*), 7.20–7.27 (m, 2.4H, Ar*H*), 7.27–7.32 (m, 1H, Ar*H*), 7.35 (d, ^*3*^*J* = 15.7 Hz, 0.3H, C*H*=CHCONH, *E* isomer), 7.50–7.58 (m, 2.1H, Ar*H* + C*H*=CHCONH), 7.63–7.70 (m, 1H, Ar*H*), 7.99–8.07 (m, 1H, Ar*H*). ^13^C NMR: (176 MHz, CD_3_OD, *E*/*Z* = 30:70): δ 13.81, 13.87, 24.36, 29.86, 29.99, 30.18, 30.22, 34.57, 37.90, 37.94, 38.04, 38.11, 46.80, 115.28, 116.03, 116.13, 117.08, 121.07, 121.54, 125.49, 127.21, 127.37, 127.86, 128.68, 128.83, 128.93, 129.00, 130.84, 130.86, 131.10, 131.67, 132.44, 133.05, 133.56, 134.64, 135.29, 135.60, 136.56, 139.55, 139.69, 140.74, 141.47, 142.84, 143.64, 143.68, 143.90, 146.78, 147.03, 156.67, 157.56, 161.65, 168.83, 175.42, 177.40. HR-MS (*m*/*z*): calcd for C_40_H_40_N_4_O_4_S [M – H]^−^: 671.2698, found: 671.2720.

##### 4.1.3.2.1.1 *N*-[4-((*E*)-3-(4-((*E*/*Z*)-1-(4-Hydroxyphenyl)-2-phenylbut-1-enyl)phenyl)acrylamido)butyl]-8-[4-oxo-2-thioxodihydroquinazolin-3-yl]octanamide (**16**)

**16** was synthesized following the general procedure applying 56 mg of GW7604 (0.15 mmol) in 0.5 mL of dry DMF, 0.16 mL of DIPEA (0.94 mmol), and 87 mg of PyBOP (0.17 mmol) in 1 mL of dry DCM. **12** (1.1 equiv, 84 mg, 0.17 mmol) in 1.0 mL of dry DMF was added. Purification by flash column chromatography with a gradient of PE and EA (70 → 100% EA) led to **16** as a whitish-yellow powder (31 mg, 0.042 mmol, 29%). Purity: 97.3% (HPLC: 1.0 mM in MeOH, 254 nm). ^1^H NMR: (700 MHz, CD_3_OD, *E*/*Z* = 45:55): δ 0.88–0.93 (2 × t, 3H, 2 × CH_2_C*H*_*3*_), 1.34–1.41 (m, 6H, C*H*_*2*_), 1.53–1.65 (m, 6H, C*H*_*2*_), 1.71–1.78 (m, 2H, C*H*_*2*_), 2.13–2.22 (2 × t, 2H, 2 × C*H*_*2*_CON), 2.45 (q, ^*3*^*J* = 7.3 Hz, 1.1H, C*H*_*2*_CH_3_, *Z* isomer), 2.50 (q, ^*3*^*J* = 7.4 Hz, 0.9H, C*H*_*2*_CH_3_, *E* isomer), 3.18 (t, ^*3*^*J* = 6.3 Hz, 0.9H, NHCH_2_CHCH_2_C*H*_*2*_NH, *E* isomer), 3.21 (t, ^*3*^*J* = 6.4 Hz, 1.1H, NHCH_2_CH_2_CH_2_C*H*_*2*_NH, *Z* isomer), 3.28 (t, ^*3*^*J* = 6.3 Hz, 0.9H, NHC*H*_*2*_CH_2_CH_2_CH_2_NH, *E* isomer), 3.33 (t, ^*3*^*J* = 6.2 Hz, 1.1H, NHC*H*_*2*_CH_2_CH_2_CH_2_NH, *Z* isomer), 4.45–4.49 (2 × t, 2H, 2 × NC*H*_*2*_), 6.37–6.46 (m, 1.4H, Ar*H* + CH=C*H*CONH), 6.60 (d, ^3^*J* = 15.8 Hz, 0.6H, CH=C*H*CONH, *Z* isomer), 6.64 (d, ^3^*J* = 8.5 Hz, 1.1H, Ar*H*, *Z* isomer), 6.78 (d, ^*3*^*J* = 8.3 Hz, 0.9H, Ar*H*, *E* isomer), 6.85 (d, ^*3*^*J* = 8.1 Hz, 0.9H, Ar*H*, *E* isomer), 7.02 (d, ^*3*^*J* = 8.3 Hz, 0.9H, Ar*H*, *E* isomer), 7.05–7.21 (m, 5.9H, Ar*H*), 7.22 (d, ^*3*^*J* = 8.0 Hz, 1.1H, Ar*H*, *Z* isomer), 7.24 (d, ^*3*^*J* = 8.3 Hz, 1H, Ar*H*), 7.27–7.31 (m, 1H, Ar*H*), 7.35 (d, ^*3*^*J* = 15.7 Hz, 0.4H, C*H*=CHCONH, *E* isomer), 7.50–7.57 (m, 1.7H, Ar*H* + C*H*=CHCONH), 7.64–7.70 (m, 1H, Ar*H*), 8.02 (d, ^*3*^*J* = 8.0 Hz, 1H, Ar*H*). ^13^C NMR: (176 MHz, CD_3_OD, *E*/*Z* = 45:55): δ 13.81, 13.88, 27.01, 27.55, 27.77, 27.87, 27.91, 28.72, 29.86, 29.94, 29.99, 30.11, 37.13, 39.98, 40.18, 40.24, 47.45, 115.27, 116.02, 116.12, 117.06, 121.11, 121.57, 125.49, 127.20, 127.36, 127.84, 128.66, 128.76, 128.92, 129.00, 130.84, 130.86, 131.10, 131.68, 132.44, 133.05, 133.56, 134.64, 135.29, 135.60, 136.53, 139.53, 139.68, 140.70, 141.39, 142.82, 143.63, 143.68, 143.87, 146.73, 146.98, 156.65, 157.55, 161.52, 168.72, 176.34, 177.30. HR-MS (*m*/*z*): calcd for C_45_H_50_N_4_O_4_S [M – H]^−^: 741.3553, found: 741.3558.

##### 4.1.3.2.1.2 *N*-[3-((*E*)-3-(4-((*E*/*Z*)-1-(4-Hydroxyphenyl)-2-phenylbut-1-enyl)phenyl)acrylamido)propyl]-4-[4-(4-(4-oxo-2-thioxodihydroquinazolin-3-yl)butanoyl)piperazin-1-yl]benzamide (**17**)

**17** was synthesized following the general procedure applying 68 mg of GW7604 (0.18 mmol) in 1 mL of dry DMF, 0.2 mL of DIPEA (1.13 mmol), and 104 mg of PyBOP (0.20 mmol) in 2 mL of dry DCM. **13** (1.1 equiv, 125 mg, 0.20 mmol) in 1 mL of dry DMF was added dropwise. Purification was carried out by flash column chromatography with a PE:EA:MeOH gradient (EA: 30 → 100% and then MeOH: 0 → 5%). **17** was obtained as a whitish-yellow powder (85 mg, 0.10 mmol, 54%). Purity: 99.8% (HPLC: 0.5 mM in MeOH, 254 nm). ^1^H NMR: (600 MHz, DMSO-*d*_6_, *E*/*Z* = 12:88): δ 0.86 (t, ^*3*^*J* = 7.4 Hz, 3H, CH_2_C*H*_*3*_), 1.64–1.67 (m, 0.2H, C*H*_*2*_, *E* isomer), 1.70 (p, ^*3*^*J* = 6.8 Hz, 1.8H, C*H*_*2*_, *Z* isomer), 1.98 (p, ^*3*^*J* = 7.0 Hz, 2H, C*H*_*2*_), 2.40 (q, ^*3*^*J* = 7.2 Hz, 2H, C*H*_*2*_CH_3_), 2.46 (t, ^*3*^*J* = 7.3 Hz, NCH_2_CH_2_C*H*_*2*_CON), 3.22–3.26 (m, 4H, NHC*H*_*2*_CH_2_C*H*_*2*_NH), 3.27–3.31 (m, 4H CON(C*H*_*2*_CH_2_)_2_N), 3.51–3.61 (m, 4H, CON(CH_2_C*H*_*2*_)_2_N), 4.46 (t, ^*3*^*J* = 7.1 Hz, NC*H*_*2*_), 6.42 (d, ^*3*^*J* = 8.5 Hz, 1.8H, Ar*H*, *Z* isomer), 6.46 (d, ^*3*^*J* = 15.8 Hz, 0.1H, CH=C*H*CONH, *E* isomer), 6.62–6.65 (m, 2.6H, Ar*H* + CH=C*H*CONH, *Z* isomers), 6.77 (d, ^*3*^*J* = 8.4 Hz, 0.2H, Ar*H*, *E* isomer), 6.84 (d, ^*3*^*J* = 8.2 Hz, 0.2H, Ar*H*, *E* isomer), 6.95–6.98 (m, 2.1H, Ar*H*), 7.01 (d, ^*3*^*J* = 8.3 Hz, 0.2H, Ar*H*, *E* isomer), 7.10–7.27 (m, 7H, Ar*H* + C*H*=CHCONH, *E* isomer), 7.31–7.33 (m, 1H, Ar*H*), 7.38 (d, ^*3*^*J* = 8.2 Hz, 1H, Ar*H*), 7.44 (d, ^*3*^*J* = 15.7 Hz, 0.9H, C*H*=CHCONH, *Z* isomer), 7.56 (d, ^*3*^*J* = 7.8 Hz, 1.8H, Ar*H*, *Z* isomer), 7.72–7.76 (m, 3H, Ar*H*), 7.94 (d, ^*3*^*J* = 7.8 Hz, 1H, Ar*H*), 8.06 (brt, ^*3*^*J* = 5.4 Hz, 0.1H, N*H*, *E* isomer), 8.16 (brt, ^*3*^*J* = 5.5 Hz, 0.9H, N*H*, *Z* isomer), 8.20 (brt, 0.1H, N*H*, *E* isomer), 8.24 (brt, ^*3*^*J* = 5.5 Hz, 0.9H, N*H*, *Z* isomer), 9.21 (s, 0.9H, O*H*, *Z* isomer), 9.46 (s, 0.1H, O*H*, *E* isomer), 12.90 (s, 1H, N*H*). ^13^C NMR: (151 MHz, DMSO-*d*_6_, *E*/*Z* = 12:88): δ 13.27, 22.13, 28.36, 29.38, 29.80, 36.56, 36.76, 40.54, 44.31, 45.19, 46.93, 47.21, 113.75, 114.33 *Z* isomer, 114.99 *E* isomer, 115.47, 121.88, 123.98, 124.02, 124.30, 126,03, 126.54 *E* isomer, 127.18, 127.34 *Z* isomer, 127.78 *Z* isomer, 127.85 *E* isomer, 128.30, 129.24, 129.49, 130.11 *E* isomer, 130.69 *E* isomer, 131.29 *Z* isomer, 132.90, 133.16, 135.26, 137.64, 138.09, 138.98, 140.59, 141.73, 144.52, 152.32, 155.31, 159.31, 164.94, 165.72, 169.89, 175.04. HR-MS (*m*/*z*): calcd for C_51_H_52_N_6_O_5_S [M – H]^−^: 859.3647, found: 859.3679.

##### 4.1.3.2.1.3 *N*-[3-((*E*)-3-(4-((*E*/*Z*)-1-(4-Hydroxyphenyl)-2-phenylbut-1-enyl)phenyl)acrylamido)propyl]-4-[4-(8-(4-oxo-2-thioxodihydroquinazolin-3-yl)octanoyl)piperazin-1-yl]benzamide (**18**)

**18** was synthesized according to the general procedure applying 68 mg of GW7604 (0.18 mmol) in 1 mL of dry DMF, 0.20 mL of DIPEA (1.13 mmol), and 104 mg of PyBOP (0.20 mmol) in 2 mL of dry DCM. **14** (1.1 equiv, 136 mg, 0.20 mmol) in 1 mL of dry DMF was added slowly. The crude product was purified by column chromatography with a DCM and MeOH gradient (96:4 → 95:5). **18** was obtained as a whitish-yellow powder (80 mg, 0.10 mmol, 48%). Purity: 99.7% (HPLC: 0.5 mM in MeOH, 254 nm). ^1^H NMR: (700 MHz, DMSO-*d*_6_, *E*/*Z* = 20:80): δ 0.86 (t, ^3^*J* = 7.3 Hz, 3H, CH_2_C*H*_*3*_), 1.30–1.36 (m, 6H, C*H*_*2*_), 1.52 (p, ^*3*^*J* = 6.7 Hz, 2H, C*H*_*2*_), 1.65–1.73 (m, 4H, C*H*_*2*_), 2.34 (t, ^*3*^*J* = 7.4 Hz, 2H, C*H*_*2*_CON(CH_2_CH_2_)_2_N), 2.39 (q, ^*3*^*J* = 7.2 Hz, 1.6H, C*H*_*2*_CH_3_, *Z* isomer), 2.44 (q, ^*3*^*J* = 7.4 Hz, 0.4H, C*H*_*2*_CH_3_, *E* isomer), 3.18–3.25 (m, 4H, NHC*H*_*2*_CH_2_C*H*_*2*_NH), 3.25–3.28 (m, 4H, CON(C*H*_*2*_CH_2_)_2_N), 3.56–3.62 (m, 4H, CON(CH_2_C*H*_*2*_)_2_N), 4.39 (t, ^*3*^*J* = 7.7 Hz, 2H, NC*H*_*2*_), 6.42 (d, ^*3*^*J* = 8.5 Hz, 1.6H, Ar*H*, *Z* isomer), 6.46 (d, ^*3*^*J* = 15.8 Hz, 0.2H, CH=C*H*CONH, *E* isomer), 6.59–6.66 (m, 2.4H, Ar*H*, *Z* isomer + CH=C*H*CONH, *Z* isomer), 6.77 (d, ^*3*^*J* = 8.3 Hz, 0.4H, Ar*H*, *E* isomer), 6.84 (d, ^*3*^*J* = 8.1 Hz, 0.4H, Ar*H*, *E* isomer), 6.94–6.99 (m, 2.2H, Ar*H*), 7.01 (d, ^*3*^*J* = 8.3 Hz, 0.4H, Ar*H*, *E* isomer), 7.04–7.29 (m, 7H, Ar*H* + C*H*=CHCONH, *E* isomer), 7.30–7.36 (m, 1H, Ar*H*), 7.39 (d, ^*3*^*J* = 8.2 Hz, 1H, Ar*H*), 7.44 (d, ^*3*^*J* = 15.7 Hz, 0.8H, C*H*=CHCONH, *Z* isomer), 7.56 (d, ^*3*^*J* = 8.0 Hz, 1.6H, Ar*H*, *Z* isomer), 7.72–7.76 (m, 3H, Ar*H*), 7.95 (d, ^*3*^*J* = 7.8 Hz, 1H, Ar*H*), 8.05 (brt, ^*3*^*J* = 5.5 Hz, 0.2H, N*H*, *E* isomer), 8.15 (brt, ^*3*^*J* = 5.4 Hz, 0.8H, N*H*, *Z* isomer), 8.20 (brt, ^*3*^*J* = 5.4 Hz, 0.2H, N*H*, *E* isomer), 8.23 (brt, ^*3*^*J* = 5.4 Hz, 0.8H, N*H*, *Z* isomer), 9.20 (s, 0.8H, O*H*, *Z* isomer), 9.45 (s, 0.2H, O*H*, *E* isomer), 12.91 (s, 1H, N*H*). ^13^C NMR: (176 MHz, DMSO-*d*_6_, *E*/*Z* = 20:80): δ 13.36, 24.68, 26.16, 26.26, 28.47, 28.63, 29.48, 32.22, 36.66, 36.87, 40.57, 44.46, 45.66, 47.14, 47.52, 113.86, 114.43 *Z* isomer, 115.09 *E* isomer, 115.48, 115.62, 121.99, 124.17, 124.43, 126.12, 126.63 *E* isomer, 127.24, 127.43 *Z* isomer, 127.87 *Z* isomer, 128.40, 129.33, 129.58, 130.20 *E* isomer, 130.78 *E* isomer, 131.38 *Z* isomer, 133.00, 133.26, 135.39, 137.75, 138.18, 139.09, 140.69, 141.83, 144.62, 152.40, 155.41, 159.21, 165.04, 165.81, 170.74, 174.98. HR-MS (*m*/*z*): calcd for C_55_H_60_N_6_O_5_S [M – H]^−^: 915.4273, found: 915.4283.

##### 4.1.3.2.2 Syntheses of the 5-Methoxybenzimidazoles **31**, **32**, **34**, **36**, **38**

##### 4.1.3.2.2.1 (*E*)-3-[4-((*E*/*Z*)-1-(4-Hydroxyphenyl)-2-phenylbut-1-enyl)phenyl]-*N*-[2-(3-(5-methoxy-1H-benzo[*d*]imidazol-2-yl)propanamido)ethyl]acrylamide (**31**)

**31** was synthesized according to the general procedure for heterodimer formation using 140 mg of GW7604 (0.38 mmol) in 2 mL of dry DMF, 0.4 mL of DIPEA (2.27 mmol), and 216 mg of PyBOP (0.42 mmol) in 2 mL of dry DCM. **26** (186 mg, 0.38 mmol) in 1 mL of dry DMF was added dropwise to this mixture. Column chromatography with DCM and MeOH (95:5 → 93:7) afforded **31** as orange crystals (116 mg, 0.19 mmol, 50%). Purity: 98.5% (HPLC: 0.5 mM in ACN + water (Na_2_SO_4_, 20 mM (pH 3)), 254 nm). ^1^H NMR: (700 MHz, CD_3_OD, *E*/*Z* = 55:45): δ 0.94 (t, ^*3*^*J* = 7.4 Hz, 3H, CH_2_C*H*_*3*_), 2.49 (q, ^*3*^*J* = 7.4 Hz, 0.9H, C*H*_*2*_CH_3_, *Z* isomer), 2.54 (q, ^*3*^*J =* 7.4 Hz, 1.1H, C*H*_*2*_CH_3_, *E* isomer), 2.73 (t, ^*3*^*J* = 7.5 Hz, 1.1H, C*H*_*2*_CH_2_CONH, *E* isomer), 2.76 (t, ^*3*^*J* = 7.5 Hz, 0.9H, C*H*_*2*_CH_2_CONH, *Z* isomer), 3.15 (t, ^*3*^*J* = 7.5 Hz, 1.1H, CH_2_C*H*_*2*_CONH, *E* isomer), 3.18 (t, ^*3*^*J* = 7.5 Hz, 0.9H, CH_2_C*H*_*2*_CONH, *Z* isomer), 3.34 (t, 1.1H, GW7604NHCH_2_C*H*_*2*_, *E* isomer), 3.36–3.41 (m, 2H, GW7604NHC*H*_2_C*H*_2_), 3.43 (t, ^*3*^*J* = 5.8 Hz, 0.9H, GW7604NHC*H*_*2*_CH_2_, *Z* isomer), 3.79 (s, 1.6H, OC*H*_*3*_, *E* isomer), 3.80 (s, 1.4H, OC*H*_*3*_, *Z* isomer), 6.36 (d, ^*3*^*J* = 15.7 Hz, 0.5H, CH=C*H*CONH, *E* isomer), 6.44 (d, ^*3*^*J* = 8.6 Hz, 0.9H, Ar*H*, *Z* isomer), 6.53 (d, ^*3*^*J* = 15.8 Hz, 0.5H, CH=C*H*CONH, *Z* isomer), 6.68 (d, ^*3*^*J* = 8.6 Hz, 0.9H, Ar*H*, *Z* isomer), 6.81 (d, ^*3*^*J* = 8.4 Hz, 1.1H, Ar*H*, *E* isomer), 6.82–6.86 (m, 1H, Ar*H*), 6.89 (d, ^*3*^*J* = 8.3 Hz, 1.1H, Ar*H*, *E* isomer), 6.98–7.04 (m, 1H, Ar*H*), 7.06 (d, ^*3*^*J* = 8.5 Hz, 1.1H, Ar*H*, *E* isomer), 7.08–7.23 (m, 6.1H, Ar*H*), 7.25 (d, ^*3*^*J* = 8.1 Hz, 0.9H, Ar*H*, *Z* isomer), 7.30–7.43 (m, 1.6H, Ar*H* + C*H*=CHCONH, *E* isomer), 7.51 (d, ^*3*^*J* = 8.1 Hz, 0.9H, Ar*H*, *Z* isomer), 7.54 (d, ^*3*^*J* = 15.8 Hz, 0.5H, C*H*=CHCONH, *Z* isomer). ^13^C NMR: (700 MHz, CD_3_OD, *E*/*Z* = 55:45): δ 13.80, 13.86, 25.50, 29.85, 29.99, 34.64, 40.16, 40.20, 40.26, 56.19, 98.08, 112.87, 115.28, 116.03, 116.21, 120.96, 121.43, 127.22, 137.37, 127.87, 128.68, 128.93, 129.00, 130.83, 130.86, 131.09, 131.67, 132.42, 133.04, 133.48, 134.55, 135.29, 135.59, 139.54, 139.68, 141.57, 141.59, 142.86, 143.64, 143.67, 143.92, 146.82, 147.06, 155.04, 155.06, 156.67, 157.56, 157.90, 169.04, 174.52, 174.56. HR-MS (*m*/*z*): calcd for C_38_H_38_N_4_O_4_ [M + H]^+^: 615.2966, found: 615.2964.

##### 4.1.3.2.2.2 (*E*)-3-[4-((*E*/*Z*)-1-(4-Hydroxyphenyl)-2-phenylbut-1-enyl)phenyl]-*N*-[3-(3-(5-methoxy-1H-benzo[*d*]imidazol-2-yl)propanamido)propyl]acrylamide (**32**)

**32** was synthesized according to the general procedure for heterodimer formation using 44 mg of GW7604 (0.12 mmol) in 1.3 mL of dry DMF, 0.13 mL of DIPEA (0.71 mmol), and 68 mg of PyBOP (0.13 mmol) in 1 mL of dry DCM. **27** (60 mg, 0.12 mmol) in 1 mL of dry DMF was added dropwise. Purification by column chromatography with a gradient using DCM and MeOH (95:5 → 90:10) led to **32** as a light-yellow powder (44 mg, 0.07 mmol, 60%). Purity: 98.7% (HPLC: 1.0 mM in ACN + water (Na_2_SO_4_, 20 mM (pH 3)), 254 nm). ^1^H NMR: (700 MHz, CD_3_OD, *E*/*Z* = 55:45): δ 0.92 (t, ^*3*^*J* = 7.4 Hz, 3H, CH_2_C*H*_*3*_), 1.66 (p, ^*3*^*J* = 6.8 Hz, 1.1H, NHCH_2_C*H*_*2*_CH_2_NH, *E* isomer), 1.71 (p, ^*3*^*J* = 6.8 Hz, 0.9H, NHCH_2_C*H*_*2*_CH_2_NH, *Z* isomer), 2.48 (q, ^*3*^*J* = 7.3 Hz, 0.9H, C*H*_*2*_CH_3_, *Z* isomer), 2.52 (q, ^*3*^*J =* 7.4 Hz, 1.1H, C*H*_*2*_CH_3_, *E* isomer), 2.69–2.78 (2 × t, ^*3*^*J* = 7.5 Hz, 2H, 2 × C*H*_*2*_CH_2_CONH), 3.14–3.19 (2 × t, ^*3*^*J* = 7.5 Hz, 2H, 2 × CH_2_C*H*_*2*_CONH), 3.19–3.23 (m, 2H, GW7604NHCH_2_CH_2_C*H*_*2*_NH), 3.25 (t, ^*3*^*J* = 6.7 Hz, 1.1H, GW7604NHC*H*_*2*_, *E* isomer), 3.27 (t, ^*3*^*J* = 6.7 Hz, 0.9H, GW7604NHC*H*_*2*_, *Z* isomer), 3.79 (s, 1.6H, OC*H*_*3*_, *E* isomer), 3.80 (s, 1.4H, OC*H*_*3*_, *Z* isomer), 6.38–6.45 (m, 1.5H, Ar*H*, *Z* isomer + CH=C*H*CONH, *E* isomer), 6.58 (d, ^*3*^*J* = 15.8 Hz, 0.5H, CH=C*H*CONH, *Z* isomer), 6.66 (d, ^*3*^*J* = 8.4 Hz, 0.9H, Ar*H*, *Z* isomer), 6.78 (d, ^*3*^*J* = 8.3 Hz, 1.1H, Ar*H*, *E* isomer), 6.81–6.86 (m, 1H, Ar*H*), 6.87 (d, ^*3*^*J* = 8.1 Hz, 1.1H, Ar*H*, *E* isomer), 6.98–7.02 (m, 1H, Ar*H*), 7.04 (d, ^*3*^*J* = 8.3 Hz, 1.1H, Ar*H*, *E* isomer), 7.09–7.19 (m, 6.1H, Ar*H*), 7.24 (d, ^*3*^*J* = 7.9 Hz, 0.9H, Ar*H*, *Z* isomer), 7.32–7.40 (m, 1.6H, Ar*H* + C*H*=CHCONH, *E* isomer), 7.51–7.57 (m, 1.4H, Ar*H* + C*H*=CHCONH, *Z* isomer). ^13^C NMR: (700 MHz, CD_3_OD, *E*/*Z* = 55:45): δ 13.81 *E* isomer, 13.86 *Z* isomer, 25.54 *E* isomer, 29.85 *Z* isomer, 29.99 *E* isomer, 30.16 *E* isomer, 30.20 *Z* isomer, 34.71, 37.81, 37.86 *E* isomer, 37.89 *Z* isomer, 56.21, 97.96, 113.10, 115.28 *Z* isomer, 116.03 *E* isomer, 116.21, 121.04 *E* isomer, 121.50 *Z* isomer, 127.22 *Z* isomer, 127.37 *E* isomer, 127.85 *E* isomer, 128.67 *Z* isomer, 128.93 *Z* isomer, 129.00 *E* isomer, 130.84 *Z* isomer, 130.86 *E* isomer, 131.11 *Z* isomer, 131.67 *E* isomer, 132.44 *E* isomer, 133.04 *Z* isomer, 133.54 *E* isomer, 134.62 *Z* isomer, 135.29 *Z* isomer, 135.60 *E* isomer, 139.54 *Z* isomer, 139.68 *E* isomer, 141.47, 142.86 *Z* isomer, 143.65 *E* isomer, 143.67 *Z* isomer, 143.92 *E* isomer, 146.80 *E* isomer, 147.05 *Z* isomer, 154.95 *E* isomer, 154.97 *Z* isomer, 156.67 *Z* isomer, 157.57 *E* isomer, 158.03, 168.80, 174.05 *Z* isomer, 174.08 *E* isomer. HR-MS (*m*/*z*): calcd for C_39_H_40_N_4_O_4_ [M + H]^+^: 629.3122, found: 629.3115.

##### 4.1.3.2.2.3 (*E*)-3-[4-((*E*/*Z*)-1-(4-Hydroxyphenyl)-2-phenylbut-1-enyl)phenyl]-*N*-[4-(3-(5-methoxy-1*H*-benzo[*d*]imidazol-2-yl)propanamido)butyl]acrylamide (**34**)

**34** was synthesized according to the general procedure using 50 mg of GW7604 (0.14 mmol) in 1.3 mL of dry DMF, 0.14 mL of DIPEA (0.81 mmol), and 84 mg of PyBOP (1.2 equiv, 0.13 mmol) in 1 mL of dry DCM. **28** (70 mg, 0.14 mmol) in 1 mL of dry DMF was added dropwise. Purification by column chromatography with DCM and MeOH (95:5) and subsequent crystallization from MeOH/water provided **34** as a peach-colored powder (43 mg, 0.07 mmol, 51.8%). Purity: 96.4% (HPLC: 1.0 mM in ACN + water (Na_2_SO_4_, 20 mM (pH 3)), 254 nm). ^1^H NMR: (600 MHz, CD_3_OD, *E*/*Z* = 55:45): δ 0.92 (t, ^*3*^*J* = 7.3 Hz, 3H, CH_2_C*H*_*3*_), 1.44–1.49 (m, 2H, C*H*_*2*_), 1.49–1.55 (m, 2H, C*H*_*2*_), 2.47 (q, ^*3*^*J* = 7.4 Hz, 0.9H, C*H*_*2*_CH_3_, *Z* isomer), 2.52 (q, ^*3*^*J =* 7.4 Hz, 1.1H, C*H*_*2*_CH_3_, *E* isomer), 2.63–2.76 (2 × t, 2H, 2 × C*H*_*2*_CH_2_CONH), 3.10–3.16 (2 × t, ^*3*^*J* = 7.5 Hz, 2H, 2 × CH_2_C*H*_*2*_CONH), 3.18 (t, ^*3*^*J* = 6.2 Hz, 1.1H, GW7604NHCH_2_CH_2_CH_2_C*H*_*2*_NH, *E* isomer) 3.19–3.23 (m, 2H, C*H*_*2*_), 3.26 (t, ^*3*^*J* = 6.3 Hz, 0.9H GW7604NHC*H*_*2*_, *Z* isomer), 3.78 (s, 1.6H, OC*H*_*3*_, *E* isomer), 3.80 (s, 1.4H, OC*H*_*3*_, *Z* isomer), 6.38–6.45 (m, 1.5H, Ar*H* + CH=C*H*CONH), 6.58 (d, ^*3*^*J* = 15.8 Hz, 0.5H, CH=C*H*CONH, *Z* isomer), 6.66 (d, ^*3*^*J* = 8.7 Hz, 0.9H, Ar*H*, *Z* isomer), 6.78 (d, ^*3*^*J* = 8.6 Hz, 1.1H, Ar*H*, *E* isomer), 6.80–6.84 (2 × dd, 1H, Ar*H*), 6.87 (d, ^*3*^*J* = 8.3 Hz, 1.1H, Ar*H*, *E* isomer), 6.97–7.02 (m, 1H, Ar*H*), 7.04 (d, ^*3*^*J* = 8.6 Hz, 1.1H, Ar*H*, *E* isomer), 7.07–7.20 (m, 6.1H, Ar*H*), 7.24 (d, ^*3*^*J* = 8.2 Hz, 0.9H, Ar*H*, *Z* isomer), 7.31–7.39 (m, 1.6H, Ar*H* + CH=C*H*CONH), 7.50–7.57 (m, 1.4H, Ar*H* + CH=C*H*CONH). ^13^C NMR: (151 MHz, CD_3_OD, *E*/*Z* = 55:45): δ 13.81, 13.87, 25.77, 27.68, 27.72, 27.75, 27.80, 29.85, 29.99, 34.86, 40.00, 40.03, 40.13, 40.19, 56.20, 98.04, 112.75, 115.29, 116.04, 116.41, 121.11, 121.58, 127.22, 127.36, 127.83, 128.66, 128.93, 129.00, 129.87, 130.84, 130.86, 131.11, 131.67, 132.44, 133.04, 133.58, 134.66, 135.30, 135.61, 139.54, 139.69, 141.37, 142.85, 143.65, 143.68, 143.90, 146.76, 147.01, 155.04, 156.67, 157.56, 157.83, 168.70, 168.72, 174.05, 174.07. HR-MS (*m*/*z*): calcd for C_40_H_42_N_4_O_4_ [M + H]^+^: 643.3279, found: 643.3271.

##### 4.1.3.2.2.4 (*E*)-3-[4-((*E*/*Z*)-1-(4-Hydroxyphenyl)-2-phenylbut-1-enyl)phenyl]-N-[5-(3-(5-methoxy-1H-benzo[*d*]imidazol-2-yl)propanamido)pentyl]acrylamide (**36**)

**36** was synthesized according to the general procedure for heterodimer formation using 77 mg of GW7604 (0.21 mmol) in 2 mL of dry DMF, 0.22 mL of DIPEA (1.24 mmol), and 118 mg of PyBOP (0.23 mmol) in 1.5 mL of dry DCM. **29** (110 mg, 0.21 mmol) in 1 mL of dry DMF was added dropwise. Purification by column chromatography with a DCM and MeOH gradient (95:5 → 93:7) led to **36** as orange, sparkling crystals (70 mg, 0.11 mmol, 52%). Purity: 98.5% (HPLC: 1.0 mM in ACN + water (Na_2_SO_4_, 20 mM (pH 3)), 254 nm and 281 nm). ^1^H NMR: (500 MHz, CD_3_OD, *E*/*Z* = 50:50): δ 0.83 – 0.99 (2 × t, 3H, 2 × CH_2_C*H*_*3*_), 1.26–1.37 (m, 2H, NHCH_2_CH_2_C*H*_*2*_CH_2_CH_2_NH), 1.45–1.55 (m, 4H, NHCH_2_C*H*_*2*_CH_2_C*H*_*2*_CH_2_NH), 2.44–2.48 (m, 1H, C*H*_*2*_CH_3_, *Z* isomer), 2.52 (d, ^*3*^*J* = 7.4 Hz, 1H, C*H*_*2*_CH_3_, *E* isomer), 2.66–2.75 (2 × t, 2H, 2 × C*H*_*2*_CH_2_CONH), 3.10–3.23 (m, 5H, C*H*_*2*_), 3.26 (t, ^*3*^*J* = 7.0 Hz, 1H, GW7604NHC*H*_*2*_, *Z* isomer), 3.74–3.88 (2 × s, 3H, 2 × OC*H*_*3*_), 6.37–6.47 (m, 1.5H, Ar*H* + CH=C*H*CONH), 6.60 (d, ^*3*^*J* = 15.8 Hz, 0.5H, CH=C*H*CONH, *Z* isomer), 6.62–6.71 (m, 1H, Ar*H*, *Z* isomer), 6.79 (d, ^*3*^*J* = 8.5 Hz, 1H, Ar*H*, *E* isomer), 6.82–6.89 (m, 2H, Ar*H*), 6.97–7.02 (m, 1H, Ar*H*), 7.03 (d, ^*3*^*J* = 8.4 Hz, 1H, Ar*H*, *E* isomer), 7.08–7.18 (m, 6H, Ar*H*), 7.20–7.25 (m, 1H, Ar*H*), 7.32–7.40 (m, 1.5H, Ar*H* + C*H*=CHCONH), 7.49–7.57 (m, 1.5H, Ar*H* + C*H*=CHCONH). ^13^C NMR: (126 MHz, CD_3_OD, *E*/*Z* = 50:50): δ 13.81, 13.86, 25.10, 25.13, 25.75, 25.78, 29.85, 29.93, 29.95, 30.01, 30.05, 34.85, 34.88, 40.22, 40.30, 40.36, 56.21, 98.06, 112.87, 115.27, 116.02, 116.18, 121.12, 121.58, 127.21, 127.35, 127.80, 128.62, 128.92, 128.98, 130.83, 130.85, 131.09, 131.67, 132.43, 133.04, 133.55, 134.63, 135.28, 135.60, 139.53, 139.67, 141.33, 142.82, 143.63, 143.67, 143.88, 146.72, 146.97, 154.99, 156.66, 157.55, 157.90, 168.70, 173.96. HR-MS (*m*/*z*): calcd for C_41_H_44_N_4_O_4_ [M + H]^+^: 657.3435, found: 657.3462.

##### 4.1.3.2.2.5 (*E*)-3-[4-((*E*/*Z*)-1-(4-Hydroxyphenyl)-2-phenylbut-1-enyl)phenyl]-*N*-[6-(3-(5-methoxy-1*H*-benzo[*d*]imidazol-2-yl)propanamido)hexyl]acrylamide (**38**)

**38** was synthesized according to the general procedure for heterodimer formation using 139 mg of GW7604 (0.38 mmol) in 2 mL of dry DMF, 0.39 mL of DIPEA (2.26 mmol), and 215 mg of PyBOP (0.41 mmol) in 2 mL of dry DCM. **30** (200 mg, 0.38 mmol) in 1 mL of dry DMF was added dropwise. Purification by column chromatography with a gradient of DCM and MeOH (96:4 → 95:5) afforded **38** as a light-yellow powder (139 mg, 0.21 mmol, 55%). Purity: 99.0% (HPLC: 1.0 mM in ACN + water (Na_2_SO_4_, 20 mM (pH 3)), 254 nm). ^1^H NMR: (500 MHz, CD_3_OD, *E*/*Z* = 50:50): δ 0.93 (t, ^*3*^*J* = 7.4 Hz, 3H, CH_2_C*H*_*3*_), 1.20–1.39 (m, 4H, C*H*_*2*_), 1.39–1.56 (m, 4H, C*H*_*2*_), 2.48 (q, ^*3*^*J* = 7.3 Hz, 1H, C*H*_*2*_CH_3_, *Z* isomer), 2.52 (q, ^*3*^*J* = 7.5 Hz, 1H, C*H*_*2*_CH_3_, *E* isomer), 2.71 (2 × t, 2H, 2 × C*H*_*2*_CH_2_CONH), 3.09–3.19 (m, 4H, C*H*_*2*_NH + CH_2_C*H*_*2*_CONH), 3.22 (t, ^*3*^*J* = 7.0 Hz, 1H, GW7604NHC*H*_*2*_, *E* isomer), 3.27 (t, ^*3*^*J* = 7.1 Hz, 1H, GW7604NHC*H*_*2*_, *Z* isomer), 3.81–3.85 (2 × s, 3H, 2 × OC*H*_*3*_), 6.41–6.45 (m, 1.5H, Ar*H* + CH=C*H*CONH), 6.61 (d, ^*3*^*J* = 15.8 Hz, 0.5H, CH=C*H*CONH, *Z* isomer), 6.67 (d, ^*3*^*J* = 8.7 Hz, 1H, Ar*H*, *Z* isomer), 6.79 (d, ^*3*^*J* = 8.6 Hz, 1H, Ar*H*, *E* isomer), 6.82–6.85 (2 × dd, 1H, Ar*H*), 6.88 (d, ^3^*J* = 8.2 Hz, 1H, Ar*H*, *E* isomer), 6.98–7.02 (m, 1H, Ar*H*), 7.04 (d, ^*3*^*J* = 8.6 Hz, 1H, Ar*H*, *E* isomer), 7.07–7.20 (m, 6H, Ar*H*), 7.25 (d, ^*3*^*J* = 8.1 Hz, 1H, Ar*H*, *Z* isomer), 7.33–7.38 (m, 1.5H, Ar*H* + C*H*=CHCONH), 7.52–7.55 (m, 1.5H, Ar*H* + C*H*=CHCONH). ^13^C NMR: (126 MHz, CD_3_OD, *E*/*Z* = 50:50): δ 13.81, 13.87, 25.78, 27.43, 27.46, 27.57, 27.60, 29.86, 30.00, 30.26, 30.31, 34.86, 40.24, 40.39, 40.45, 56.21, 98.02, 112.79, 115.27, 116.02, 121.16, 121.62, 127.21, 127.35, 127.81, 128.63, 128.92, 128.99, 130.83, 130.85, 131.09, 131.66, 132.43, 133.03, 133.59, 134.67, 135.28, 135.60, 139.54, 139.68, 141.29, 142.83, 143.63, 143.66, 143.88, 146.72, 146.97, 154.99, 156.66, 157.55, 157.86, 168.65, 173.94. HR-MS (*m*/*z*): calcd for C_42_H_46_N_4_O_4_ [M + H]^+^: 671.3592, found: 671.3588.

##### 4.1.3.2.3 Syntheses of the 5-Hydroxybenzimidazoles **39**–**43**

General Procedure for Methyl Ether Cleavage:

The respective methoxy-protected GW7604-benzimidazole derivative (1 equiv) was dissolved in dry DCM or dry chlorobenzene. The suspension was cooled to −78 °C, and a BBr_3_ solution in DCM (1 M, 3.5 equiv for one methoxy group, 6–7 equiv for two methoxy groups) was added dropwise. The solution was further stirred at 0–25 °C for 3–24 h. MeOH (10 mL) was added to stop the reaction, stirred for 30 min, and concentrated under reduced pressure. The residue was neutralized with 6 N NaOH (pH = 8–9), and EA was added. The aqueous phase was extracted three times, and the combined organic phases were dried over anhydrous Na_2_SO_4_, evaporated to dryness, and further purified by column chromatography.

##### 4.1.3.2.3.1 (*E*)-*N*-[2-(3-(5-Hydroxy-1*H*-benzo[*d*]imidazol-2-yl)propanamido)ethyl]-3-[4-((*E*/*Z*)-1-(4-hydroxyphenyl)-2-phenylbut-1-enyl)phenyl]acrylamide (**39**)

**39** was synthesized according to the general procedure applying 93 mg of **31** (0.15 mmol) in 5 mL of dry chlorobenzene and 0.52 mL of a BBr_3_ solution (0.52 mmol) at ambient temperature. The reaction time was 24 h. Purification was performed by column chromatography with DCM and MeOH (9:1), affording **39** as a white-yellowish powder (30 mg, 0.05 mmol, 42%). Purity: 95.0% (HPLC: 1.0 mM in MeOH, 254 nm). ^1^H NMR: (500 MHz, CD_3_OD, *E*/*Z* = 50:50): δ 0.92 (t, ^*3*^*J* = 7.2 Hz, 3H, CH_2_C*H*_*3*_), 2.47 (q, ^*3*^*J* = 7.3 Hz, 1H, C*H*_*2*_CH_3_, *Z* isomer), 2.51 (q, ^*3*^*J =* 7.3 Hz, 1H, C*H*_*2*_CH_3_, *E* isomer), 2.65–2.76 (2 × t, 2H, 2 × C*H*_*2*_CH_2_CONH), 3.07–3.16 (2 × t, 2H, 2 × CH_2_C*H*_*2*_CONH), 3.33–3.46 (m, 4H, NHC*H*_*2*_C*H*_*2*_NH), 6.37 (d, ^*3*^*J* = 15.7 Hz, 0.5H, CH=C*H*CONH, *E* isomer), 6.42 (d, ^*3*^*J* = 8.0 Hz, 1H, Ar*H*, *Z* isomer), 6.53 (d, ^3^*J* = 15.7 Hz, 0.5H, CH=C*H*CONH, *Z* isomer), 6.66 (d, ^*3*^*J* = 8.1 Hz, 1H, Ar*H*, *Z* isomer), 6.69–6.75 (m, 1H, Ar*H*), 6.78 (d, ^*3*^*J* = 7.9 Hz, 1H, Ar*H*, *E* isomer), 6.82–6.92 (m, 2H, Ar*H*), 7.04 (d, ^*3*^*J* = 8.1 Hz, 1H, Ar*H*, *E* isomer), 7.05–7.22 (m, 6H, Ar*H*), 7.24 (d, ^*3*^*J* = 7.7 Hz, 1H, Ar*H*, *Z* isomer), 7.26–7.32 (m, 1H, Ar*H*), 7.34 (d, ^*3*^*J* = 15.7 Hz, 0.5H, C*H*=CHCONH, *E* isomer), 7.46–7.57 (m, 1.5H, Ar*H* + C*H*=CHCONH). ^13^C NMR: (126 MHz, CD_3_OD, *E*/*Z* = 50:50): δ 13.85, 13.90, 25.54, 29.85, 29.99, 34.70, 40.16, 99.98, 112.77, 115.26, 116.01, 116.22, 120.88, 121.35, 127.19, 127.33, 127.85, 128.67, 128.92, 128.97, 130.81, 131.08, 131.66, 132.42, 133.04, 133.40, 133.95, 134.47, 135.24, 135.54, 139.45, 139.60, 141.61, 142.79, 143.56, 143.60, 143.85, 146.77, 147.01, 154.68, 156.60, 157.49, 168.98, 169.00, 174.49, 174.53. HR-MS (*m*/*z*): calcd for C_37_H_36_N_4_O_4_ [M + H]^+^: 601.2809, found: 601.2809.

##### 4.1.3.2.3.2 (*E*)-*N*-[3-(3-(5-Hydroxy-1*H*-benzo[*d*]imidazol-2-yl)propanamido)propyl]-3-[4-((*E*/*Z*)-1-(4-hydroxyphenyl)-2-phenylbut-1-enyl)phenyl]acrylamide (**40**)

Synthesis of **40** was carried out according to the general procedure applying 68 mg of **33** (0.11 mmol) dissolved in 3.6 mL of dry DCM and 0.71 mL of BBr_3_ solution (0.71 mmol) at 0 °C. The reaction time was 2 h. Purification was performed by column chromatography with DCM and MeOH (9:1), affording **40** as a white-yellowish powder (58 mg, 0.09 mmol, 81%). Purity: 99.7% (HPLC: 1.0 mM in ACN + water (Na_2_SO_4_, 20 mM (pH 3)), 254 nm). ^1^H NMR: (700 MHz, CD_3_OD, *E*/*Z* = 50:50): δ 0.92 (t, ^*3*^*J* = 7.4 Hz, 3H, CH_2_C*H*_*3*_), 1.66 (p, ^*3*^*J =* 6.8 Hz, 1H, NHCH_2_C*H*_*2*_CH_2_NH, *E* isomer), 1.71 (p, ^*3*^*J =* 6.8 Hz, 1H, NHCH_2_C*H*_*2*_CH_2_NH, *Z* isomer), 2.47 (q, ^*3*^*J* = 7.4 Hz, 1H, C*H*_*2*_CH_3_, *Z* isomer), 2.52 (q, ^*3*^*J =* 7.4 Hz, 1H, C*H*_*2*_CH_3_, *E* isomer), 2.68–2.74 (2 × t, 2H, 2 × C*H*_*2*_CH_2_CONH), 3.11 (t, ^*3*^*J* = 7.5 Hz, 1H, CH_2_C*H*_*2*_CONH, *E* isomer), 3.13 (t, ^*3*^*J* = 7.5 Hz, 1H, CH_2_C*H*_*2*_CONH, *Z* isomer), 3.19–3.23 (2 × t, 2H, 2 × GW7604NHCH_2_CH_2_C*H*_*2*_NH), 3.24 (t, ^*3*^*J* = 6.9 Hz, 1H, GW7604NHC*H*_*2*_, *E* isomer), 3.27 (t, ^*3*^*J* = 6.9 Hz, 1H, GW7604NHC*H*_*2*_, *Z* isomer), 6.40–6.43 (m, 1.5H, Ar*H* + CH=C*H*CONH), 6.59 (d, ^*3*^*J* = 15.8 Hz, 0.5H, CH=C*H*CONH, *Z* isomer), 6.66 (d, ^*3*^*J* = 8.7 Hz, 1H, Ar*H*, *Z* isomer), 6.69–6.74 (2 × dd, 1H, Ar*H*), 6.78 (d, ^*3*^*J* = 8.5 Hz, 1H, Ar*H*, *E* isomer), 6.83–6.91 (m, 2H, Ar*H*), 7.04 (d, ^*3*^*J* = 8.5 Hz, 1.1H, Ar*H*, *E* isomer), 7.07–7.21 (m, 6H, Ar*H*), 7.24 (d, ^*3*^*J* = 8.1 Hz, 1H, Ar*H*, *Z* isomer), 7.27–7.32 (m, 1H, Ar*H*), 7.35 (d, ^*3*^*J* = 15.7 Hz, 0.5H, C*H*=CHCONH, *E* isomer), 7.52–7.58 (m, 1.5H, Ar*H* + C*H*=CHCONH). ^13^C NMR: (176 MHz, CD_3_OD, *E*/*Z* = 50:50): δ 13.81, 13.86, 25.72, 29.85, 30.00, 30.18, 30.22, 34.91, 37.83, 37.85, 37.87, 37.92, 112.71, 115.29, 116.04, 121.06, 121.52, 127.22, 127.37, 127.86, 128.68, 128.93, 129.00, 130.85, 130.87, 131.11, 131.68, 132.45, 133.05, 133.56, 134.64, 135.30, 135.61, 139.55, 139.70, 141.48, 142.86, 143.65, 143.69, 143.91, 146.79, 147.04, 154.67, 154.75, 156.68, 157.57, 168.83, 174.26. HR-MS (*m*/*z*): calcd for C_38_H_38_N_4_O_4_ [M + H]^+^: 615.2966, found: 615.2960.

##### 4.1.3.2.3.3 (*E*)-*N*-[4-(3-(5-Hydroxy-1*H*-benzo[*d*]imidazol-2-yl)propanamido)butyl]-3-[4-((*E*/*Z*)-1-(4-hydroxyphenyl)-2-phenylbut-1-enyl)phenyl]acrylamide (**41**)

**41** was synthesized according to the general procedure with 112 mg of **35** (0.17 mmol) dissolved in 5.5 mL of dry DCM and 1.02 mL of BBr_3_ solution (1.02 mmol) at 0 °C. The reaction time was 4 h. Purification was achieved by column chromatography with DCM and MeOH (9:1), affording **41** as a white-yellowish powder (48 mg, 0.076 mmol, 45%). Purity: 98.3% (HPLC: 1.0 mM in ACN + water (Na_2_SO_4_, 20 mM (pH 3)), 283 nm). ^1^H NMR: (600 MHz, CD_3_OD, *E*/*Z* = 50:50): δ 0.92 (t, ^*3*^*J* = 7.4 Hz, 3H, CH_2_C*H*_*3*_), 1.44–1.50 (m, 2H, C*H*_*2*_), 1.44–1.50 (m, 2H, C*H*_*2*_), 1.50–1.53 (m, 2H, C*H*_*2*_), 2.47 (q, ^*3*^*J* = 7.4 Hz, 1H, C*H*_*2*_CH_3_, *Z* isomer), 2.51 (q, ^*3*^*J =* 7.4 Hz, 1H, C*H*_*2*_CH_3_, *E* isomer), 2.68 (t, ^*3*^*J* = 7.6 Hz, 1H, C*H*_*2*_CH_2_CONH), 2.70 (t, ^*3*^*J* = 7.6 Hz, 1H, C*H*_*2*_CH_2_CONH), 3.08–3.14 (2 × t, 2H, 2 × CH_2_C*H*_*2*_CONH), 3.17 (t, ^*3*^*J =* 6.4 Hz, 1H, GW7604NHCH_2_CH_2_CH_2_C*H*_*2*_NH, *E* isomer), 3.19–3.23 (m, 2H, C*H*_*2*_), 3.26 (t, ^*3*^*J =* 6.4 Hz, 1H, GW7604NHC*H*_*2*_, *Z* isomer), 6.37–6.46 (m, 1.5H, Ar*H* + CH=C*H*CONH), 6.59 (d, ^*3*^*J* = 15.8 Hz, 0.5H, CH=C*H*CONH, *Z* isomer), 6.66 (d, ^*3*^*J* = 8.8 Hz, 1H, Ar*H*, *Z* isomer), 6.70–6.76 (2 × dd, 1H, Ar*H*), 6.78 (d, ^*3*^*J* = 8.6 Hz, 1H, Ar*H*, *E* isomer), 6.84–6.91 (m, 2H, Ar*H*), 7.03 (d, ^*3*^*J* = 8.6 Hz, 1H, Ar*H*, *E* isomer), 7.06–7.21 (m, 6H, Ar*H*), 7.24 (d, ^*3*^*J* = 8.2 Hz, 1H, Ar*H*, *Z* isomer), 7.26–7.32 (2 × d, 1H, Ar*H*), 7.35 (d, ^*3*^*J* = 15.7 Hz, 0.5H, C*H*=CHCONH, *E* isomer), 7.52–7.57 (m, 1.5H, Ar*H* + C*H*=CHCONH). ^13^C NMR: (151 MHz, CD_3_OD, *E*/*Z* = 50:50): δ 13.81, 13.87, 25.70, 25.71, 27.69, 27.73, 27.78, 29.85, 29.99, 34.84, 34.85, 40.00, 40.02, 40.11, 40.17, 99.97, 112.83, 115.27, 116.02, 116.23, 121.08, 121.54, 127.21, 127.36, 127.84, 128.66, 128.93, 129.00, 130.83, 130.86, 131.10, 131.67, 132.44, 133.04, 133.56, 134.64, 135.28, 135.59, 139.53, 139.53, 139.67, 141.40, 142.84, 143.63, 143.67, 143.89, 146.76, 147.01, 154.69, 154.76, 156.66, 157.56, 168.71, 168.73, 174.03, 174.05. HR–zMS (*m*/*z*): calcd for C_39_H_40_N_4_O_4_ [M + H]^+^: 629.3050, found: 629.3098.

##### 4.1.3.2.3.4 (*E*)-*N*-[5-(3-(5-Hydroxy-1*H*-benzo[*d*]imidazol-2-yl)propanamido)pentyl]-3-[4-((*E*/*Z*)-1-(4-hydroxyphenyl)-2-phenylbut-1-enyl)phenyl]acrylamide (**42**)

**42** was synthesized according to the general procedure with 61 mg of **37** (0.091 mmol) dissolved in 3.5 mL of dry DCM and 0.64 mL of BBr_3_ solution (0.64 mmol) at 0 °C. The reaction time was 4.5 h. Purification was achieved by column chromatography with DCM and MeOH (9:1) followed by crystallization from MeOH/water. **42** was obtained as a white powder (12 mg, 0.019 mmol, 21%). Purity: 98.6% (HPLC: 1.0 mM in ACN + water (Na_2_SO_4_, 20 mM (pH 3)), 283 nm). ^1^H NMR: (500 MHz, CD_3_OD, *E*/*Z* = 50:50): δ 0.83–0.99 (2 × t, ^*3*^*J* = 7.4 Hz, 3H, 2 × CH_2_C*H*_*3*_), 1.23–1.36 (m, 2H, NHCH_2_CH_2_C*H*_*2*_CH_2_CH_2_NH), 1.40–1.59 (m, 4H, NHCH_2_C*H*_*2*_CH_2_C*H*_*2*_CH_2_NH), 2.46 (q, ^*3*^*J* = 7.4 Hz, 1H, C*H*_*2*_CH_3_, *Z* isomer), 2.51 (q, ^*3*^*J* = 7.4 Hz, 1H, C*H*_*2*_CH_3_, *E* isomer), 2.62–2.79 (2 × t, 2H, 2 × C*H*_*2*_CH_2_CONH), 3.07–3.19 (m, 4H, CH_2_C*H*_*2*_CONH + GW7604NHCH_2_CH_2_CH_2_CH_2_C*H*_*2*_NH), 3.21 (t, ^*3*^*J* = 7.1 Hz, 1H, GW7604NHC*H*_*2*_, *E* isomer), 3.26 (t, ^*3*^*J* = 7.0 Hz, GW7604NHC*H*_*2*_, *Z* isomer), 6.37–6.47 (m, 1.5H, Ar*H* + CH=C*H*CONH), 6.60 (d, ^*3*^*J* = 15.7 Hz, 0.5H, CH=C*H*CONH, *Z* isomer), 6.65 (d, ^*3*^*J* = 8.6 Hz, 1H, Ar*H*, *Z* isomer), 6.72–6.82 (m, 2H, Ar*H*), 6.85 (d, ^*3*^*J* = 8.3 Hz, 1H, Ar*H*, *E* isomer), 6.87–6.92 (m, 1H, Ar*H*), 7.03 (d, ^*3*^*J* = 8.5 Hz, 1H, Ar*H*, *E* isomer), 7.05–7.19 (m, 6H, Ar*H*), 7.22 (d, ^*3*^*J* = 8.2 Hz, 1H, Ar*H*, *Z* isomer), 7.27–7.39 (m, 1.5H, Ar*H* + C*H*=CHCONH), 7.47–7.58 (m, 1.5H, Ar*H* + C*H*=CHCONH). ^13^C NMR: (126 MHz, CD_3_OD, *E*/*Z* = 50:50): δ 13.87, 13.92, 25.20, 25.57, 29.90, 30.04, 30.07, 34.72, 40.29, 40.37, 99.95, 113.37, 115.32, 116.07, 116.14, 121.15, 121.62, 127.26, 127.40, 127.86, 128.68, 128.97, 129.03, 130.89, 131.13, 131.71, 132.47, 133.08, 133.58, 134.66, 135.32, 135.64, 139.70, 141.39, 142.87, 143.66, 143,70, 143.92, 146.77, 147.03, 154.61, 155.17, 156.68, 157.58, 168.76, 173.86. HR-MS (*m*/*z*): calcd for C_40_H_42_N_4_O_4_ [M + H]^+^: 643.3279, found: 643.3252.

##### 4.1.3.2.3.5 (*E*)-*N*-[6-(3-(5-Hydroxy-1*H*-benzo[*d*]imidazol-2-yl)propanamido)hexyl]-3-[4-((*E*/*Z*)-1-(4-hydroxyphenyl)-2-phenylbut-1-enyl)phenyl]acrylamide (**43**)

**43** was synthesized according to the general procedure with 130 mg of **38** (0.19 mmol) dissolved in 3.9 mL of dry DCM and 0.68 mL of BBr_3_ solution (0.68 mmol) at 10 °C. The reaction time was 4.5 h. Purification was performed by column chromatography first with EA (100%) and then with a gradient of DCM and MeOH (98:2 → 95:5). **43** was obtained as an off-white powder (15 mg, 0.023 mmol, 12%). Purity: 95.0% (HPLC: 1.0 mM in ACN + water (Na_2_SO_4_, 20 mM (pH 3)), 283 nm). ^1^H NMR: (700 MHz, CD_3_OD, *E*/*Z* = 50:50): δ 0.92 (2 × t, ^*3*^*J* = 7.4 Hz, 3H, 2 × CH_2_C*H*_*3*_), 1.22–1.34 (m, 4H, C*H*_*2*_), 1.40–1.52 (m, 3H, C*H*_*2*_), 1.50 (p, ^*3*^*J* = 7.7 Hz, 1H, C*H*_*2*_, *Z* isomer), 2.47 (q, ^*3*^*J* = 7.4 Hz, 1H, C*H*_*2*_CH_3_, *Z* isomer), 2.52 (q, ^*3*^*J* = 7.5 Hz, 1H, C*H*_*2*_CH_3_, *E* isomer), 2.65–2.72 (2 × t, 2H, 2 × C*H*_*2*_CH_2_CONH), 3.09–3.17 (m, 4H, C*H*_*2*_NH + CH_2_C*H*_*2*_CONH), 3.22 (t, ^*3*^*J* = 7.0 Hz, 1H, GW7604NHC*H*_*2*_, *E* isomer), 3.27 (t, ^*3*^*J* = 7.1 Hz, 1H, GW7604NHC*H*_*2*_, *Z* isomer), 6.41–6.45 (m, 1.5H, Ar*H*, *Z* isomer + CH=C*H*CONH, *E* isomer), 6.61 (d, ^*3*^*J* = 15.8 Hz, 0.5H, CH=C*H*CONH, *Z* isomer), 6.66 (d, ^*3*^*J* = 8.7 Hz, 1H, Ar*H*, *Z* isomer), 6.72–6.74 (2 × dd, ^*4*^*J* = 2.3 Hz, 1H, Ar*H*), 6.78 (d, ^*3*^*J* = 8.5 Hz, 1H, Ar*H*, *E* isomer), 6.84–6.90 (m, 2H, Ar*H*), 7.03 (d, ^*3*^*J* = 8.6 Hz, 1H, Ar*H*, *E* isomer), 7.07–7.20 (m, 6H, Ar*H*), 7.24 (d, ^*3*^*J* = 8.2 Hz, 1H, Ar*H*, *Z* isomer), 7.29–7.31 (m, 1H, Ar*H*), 7.35 (d, ^*3*^*J* = 15.7 Hz, 0.5H, C*H*=CHCONH, *E* isomer), 7.53–7.56 (m, 1.5H, Ar*H* + C*H=*CHCONH, *Z* isomer). ^13^C NMR: (176 MHz, CD_3_OD, *E*/*Z* = 1:1): δ 13.81 *E* isomer, 13.86 *Z* isomer, 25.75, 27.46 *E* isomer, 27.48 *Z* isomer, 27.59 *E* isomer, 27.62 *Z* isomer, 29.85 *Z* isomer, 30.00 *E* isomer, 30.24 *E* isomer, 30.27 *Z* isomer, 30.29 *E* isomer, 30.33 *Z* isomer, 34.89, 40.25 *E* isomer, 40.26 *Z* isomer, 40.42 *Z* isomer, 40.48 *E* isomer, 100.05, 112.85, 115.28 *Z* isomer, 116.03 *E* isomer, 116.14, 121.16 *E* isomer, 121.63 *Z* isomer, 127.21 *Z* isomer, 127.37 *E* isomer, 127.83 *E* isomer, 128.65 *Z* isomer, 128.93 *Z* isomer, 129.00 *E* isomer, 130.84 *Z* isomer, 130.86 *E* isomer, 131.10 *Z* isomer, 131.67 *E* isomer, 132.44 *E* isomer, 133.04 *Z* isomer, 133.60, 134.68 *Z* isomer, 135.30 *Z* isomer, 135.61 *E* isomer, 139.55 *Z* isomer, 139.69 *E* isomer, 141.32, 142.84 *Z* isomer, 143.65 *Z* isomer, 143.68 *E* isomer, 143.89 *E* isomer, 146.73 *E* isomer, 146.99 *Z* isomer, 147.93, 154.68, 154.75, 156.67 *Z* isomer, 157.56 *E* isomer, 168.70, 173.98. HR-MS (*m*/*z*): calcd for C_41_H_44_N_4_O_4_ [M + H]^+^: 657.3435, found: 657.3438.

### 4.2 Cell Culture Experiments

#### 4.2.1 General

The human osteosarcoma cell line U2OS, the hormone-dependent breast cancer cell line MCF-7, the ER-negative breast cancer cell line MDA-MB-231, and the African green monkey kidney cell line COS-7 were obtained from the cell line service (CLS, Eppelheim, Germany). The MCF-7TamR cell line was generated and kindly provided by Cardiff University (Great Britain). The cells were maintained as monolayer cultures. McCoy’s 5A medium supplemented with 10% fetal bovine serum (FBS) (both from Biochrome GmbH, Berlin, Germany) was used for the osteosarcoma cell line, and Dulbecco’s modified Eagle’s medium (DMEM) without phenol red, with glucose (4.5 g/L) (GE Healthcare, Pasching, Austria), supplemented with 10% FBS and 1% pyruvate (GE Healthcare) was used for the breast cancer cell lines. The COS-7 cell line was maintained with DMEM supplemented with 10% FBS. MCF-7TamR were cultured in RPMI medium supplemented with 5% FBS, L*-*glutamine (4 mM), and 4-OHT (10^–7^ M). All cell lines were cultivated in a humidified atmosphere (5% CO_2_/95% air) at 37 °C and passaged twice a week. DMSO was used as the solvent for the investigated compounds. The final concentration of DMSO never exceeded 0.1% in cell-based assays. Vehicle-treated controls were always included.

#### 4.2.2 Binding Assays

LanthaScreen TR-FRET ER alpha/beta competitive binding assays (Invitrogen, Carlsbad) were used to investigate the binding affinity to the isolated receptors according to the manufacturer′s protocols. Measurement was performed with an Enspire multimode plate reader (PerkinElmer Life Sciences, Waltham). Calculations were performed with Excel (Microsoft, Redmond) and GraphPad Prism (GraphPad Software, San Diego).

#### 4.2.3 Cellular Uptake

The determination of the cellular uptake in MCF-7 and COS-7 cells was performed as already described.^24^ Measurement was conducted with the Enspire multimode plate reader. The values represent the mean ± SD of ≥3 independent experiments.

#### 4.2.4 Luciferase Reporter Gene Assay

Transactivation evaluation with respect to ERα/β was performed essentially as previously described.^24^ The transfection reagent (TansIT-LT1, MoBiTec) and the dual-luciferase reporter assay (Promega) were applied according to the manufacturer’s protocol. Renilla luciferase activity was used as the internal control and for normalization. The values represent the mean ± SD of ≥3 independent experiments.

#### 4.2.5 In-Cell Western Immunoassay

Degradation was evaluated with the CellTagTM 700 In-Cell Western kit (LI-COR, Lincoln) according to the manufacturer’s protocol. MCF-7 cells were treated, and calculation was performed as previously described.^24^

#### 4.2.6 Crystal Violet Assay

The cytotoxicity evaluation was performed using MCF-7, MCF-7TamR^49^ (2 × 10^3^ cells per well), and MDA-MB-231 cell lines (1.5 × 10^3^ cells per well) according to a modified protocol previously described.^48^ Cells were seeded in 96-well microtiter plates in DMEM supplemented with 10% charcoal dextran-treated FBS and 1% pyruvate. Twenty-four hours after seeding, the selected compounds, controls, and the vehicle (DMSO) were added at indicated concentrations in quadruples. After an incubation time of 120 h in a humidified atmosphere (5% CO_2_/95% air) at 37 °C, the medium was aspirated, and cells were washed with PBS (GE Healthcare) and fixed with a solution of 1% (v/v) glutaric dialdehyde in PBS. The cell biomass was determined *via* staining of adherent cells with crystal violet, extraction of the stain with ethanol (70%, v/v), and measurement of absorbance at 590 nm. Cell viability is expressed as the percentage of cell viability of the vehicle-treated control, which was set to 100%. Results are the mean ± SD of ≥3 independent experiments.

## Supporting Information Available

^1^H and ^13^C NMR spectra of all final compounds, reversed-phase HPLC chromatograms, additional biological data (PDF)
Molecular formula strings (CSV)

## Abbreviations

4-OHT: 4-hydroxytamoxifen
ACN: acetonitrile
AF2: activation function 2
anh.: anhydrous
Boc: *tert*-butyloxycarbonyl
CABS: coactivator binding site
DCM: dichloromethane
DIPEA: diisopropylamine
DMEM: Dulbecco′s modified Eagle’s medium
DMF: dimethylformamide
DMSO: dimethyl sulfoxide
E2: estradiol
EA: ethyl acetate
equiv: equivalent
ER: estrogen receptor
ESR1: ERα gene alterations
EtOH: ethanol
FBS: fetal bovine serum
GW7604: (*E*/*Z*)-3-(4-((*E*)-1-(4-hydroxyphenyl)-2-phenylbut-1-enyl)phenyl)acrylic acid
H12: Helix 12
HPLC: high-performance liquid chromatography
HR-MS: high-resolution mass spectrometry
LBD: ligand binding domain
LBS: ligand binding site
MeOH: methanol
NMR: nuclear magnetic resonance spectra
PBS: phosphate-buffered saline
PE: petroleum ether
PGC-1: peroxisome proliferator-activated receptor-γ coactivator 1
PPARγ: peroxisome proliferator-activated receptor γ
PyBOP: benzotriazol-1-yl-oxytripyrrolidinophosphonium hexafluorophosphate
RBA: relative binding affinity
SAR: structure–activity relationship
SD: standard deviation
SE: standard error
SERDs: selective ER degraders/downregulators
SERMs: selective ER modulators
TFA: trifluoroacetic acid
THF: tetrahydrofuran
TMS: tetramethylsilane
TR-FRET: time-resolved fluorescence resonance energy transfer
