# Supplementary material for: Heterodimeric GW7604 Derivatives: Modification of the Pharmacological Profile by Additional Interactions at the Coactivator Binding Site
Source: J Med Chem. 2021 Apr 27;64(9):5766–86. doi: 10.1021/acs.jmedchem.0c02230 (PMC8279417; doi:10.1021/acs.jmedchem.0c02230)
Supplement: Supplementary file 1 — jm0c02230_si_001.pdf [file jm0c02230_si_001.pdf]

Supporting Information

Heterodimeric GW7604 Derivatives: Modification of the Pharmacological Profile by Additional Interactions at the Coactivator Binding Site

Alexandra K. Knox,[a] Christina Kalchschmid,[a] Daniela Schuster,[a,b] Francesca Gaggia,[a] and Ronald Gust[a]*

[a] *Department of Pharmaceutical Chemistry, Institute of Pharmacy, CMBI– Center for Molecular Biosciences Innsbruck, University of Innsbruck, CCB – Center for Chemistry and Biomedicine, 6020 Innsbruck, Austria*

[b] *Department of Pharmaceutical and Medicinal Chemistry, Institute of Pharmacy, Paracelsus Medical University, 5020 Salzburg, Austria*

* Corresponding author. E-mail: ronald.gust@uibk.ac.at, Tel: +43-512-507-58200

Table of Contents

1. Synthesis of intermediates .... S 2
2. ^{1}H and ^{13}C NMR spectra of final compounds .... S 12
3. HPLC chromatograms of final compounds .... S 29
4. Coactivator recruitment .... S 44
5. Crystal violet assay .... S 45
6. Transactivation assay .... S 46
7. References .... S 47

1. Synthesis of intermediates

Syntheses of GW7604 and methoxy-GW7604 was performed as described previously.[1]

1.1. Synthesis of thioxo-quinazolinones

General procedure for synthesis of thioxo-quinazolinone carboxylic acids

Modifying the method from Sun et al.[2], 2-methoxycarbonylphenyl isothiocyanate (1 eq) and the respective amino carboxylic acid (1 eq) were dissolved in EtOH and refluxed for 2-3 days. After cooling the solution to ambient temperature and concentrating to half of the volume, the resulting precipitate was filtered off by suction and washed with ice-cold EtOH.

4-(4-Oxo-2-thioxodihydroquinazolin-3-yl)butanoic acid **1**

1 was prepared following the general procedure with 580 mg of 2-methoxycarbonylphenyl isothiocyanate (3.00 mmol), 309 mg of 4-aminobutanoic acid (3.00 mmol) dissolved in 15 mL of EtOH. The mixture was refluxed for 2 days and yielded **1** as a pearl colored powder (538 mg, 2.04 mmol, 68%). ^{1}H NMR: (200 MHz, DMSO-d$_6$): δ 1.94 (p, ^{3}J = 7.1 Hz, 2H, $NCH_2CH_2CH_2COOH$), 2.29 (t, ^{3}J = 7.8 Hz, 2H, $NCH_2CH_2CH_2COOH$), 4.44 (t, ^{3}J = 7.4 Hz, 2H, $NCH_2CH_2CH_2COOH$), 7.29-7.40 (m, 2H, Ar*H*), 7.70-7.78 (m, 1H, Ar*H*), 8.20 (d, ^{3}J = 8.0 Hz, 1H, Ar*H*).

8-(4-Oxo-2-thioxodihydroquinazolin-3-yl)octanoic acid **2**

2 was synthesized according to the general procedure with 386 mg of 2-methoxycarbonylphenyl isothiocyanate (2.00 mmol), 206 mg of 8-aminooctanoic acid (2.00 mmol) dissolved in 10 mL of EtOH. Reaction time was 3 days. **2** was obtained as an off-white powder (380 mg, 1.08 mmol, 56%). ^{1}H NMR: (200 MHz, DMSO-d$_6$): δ 1.28-1.39 (m, 6H, $NCH_2CH_2CH_2CH_2CH_2CH_2CH_2COOH$), 1.29-1.59 (m, 2H, $NCH_2CH_2CH_2CH_2CH_2CH_2CH_2COOH$), 1.59-1.83 (m, 2H, $NCH_2CH_2CH_2CH_2CH_2CH_2CH_2COOH$), 2.19 (t, ^{3}J = 7.2 Hz, 2H, $NCH_2CH_2CH_2CH_2CH_2CH_2$-$CH_2COOH$), 4.38 (t, ^{3}J = 7.2 Hz, 2H, $NCH_2CH_2CH_2CH_2CH_2CH_2$-$CH_2COOH$), 7.29-7.40 (m, 2H, Ar*H*), 7.71-7.78 (m, 1H, Ar*H*), 7.96 (d, ^{3}J = 8.0 Hz, 1H, Ar*H*). HRMS (m/z): calculated for $C_{16}H_{20}N_2O_3S$ [M-H]$^-$: 319.1122, found: 319.1735.

General procedure for synthesis of thioxo-quinazolinone carboxamides

Similar to the previously mentioned general procedures for amide formation, PyBOP (1.0 eq) was dissolved in dry DCM and DIPEA (2.0 eq) together with the respective acid (1.0 eq) in dry DMF were added at 0 °C. After 5 min, the respective amine (1.1 eq) in dry DCM or DMF was supplemented dropwise. The mixture was stirred first at 0 °C for 30 min, then at rt for 20 h. The mixture was concentrated to a residue, which was dissolved in EA, washed with water (pH = 3-4; adjusted with 1N HCl) and further extracted twice with EA. The combined organic layers were washed with brine, dried over dry Na_2SO_4 and evaporated. Purification was carried out by flash column chromatography.

N-[3-(Boc-amino)propyl]-4-[4-oxo-2-thioxodihydroquinazolin-3-yl]butanamide **3**

Synthesis of **3** was carried out according to the general procedure. **1** (120 mg, 0.45 mmol) was dissolved in 2.0 mL of dry DMF. 260 mg of PyBOP (1.1 eq, 0.50 mmol) in 2.0 mL of dry DCM, 0.16 mL of DIPEA (0.91 mmol) and 87 mg of *N*-Boc-1,3-propanediamine (0.50 mmol) in 0.5 mL of dry DCM were added. Flash column chromatography with PE and EA (83:17 → 100% EA) led to a waxy, white powder (175 mg, 0.42 mmol, 92%). ^{1}H NMR: (200 MHz, DMSO-d$_6$): δ 1.36 (s, 9H, COOC(C*H*$_3$)$_3$), 1.44-1.50 (m, 2H, NCH$_2$C*H*$_2$CH$_2$CONH), 1.87-1.99 (m, 2H, NHCH$_2$C*H*$_2$CH$_2$NH), 2.13 (t, ^3J = 7.8 Hz, 2H, NCH$_2$CH$_2$C*H*$_2$CONH), 2.89-2.95 (m, 2H, NHCH$_2$CH$_2$C*H*$_2$NH), 3.00 (q, ^3J = 6.2 Hz, 2H, NHC*H*$_2$CH$_2$CH$_2$NH), 4.41 (t, ^3J = 7.0 Hz, 2H, NC*H*$_2$CH$_2$CH$_2$CONH), 6.74 (t, ^3J = 5.4 Hz, 1H, N*H*), 7.30-7.50 (m, 2H, Ar*H*), 7.70-7.82 (m, 2H, N*H* + Ar*H*), 7.97 (m, 1H, Ar*H*).

N-[4-(Boc-amino)butyl]-8-[4-oxo-2-thioxodihydroquinazolin-3-yl]octanamide **4**

Following the general procedure, **2** (100 mg, 0.31 mmol) was dissolved in 2.0 mL of dry DMF. 162 mg of PyBOP (0.31 mmol) in 2.0 mL of dry DCM, 0.11 mL of DIPEA (0.62 mmol) and 65 mg of *N*-Boc-1,4-butanediamine (0.34 mmol) in 0.5 mL dry DCM were added. Flash column chromatography applying a gradient of PE and EA (3:1 → 100% EA) resulted in a white powder (95 mg, 0.19 mmol, 62%). ^{1}H NMR: (200 MHz, DMSO-d$_6$): δ 1.17-1.36 (m, 19H, C*H*$_2$ + COOC(C*H*$_3$)$_3$), 1.46-1.51 (m, 2H, C*H*$_2$), 1.59-1.67 (m, 2H, C*H*$_2$), 2.03 (t, ^3J = 7.2 Hz, 2H, C*H*$_2$CONH), 2.87-2.89 (m, 2H, C*H*$_2$NHCOOC(CH$_3$)$_3$), 2.98-3.00 (m, 2H, CONHC*H*$_2$), 4.38 (t, ^3J = 7.6 Hz, 2H, NC*H*$_2$), 6.77 (brt, 1H, N*H*), 7.29-7.40 (m, 2H, Ar*H*), 7.70-7.78 (m, 2H, N*H* + *ArH*), 7.96 (d, ^3J = 8.0 Hz, 1H, Ar*H*).

Ethyl 4-[4-(4-(4-oxo-2-thioxodihydroquinazolin-3-yl)butanoyl)piperazin-1-yl]benzoate **5**

5 was synthesized following the general procedure for preparation of thioxo-quinazolinone carboxamides. **1** (381 mg, 1.44 mmol) was dissolved in 4 mL of dry DMF. 0.40 mL of DIPEA (2.27 mmol), 786 mg of PyBOP (1.05 eq, 1.51 mmol) in 8 mL of dry DCM and 355 mg of ethyl 4-(piperazinyl)benzoate in 6 mL of dry DCM were added in this order. After 1 h, the clear solution turned into a cloudy suspension. The next day the solid was filtered by suction and washed with cold MeOH to yield **5** as a shiny white powder (530 mg, 1.1 mmol, 73%). ^{1}H NMR: (200 MHz, DMSO-d$_6$): δ 1.29 (t, ^{3}J = 7.0 Hz, 3H, $COOCH_2CH_3$), 1.97 (p, ^{3}J = 6.6 Hz, 2H, $NHCH_2CH_2CH_2CON$), 2.46 (t, ^{3}J = 7.4 Hz, 2H, $NHCH_2CH_2CH_2CON$), 3.30-3.41 (m, 4H, $CON(CH_2CH_2)_2N$), 4.24 (q, ^{3}J = 7.0 Hz, 2H, $COOCH_2CH_3$), 4.46 (t, ^{3}J = 6.8 Hz, 2H, NCH_2), 6.99 (d, ^{3}J = 8.8 Hz, 1H, Ar*H*), 7.28-7.41 (m, 2H, Ar*H*), 7.70-7.82 (m, 3H, Ar*H*), 7.94 (d, ^{3}J = 7.8 Hz, 1H, Ar*H*).

Ethyl 4-[4-(8-(4-oxo-2-thioxodihydroquinazolin-3-yl)octanoyl)piperazin-1-yl]benzoate **6**

6 was synthesized according to the general procedure for the preparation of thioxo-quinazolinone carboxamides using 372 mg of **2** (1.16 mmol) in 3.5 mL of dry DMF. 0.44 mL (1.25 mmol) of DIPEA, 650 mg of PyBOP (1.25 mmol) in 8 mL of dry DCM and 293 mg of ethyl 4-(piperazinyl)benzoate (1.25 mmol) in 6 mL of dry DCM were added in that order. The next day, water and 2.5N NaOH were added (to adjust pH = 8-9) and the residue was extracted 3× with EA. Then, the combined organic extracts were washed with H_2O/1N HCl (pH = 2-3) whereupon the EA phase turned to cloudy. The aqueous phase was further extracted 2× with EA, then the combined organic extracts were washed with brine, dried over dry Na_2SO_4 and evaporated. The crude product was washed with water and cold MeOH over a sintered glass funnel to remove salts and organic by-products, giving **6** as a white powder (344 mg, 0.64 mmol, 55%). ^{1}H NMR: (200 MHz, DMSO-d$_6$): δ 1.29 (t, ^{3}J = 7.0 Hz, 3H, $COOCH_2CH_3$), 1.30-1.40 (m, 6H, CH_2), 1.41-1.59 (m, 2H, CH_2), 1.59-1.78 (m, 2H, CH_2), 2.34 (t, ^{3}J = 7.2 Hz, 2H, $CH_2CON(CH_2CH_2)_2N$), 3.57-3.68 (m, 4H, $CON(CH_2CH_2)_2N$), 4.24 (q, ^{3}J = 7.0 Hz, 2H, $COOCH_2CH_3$), 4.38 (t, ^{3}J = 7.6 Hz, 2H, Ar-CH_2), 6.98 (d, ^{3}J = 8.8 Hz, 1H, Ar*H*), 7.29-7.41 (m, 2H, Ar*H*), 7.70-7.82 (m, 3H, Ar*H*), 7.94 (d, ^{3}J = 8.0 Hz, 1H, Ar*H*).

4-[4-(4-(4-Oxo-2-thioxodihydroquinazolin-3-yl)butanoyl)piperazin-1-yl]benzoic acid **7**

For ester cleavage, **5** (400 mg, 0.82 mmol) was dissolved in 8 mL of EtOH/THF (1:1) and 4 mL of 2N KOH and the mixture was stirred for 24 h. The solution was concentrated, cooled and 8 mL of 1N HCl were added. The white powder was separated by vacuum filtration (376 mg, 0.82 mmol, quant.). ^{1}H NMR: (200 MHz, DMSO-d$_6$): δ 1.97 (p, ^{3}J = 7.2 Hz, 2H, $NCH_2CH_2CH_2CON$), 2.46 (t, ^{3}J = 7.6 Hz, 2H, $NCH_2CH_2CH_2CON$), 3.22-3.33 (m, 4H, $CON(CH_2CH_2)_2N$), 4.46 (t, ^{3}J = 6.0 Hz, 2H, NCH_2), 6.98 (d, ^{3}J = 8.8 Hz, 1H, Ar*H*), 7.28-7.42 (m, 2H, Ar*H*), 7.71-7.81 (m, 3H, Ar*H*), 7.95 (d, ^{3}J = 8.0 Hz, 1H, Ar*H*).

4-[4-(8-(4-Oxo-2-thioxodihydroquinazolin-3-yl)octanoyl)piperazin-1-yl]benzoic acid **8**

6 (340 mg, 0.82 mmol) was dissolved in 6.8 mL of EtOH/THF (1:1) and 3.4 mL of 2N KOH and the mixture was stirred for 24 h. The solution was concentrated, cooled and 4 mL of 2N HCl were added. The solids were collected by vacuum filtration and gave **8** as a white powder (349 mg, 0.69 mmol, 93%). ^{1}H NMR: (200 MHz, DMSO-d$_6$): δ 1.17-1.39 (m, 6H, C*H$_2$*), 1.39-1.59 (m, 2H, C*H$_2$*), 1.59-1.78 (m, 2H, C*H$_2$*), 2.35 (t, ^3J = 7.2 Hz, 2H, C*H$_2$*CON(CH$_2$CH$_2$)$_2$N), 3.56-3.75 (m, 4H, CON(C*H$_2$*CH$_2$)$_2$N), 4.38 (t, ^3J = 7.7 Hz, 2H, NC*H$_2$*), 6.97 (d, ^3J = 9.2 Hz, 1H, Ar*H*), 7.27-7.41 (m, 2H, Ar*H*), 7.70-7.80 (m, 3H, Ar*H*), 7.96 (d, ^3J = 7.4 Hz, 1H, Ar*H*), 12.39 (brs, 2H, COO*H* + N*H*).

N-3-(Boc-aminopropyl)-4-[4-(4-(4-oxo-2-thioxodihydroquinazolin-3-yl)butanoyl)piperazin-1-yl]benz-amide **9**

Following the general procedure for the synthesis of thioxo-quinazolinone carboxamides, **7** (200 mg, 0.44 mmol) was dissolved in 3 mL of dry DMF and 0.15 mL of DIPEA (0.88 mmol) was added, leading to an intense yellow colored solution. The color changed to red after addition of 230 mg (0.44 mmol) of PyBOP dissolved in 4 mL of dry DCM. At last, *N*-Boc-1,3-propanediamine (71 mg, 0.44 mmol) dissolved in 3 mL of dry DCM was added. The next day, the reaction was quenched with water whereupon the red color faded. The resulting precipitate was filtered off by vacuum suction and washed with cold MeOH (145 mg, 0.24 mmol, 54%). ^{1}H NMR: (200 MHz, DMSO-d$_6$): δ 1.37 (s, 9H, COOC(C*H$_3$*)$_3$), 1.56 (p, ^3J = 7.0 Hz, 2H, NHCH$_2$C*H$_2$*CH$_2$NH), 1.97 (p, ^3J = 6.6 Hz, 2H, NCH$_2$C*H$_2$*CH$_2$CON), 2.46 (t, ^3J = 7.0 Hz, 2H, NCH$_2$CH$_2$C*H$_2$*CON), 2.95 (q, ^3J = 6.2 Hz, 2H, NHCH$_2$CH$_2$C*H$_2$*NH), 3.23-3.35 (m, 4H, CON(C*H$_2$*CH$_2$)$_2$N), 3.54-3.66 (m, 4H, CON(CH$_2$C*H$_2$*)$_2$N), 4.46 (t, ^3J = 6.8 Hz, 2H, NC*H$_2$*), 6.81 (brt, 1H, N*H*), 6.97 (d, ^3J = 8.8 Hz, 2H, Ar*H*), 7.28-7.40 (m, 2H, Ar*H*), 7.71-7.75 (m, 3H, Ar*H*), 7.94 (d, ^3J = 7.6 Hz, 1H, Ar*H*), 8.18 (brt, 1H, N*H*).

N-3-(Boc-aminopropyl)-4-[4-(8-(4-oxo-2-thioxodihydroquinazolin-3-yl)octanoyl)piperazin-1-yl]benz-amide **10**

10 was synthesized according to the general procedure for the synthesis of thioxo-quinazolinone carboxamides. 0.15 mL of DIPEA (0.87 mmol, 2 eq), 225 mg of PyBOP (0.43 mmol) in 4 mL of dry DCM and 76 mg of *N*-Boc-1,3-propanediamine (0.43 mmol) in 3 mL of dry DCM were added to 220 mg of **8** (0.43 mmol) in 3 mL of dry DMF. The following day, water and 2.5N NaOH were added (pH = 8-9) and the residue was extracted 3× with EA. Then, the combined organic extracts were washed with water/1N HCl (pH = 2-3). The aqueous phase was further extracted twice with EA and the combined organic extracts were evaporated to dryness. Purification was carried out by flash column chromatography applying a PE:EA:MeOH gradient (EA: 24% → 100%, then MeOH 0% → 5%), which afforded **10** as a white foam

(177 mg, 0.27 mmol, 62%). ^{1}H NMR: (200 MHz, DMSO-d$_6$): δ 1.28-1.36 (m, 6H, C*H$_2$*), 1.37 (s, 9H, COOC(C*H$_3$*)$_3$), 1.47-1.66 (m, 6H, C*H$_2$*), 2.35 (t, ^3J = 7.6 Hz, 2H, C*H$_2$*CON(CH$_2$CH$_2$)$_2$N), 2.89-3.00 (m, 2H, NHCH$_2$CH$_2$C*H$_2$*NH), 3.14-3.37 (m, 6H, C*H$_2$*), 3.53-3.66 (m, 4H, CON(CH$_2$C*H$_2$*)$_2$N), 4.39 (t, ^3J = 8.0 Hz, 2H, NC*H$_2$*), 6.81 (t, ^3J = 5.4 Hz, 2H, N*H*), 6.96 (d, ^3J = 8.8 Hz, 2H, Ar*H*), 7.29-7.41 (m, 2H, Ar*H*), 7.71-7.78 (m, 3H, N*H* + Ar*H*), 7.95 (d, ^3J = 7.0 Hz, 1H, Ar*H*), 8.17 (t, ^3J = 5.6 Hz, 1H, N*H*).

3-[4-(4-Oxo-2-thioxo-dihydroquinazolin-3-yl)butanamido]propan-1-aminium trifluoroacetate **11**

According to the general procedure for Boc-deprotection of amines (see 8.1.2.3), 160 mg of **3** (0.38 mmol) were suspended in 6 mL of dry DCM and 0.44 mL of TFA (5.71 mmol, 15 eq) were added dropwise. **11** was obtained as white powder (160 mg, 0.37 mmol, 99%). ^{1}H NMR: (200 MHz, DMSO-d$_6$): δ 1.66 (p, ^3J = 7.0 Hz, 2H, C*H$_2$*), 1.85-1.96 (m, 2H, C*H$_2$*), 2.17 (t, ^3J = 7.3 Hz, 2H, NCH$_2$CH$_2$CH$_2$C*H$_2$*CONH), 2.73-2.83 (m, 2H, NHCH$_2$CH$_2$C*H$_2$*NH$_3^+$), 3.10 (q, ^3J = 6.2 Hz, 2H, NHC*H$_2$*CH$_2$CH$_2$NH), 4.42 (t + brs, ^3J = 7.2 Hz, 5H, NC*H$_2$* + N*H$_3^+$*), 7.31-7.45 (m, 2H, Ar*H*), 7.59-7.79 (m, 2H, Ar*H* + N*H*), 7.95-8.04 (m, 2H, Ar*H* + N*H*).

4-[8-(4-Oxo-2-thioxodihydroquinazolin-3-yl)octanamido]butan-1-aminium trifluoroacetate **12**

According to the general procedure for Boc-deprotection of amines, 90 mg of **4** (0.18 mmol) were suspended in 3 mL of dry DCM and 0.26 mL of TFA (2.7 mmol, 18 eq) were slowly added whereupon the suspension turned clear. Workup resulted in a white solid (90 mg, 0.18 mmol, 99%). ^{1}H NMR: (200 MHz, DMSO-d$_6$): δ 1.24-1.46 (m, 12H, C*H$_2$*), 1.60-1.81 (m, 2H, C*H$_2$*), 2.05 (t, ^3J = 7.0 Hz, 2H, C*H$_2$*CONH), 2.80 (t, ^3J = 6.6 Hz, 2H, C*H$_2$*NH$_3^+$), 3.04 (q, ^3J = 5.8 Hz, 2H, CONHC*H$_2$*), 4.38 (t, ^3J = 7.8 Hz, 2H, NC*H$_2$*), 7.31-7.41 (m, 2H, Ar*H*), 7.51-7.82 (m, Ar*H* + N*H$_3^+$*), 7.40 (d,^3J = 7.8 Hz, 1H, Ar*H*).

3-[4-(4-(4-(4-Oxo-2-thioxodihydroquinazolin-3-yl)butanoyl)piperazin-1-yl)benzamido]propan-1-aminium trifluoroacetate **13**

13 was synthesized following the general procedure for Boc-deprotection with 140 mg of **9** (0.23 mmol), 4 mL of dry DCM and 0.27 mL of TFA (3.45 mmol, 15 eq). A white solid was obtained (143 mg, 0.23 mmol, quant.). ^{1}H NMR: (200 MHz, DMSO-d$_6$): δ 1.78 (p, ^3J = 6.8 Hz, 2H, NHCH$_2$C*H$_2$*CH$_2$NH$_3^+$), 1.98 (p, ^3J = 7,3 Hz, 2H, NCH$_2$C*H$_2$*CH$_2$CON), 2.40-2.51 (m, 2H, NCH$_2$CH$_2$C*H$_2$*CON), 2.82 (q, ^3J = 7.0 Hz, NHCH$_2$CH$_2$C*H$_2$*NH$_3^+$), 3.17-3.32 (m, 6H, CON(C*H$_2$*CH$_2$)$_2$N + NHC*H$_2$*CH$_2$CH$_2$NH$_3^+$), 3.66-3.81 (m, 4H, CON(CH$_2$C*H$_2$*)$_2$N), 4.46 (t, ^3J = 7.2 Hz, 1H, NC*H$_2$*CH$_2$CH$_2$CON), 6.97 (d, ^3J = 8.4 Hz, 2H, Ar*H*), 7.29-7.41 (m, 2H, Ar*H*), 7.62-7.83 (m, 6H, Ar*H* + N*H$_3^+$*), 7.95 (d, ^3J = 7.6 Hz, 1H, Ar*H*), 8.38 (t, ^3J = 5.8 Hz, 1H, N*H*).

3-[4-(4-(8-(4-Oxo-2-thioxodihydroquinazolin-3-yl)octanoyl)piperazin-1-yl)benzamido]propan-1-aminium trifluoroacetate **14**

14 was synthesized according to the general procedure for Boc-deprotection, using 160 mg of **10** (0.24 mmol), 4.5 mL of dry DCM and an excess of TFA (1.0 mL). Workup resulted in a white powder (163 mg, 0.24 mmol, quant.). ^{1}H NMR: (200 MHz, DMSO-d_6): δ 1.24-1.41 (m, 6H, CH_2), 1.51-1.81 (m, 6H, CH_2), 2.35 (t, ^3J = 7.4 Hz, 2H, CH_2CON(CH_2CH_2)$_2$N)), 2.80-2.89 (m, 2H, NHCH_2CH_2CH_2NH_3^+), 3.26-3.31 (m, 6H, CON(CH_2CH_2)$_2$N + NHCH_2CH_2CH_2NH_3^+), 3.49-3.68 (m, 4H, CON(CH_2CH_2)$_2$N), 6.98 (d, ^3J = 8.8 Hz, 2H, ArH), 7.30-7.41 (m, 2H, ArH), 7.72-7.78 (m, 6H, ArH, + NH_3^+), 7.96 (d, ^3J = 7.4 Hz, 1H, ArH), 8.38 (brt, 1H, NH).

1.2. Synthesis of 1*H*-benzo[*d*]imidazoles

Ethyl 3-(5-methoxy-1H-benzo[d]imidazole-2-yl)propanoate **19**

19 was prepared adopting a described procedure.[3] 4-Methoxybenzene-1,2-diamine (1.0 eq, 5.00 g, 36.2 mmol), succinic anhydride (1.2 eq, 4.35 g, 43.5 mmol) and dioxane (90 mL) were mixed and heated to 80 °C for 24 h. After cooling to rt, the mixture was concentrated to a sticky residue, which was dissolved in EtOH (90 mL). Concentrated H_2SO_4 (2 mL) was subsequently added dropwise and the mixture was heated again for 24 h to 80 °C. After cooling, the solution was concentrated, water was added (80 mL), the pH was adjusted to 8-9 with 1 N NaOH and extracted three times with DCM (100 mL). The combined organic layers were washed with water (20 mL), brine, dried over Na_2SO_4 and evaporated. The crude product was dissolved in DCM, adsorbed on silica and purified by flash chromatography with DCM and MeOH (98:2 → 84:16) to yield **19** as a red-brown sticky solid (3.90 g, 15.6 mmol, 43%). ^{1}H NMR: (200 MHz, CD_3OD): δ 1.20 (t, ^3J = 7.4 Hz, 3H, OCH$_2$CH_3), 2.87 (t, ^3J = 7.2 Hz, 2H, CH_2CH$_2$COCH$_2$CH$_3$), 3.14 (t, ^3J = 6.8 Hz, 2H, CH$_2$CH_2COCH$_2$CH$_3$), 3.82 (s, 3H, OCH_3), 4.12 (q, ^3J = 6.8 Hz, 3H, OCH_2CH$_3$), 6.82 (dd, ^3J = 8.8 Hz, ^4J = 2.4 Hz, 1H, ArH_6), 7.00 (d, ^4J = 2.6 Hz, 1H, ArH_4), 7.36 (d, ^3J = 8.8 Hz, 1H, ArH_7). ^{13}C NMR: (50 MHz, CD_3OD): δ 14.48, 25.03, 33.00, 56.20, 61.81, 98.46, 112.71, 157.84, 173.85.

3-(5-Methoxy-1H-benzo[d]imidazol-2-yl)propanoic acid hydrochloride **20**

19 (780 mg, 3.14 mmol) was dissolved in 2 N HCl (13 mL) and heated to reflux for 4 h.[4] Removal of water under reduced pressure led to **20** as dark brown crystals (806 mg, 3.14 mmol, quant.). ^{1}H NMR: (200 MHz, CD_3OD): δ 3.00 (t, ^3J = 6.8 Hz, 2H, CH_2CH$_2$COOH), 3.37 (t, ^3J = 6.0 Hz, 2H, CH$_2$CH_2COOH), 3.90 (s, 3H, OCH_3), 7.14-7.20 (m, 2H, ArH_6, ArH_4), 7.61 (d, ^3J = 8.7 Hz, 1H, ArH_7).

General procedure for benzimidazole monoamide formation

Benzimidazole monoamide formation was performed based on the general procedure for homo diamide formation and was further optimized regarding temperate and work up. PyBOP (1.1 eq) dissolved in dry DCM was added to a solution of **20** (1.0 eq) in dry DMF at 0 °C under an argon atmosphere. The mixture was stirred for 5 min. Then, DIPEA (6.0 eq) was added dropwise followed by the addition of the respective mono *N*-Boc-protected diamine (1.0 eq) in dry DMF. The mixture was stirred first at 0 °C for 30 min, then at 40 °C for 20 h. Afterwards, the solvents were concentrated and the residue was dissolved in EA, washed with water (pH = 8-9; adjusted with 2N NaOH if needed), dried over Na_2SO_4 and evaporated. Purification was achieved by column chromatography with DCM and MeOH as eluent (93:7) followed by crystallization from MeOH/water.[5-8]

N-[2-(Boc-amino)ethyl]-3-(5-methoxy-1H-benzo[d]imidazole-2-yl)propanamide **21**

21 was synthesized following the general procedure with 400 mg of **20** (1.56 mmol) in 2 mL of dry DMF, 315 mg of *N*-Boc-1,2-diaminoethane (1.56 mmol) in 0.4 mL of dry DMF, 1.52 mL of DIPEA (8.58 mmol) and 893 mg of PyBOP (1.72 mmol) in 4 mL of dry DCM. **21** was obtained as a pearl-colored powder (491 mg, 1.35 mmol, 87%). ^{1}H NMR: (200 MHz, CD_3OD): δ 1.41 (s, 9H, COOC(C*H₃*)₃), 2.72 (t, ^3J = 7.4 Hz, 2H, $C\mathit{H_2}CH_2CONH$), 3.11-3.18 (m, 4H, $CH_2C\mathit{H_2}CONH$, $CH_2C\mathit{H_2}NHCOOC(CH_3)_3$), 3.21-3.27 (m, 2H, $C\mathit{H_2}CH_2NHCOOC(CH_3)_3$), 3.82 (s, 3H, $OC\mathit{H_3}$), 6.61 (brs, 1H, $N\mathit{H}COOC(CH_3)_3$), 6.86 (dd, ^3J = 8.8 Hz, ^4J = 2.4 Hz, 1H, $Ar\mathit{H_6}$), 7.01 (d, ^4J = 2.2 Hz, 1H, $Ar\mathit{H_4}$), 7.39 (d, ^3J = 8.8 Hz, 1H, $Ar\mathit{H_7}$).

N-[3-(Boc-amino)propyl]-3-(5-methoxy-1H-benzo[d]imidazol-2-yl)propanamide **22**

22 was synthesized applying 160 mg of **20** (0.63 mmol) in 1.3 mL of dry DMF, 109 mg of *N*-Boc-1,3-diaminopropane (0.63 mmol) in 0.4 mL of dry DMF, 0.66 mL of DIPEA (3.8 mmol) and 358 mg of PyBOP (0.69 mmol) in 3.5 mL of dry DCM. **22** was obtained as a white powder (130 mg, 0.35 mmol, 55%). ^{1}H NMR: (200 MHz, CD_3OD): δ 1.43 (s, 9H, $COOC(C\mathit{H_3})_3$), 1.58 (p, ^3J = 6.6 Hz, 2H, $CH_2C\mathit{H_2}CH_2NHCOOC(CH_3)_3$), 2.70 (t, ^3J = 7.5 Hz, 2H, $C\mathit{H_2}CH_2CONH$), 2.98 (t, ^3J = 6.6 Hz 2H, $CH_2C\mathit{H_2}CONH$), 3.13 (t, ^3J = 7.8 Hz, 2H, $CH_2CH_2C\mathit{H_2}NHCOOC(CH_3)_3$), 3.19 (t, ^3J = 6.8 Hz, 2H, $C\mathit{H_2}CH_2CH_2NHCOOC(CH_3)_3$), 3.81 (s, 3H, $OC\mathit{H_3}$), 6.53 (brs, 1H, $N\mathit{H}COOC(CH_3)_3$), 6.82 (dd, ^3J = 8.8 Hz, ^4J = 2.2 Hz, 1H, $Ar\mathit{H_6}$), 6.99 (d, ^4J = 2.1 Hz, 1H, $Ar\mathit{H_4}$), 7.36 (d, ^3J = 8.8 Hz, 1H, $Ar\mathit{H_7}$).

N-[4-(Boc-amino)butyl]-3-(5-methoxy-1H-benzo[d]imidazol-2-yl)propanamide **23**

23 was synthesized using 215 mg of **20** (0.84 mmol) in 1.3 mL of dry DMF, 158 mg of *N*-Boc-1,4-diaminobutane (0.84 mmol) in 0.4 mL of dry DMF, 0.89 mL of DIPEA (5.0 mmol) and 523 mg of PyBOP (1.01 mmol) in 3 mL of dry DCM. **23** was obtained as a beige powder (210 mg, 0.54 mmol, 64%). ^{1}H NMR: (200 MHz, CD_3OD): δ 1.35-1.42 (m, 13H, $CH_2\mathit{CH_2}\mathit{CH_2}CH_2NHCOOC(\mathit{CH_3})_3$), 2.70 (t, ^3J = 7.5 Hz, 2H, $\mathit{CH_2}CH_2CONH$), 2.97 (t, ^3J = 6.1 Hz, 2H, $CH_2\mathit{CH_2}CONH$), 3.09-3.19 (m, 4H, $\mathit{CH_2}CH_2CH_2\mathit{CH_2}NCOC(\mathit{CH_3})_3$), 3.82 (s, 3H, $O\mathit{CH_3}$), 6.83 (dd, ^3J = 8.8 Hz, ^4J = 2.3 Hz, 1H, $Ar\mathit{H_6}$), 7.00 (d, ^4J = 2.2 Hz, 1H, $Ar\mathit{H_4}$), 7.37 (d, ^3J = 8.8 Hz, 1H, $Ar\mathit{H_7}$).

N-[5-(Boc-amino)pentyl]-3-(5-methoxy-1H-benzo[d]imidazol-2-yl)propanamide **24**

24 was prepared using 250 mg of **20** (0.97 mmol) in 1.5 mL of dry DMF, 197 mg of *N*-Boc-1,5-diaminopentane (0.97 mmol) in 0.4 mL of dry DMF, 1.03 mL of DIPEA (5.84 mmol) and 608 mg of PyBOP (1.17 mmol) in 4 mL of dry DCM. Column chromatography with a DCM and MeOH gradient (95:5 → 93:7) and crystallization (acetone/water) led to **20** as a pearl-brownish powder (170 mg, 0.42 mmol (43%). ^{1}H NMR: (200 MHz, CD_3OD): δ 1.30-1.55 (m, 15H, $CH_2\mathit{CH_2}\mathit{CH_2}\mathit{CH_2}CH_2NHCOOC(\mathit{CH_3})_3$), 2.73 (t, ^3J = 7.0 Hz, 2H, $\mathit{CH_2}CH_2CONH$), 2.88-3.27 (m, 6H, $\mathit{CH_2}CH_2CH_2CH_2\mathit{CH_2}NHCOOC(CH_3)_3$, $CH_2\mathit{CH_2}CONH$), 3.80 (s, 3H, $O\mathit{CH_3}$), 5.93 (brs, 1H, $N\mathit{H}COOC(CH_3)_3$), 6.79 (dd, ^3J = 8.7 Hz, ^4J = 2.4 Hz, 1H, $Ar\mathit{H_6}$), 7.06 (d, ^4J = 2.4 Hz, 1H, $Ar\mathit{H_4}$), 7.18 (brs, 1H, $CH_2CH_2CON\mathit{H}$), 7.40 (d, ^3J = 8.7 Hz, 1H, $Ar\mathit{H_7}$).

N-[6-(Boc-amino)hexyl]-3-(5-methoxy-1H-benzo[d]imidazol-2-yl)propanamide **25**

25 was synthesized using 250 mg of **20** (0.97 mmol) in 1.5 mL of dry DMF, 211 mg of *N*-Boc-1,6-diaminohexane (0.97 mmol) in 0.4 mL dry DMF, 1.03 mL of DIPEA (5.84 mmol) and 608 mg of PyBOP (1.17 mmol) in 4 mL of dry DCM. Column chromatography and crystallization from acetone/water led to **25** as beige crystals (185 mg, 0.44 mmol, 45%). ^{1}H NMR: (200 MHz, CD_3OD): δ 1.22-1.38 (m, 4H, $\mathit{CH_2}$), 1.39-1.51 (m, 13H, $\mathit{CH_2}$ + $COOC(\mathit{CH_3})_3$), 2.73 (t, ^3J = 6.8 Hz, 2H, $\mathit{CH_2}CH_2CONH$), 2.97-3.07 (m, 2H, $CH_2\mathit{CH_2}CONH$), 3.12-3.23 (m, 4H, $\mathit{CH_2}CH_2CH_2CH_2CH_2\mathit{CH_2}NHCOOC(CH_3)_3$), 3.80 (s, 3H, $O\mathit{CH_3}$), 5.92 (brs, 1H, $N\mathit{H}COOC(CH_3)_3$), 6.80 (dd, ^3J = 8.8 Hz, ^4J = 2.4 Hz, 1H, $Ar\mathit{H_6}$), 7.06 (d, ^4J = 2.4 Hz, 1H, $Ar\mathit{H_4}$), 7.40 (d+brs, ^3J = 8.8 Hz, 2H, $Ar\mathit{H_7}$ + $CH_2CH_2CON\mathit{H}$).

General procedure for Boc-deprotection of amines

N-Boc cleavage was performed revising literature procedures.[7, 9] TFA was added to the protected amine in dry DCM (DCM:TFA = 3:1) under an argon atmosphere at 0 °C. The reaction mixture was stirred for 2 h at 0 °C followed by the evaporation of the solvent under reduced pressure. The residue was treated with

MeOH and DCM several times to remove remaining TFA and then evaporated to dryness affording a brownish oil.

N-(2-Aminoethyl)-3-(5-methoxy-1H-benzo[d]imidazol-2-yl)propanamide bis(trifluoroacetate) salt **26**

3.0 mL of TFA were added to 450 mg of **21** (1.24 mmol) in 9 mL of dry DCM (608 mg, 1.24 mmol, quant.). ^{1}H NMR: (200 MHz, DMSO-d$_6$): δ 2.66-2.98 (m, 4H, CH_2CH_2CONH), 3.17-3.41 (m, 4H, CH_2CH_2NH$_3^+$), 3.96 (s, 3H, OCH_3), 5.31 (brs, 3H, NH_3^+), 7.11 (dd, ^3J = 9.0 Hz, ^4J = 2.0 Hz, 1H, ArH_6), 7.23 (d, ^4J = 2.0 Hz, 1H, ArH_4), 7.66 (d, ^3J = 8.9 Hz, 1H, ArH_7), 7.86 (brs, 2H, NH^+), 8.31 (brt, 1H, CONHCH$_2$).

N-(3-Aminopropyl)-3-(5-methoxy-1H-benzo[d]imidazol-2-yl)propanamide bis(trifluoroacetate) salt **27**

1.7 mL of TFA were added to 140 mg of **22** (0.37 mmol) in 5 mL of dry DCM (187 mg, 0.37 mmol, quant.). ^{1}H NMR: (400 MHz, (CD$_3$)$_2$CO): δ 1.98 (p, ^3J = 6.8 Hz, 2H, CH$_2$CH_2CH$_2$NH$_3^+$), 2.41-2.68 (m, 2H, NH), 3.00 (t, ^3J = 6.8 Hz, 2H, CH_2CH$_2$CONH), 3.32 (t, ^3J = 6.1 Hz, 2H, CH$_2$CH_2CONH), 3.53 (t, ^3J = 6.7 Hz, 2H, CH$_2$CH$_2$CH_2NH$_3^+$), 3.83 (t, ^3J = 6.9 Hz, 2H, CH_2CH$_2$CH$_2$NH$_3^+$), 3.89 (s, 3H, OCH_3), 7.14 (dd, ^3J = 9.0 Hz, ^4J = 2.4 Hz, 1H, ArH_7), 7.35 (d, ^4J = 2.3 Hz, 1H, ArH_2), 7.72 (d, ^3J = 9.0 Hz, 1H, ArH_6), 8.14 (brs, 1H, NH). ^{1}H NMR: (400 MHz, DMSO-d$_6$): δ 1.66 (p, ^3J = 7.0 Hz, 2H, CH$_2$CH_2CH$_2$NH$_3^+$), 2.67-2.88 (m, 4H, CH_2), 3.10 (q, ^3J = 6.7 Hz, 2H, CH_2), 3.27 (t, ^3J = 7.0 Hz, 2H, CH_2), 3.85 (s, 3H, OCH_3), 7.12 (dd, ^3J = 9.0 Hz, ^4J = 2.4 Hz, 1H, ArH_6), 7.24 (d, ^4J = 2.3 Hz, 1H, ArH_4), 7.67 (d, ^3J = 9.0 Hz, 1H, ArH_7), 7.78 (brs, 2H, NH^+), 8.22 (t, ^3J = 5.6 Hz 1H, NH).

N-(4-Aminobutyl)-3-(5-methoxy-1H-benzo[d]imidazol-2-yl)propanamide bis(trifluoroacetate) salt **28**

2.2 mL of TFA were added to 206 mg of **23** (0.53 mmol) in 7 mL of dry DCM (273 mg, 0.53 mmol, quant.). ^{1}H NMR: (200 MHz, (CD$_3$)$_2$CO): δ 1.53 (p, 2H, ^3J = 6.3 Hz, CH$_2$CH_2CH$_2$CH$_2$NH$_3^+$), 1.70 (p, 2H, ^3J = 6.2 Hz, CH$_2$CH$_2$CH_2CH$_2$NH$_3^+$), 2.44-2.58 (m, 2H, NH), 2.96 (t, ^3J = 7.3 Hz, 2H, CH_2CH$_2$CONH), 3.19 (t, ^3J = 6.1 Hz, 2H, CH$_2$CH_2CONH), 3.50 (t, ^3J = 7.3 Hz, 2H CH_2CH$_2$CH$_2$CH$_2$NH$_3^+$), 3.73 (t, ^3J = 7.0 Hz, 2H CH$_2$CH$_2$CH$_2$CH_2NH$_3^+$), 3.87 (s, 3H, OCH_3), 7.10 (dd, ^3J = 9.0 Hz, ^4J = 2.4 Hz, 1H, ArH_6), 7.36 (d, ^4J = 2.3 Hz, 1H, ArH_4), 7.70 (d, ^3J = 9.0 Hz, 1H, ArH_7), 7.96 (brs, 1H, NH).

N-(5-Aminopentyl)-3-(5-methoxy-1H-benzo[d]imidazol-2-yl)propanamide bis(trifluoroacetate) salt **29**

1.8 mL of TFA were added to 156 mg of **24** (0.39 mmol) in 5 mL of dry DCM (208 mg, 0.39 mmol, quant.). ^{1}H NMR: (200 MHz, (CD$_3$)$_2$CO: δ 1.39-1.52 (m, 4H, CH$_2$CH_2CH_2CH$_2$CH$_2$NH$_3^+$), 1.75-1.86 (m, 2H, CH$_2$CH$_2$CH$_2$CH_2CH$_2$NH$_3^+$), 2.97 (t, ^3J = 7.7 Hz, 2H, CH_2CH$_2$CONH), 3.18-3.20 (m, 2H, CH$_2$CH_2CONH), 3.51 (t, ^3J = 7.6 Hz, 2H, CH_2CH$_2$CH$_2$CH$_2$CH$_2$NH$_3^+$), 3.73 (t, ^3J = 6.8 Hz, 2H, CH$_2$CH$_2$CH$_2$CH$_2$CH_2NH$_3^+$),

3.89 (s, 3H, OCH_3), 7.14 (dd, ^3J = 8.9 Hz, ^4J = 2.2 Hz, 1H, ArH_6), 7.36 (d, ^4J = 2.4 Hz, 1H, ArH_4), 7.71 (d, ^3J = 9.4 Hz, 1H, ArH_7), 7.73 (brs, 1H, NH).

N-(6-Aminohexyl)-3-(5-methoxy-1H-benzo[d]imidazol-2-yl)propanamide bis(trifluoroacetate) salt **30**

2 mL of TFA were added to 251 mg of **25** (0.60 mmol) in 8 mL of dry DCM (327 mg, 0.60 mmol, quant.). ^{1}H NMR: (200 MHz, $(CD_3)_2CO$): δ 1.15-1.23 (m, 2H, $CH_2CH_2CH_2CH_2CH_2CH_2NH_3^+$), 1.29-1.45 (m, 4H, $CH_2CH_2CH_2CH_2CH_2CH_2NH_3^+$), 1.60-1.70 (m, 2H, $CH_2CH_2CH_2CH_2CH_2CH_2NH_3^+$), 2.96 (t, ^3J = 6.9 Hz, 2H, CH_2CH_2CONH), 3.07-3.22 (m, 2H, CH_2CH_2CONH), 3.51 (t, ^3J = 6.9 Hz, 2H, $CH_2CH_2CH_2CH_2CH_2CH_2NH_3^+$), 3.71 (t, ^3J = 6.9 Hz, 2H, $CH_2CH_2CH_2CH_2CH_2CH_2NH_3^+$), 3.88 (s, 3H, OC$H_3$), 7.11 (dd, ^3J = 9.0 Hz, ^4J = 2.4 Hz, 1H, ArH_6), 7.37 (d, ^4J = 2.3 Hz, 1H, ArH_4), 7.72 (d, ^3J = 9.0 Hz, 2H, ArH_7), 7.98 (brs, 1H, NH).

2. ^{1}H and ^{13}C NMR spectra of final compounds.

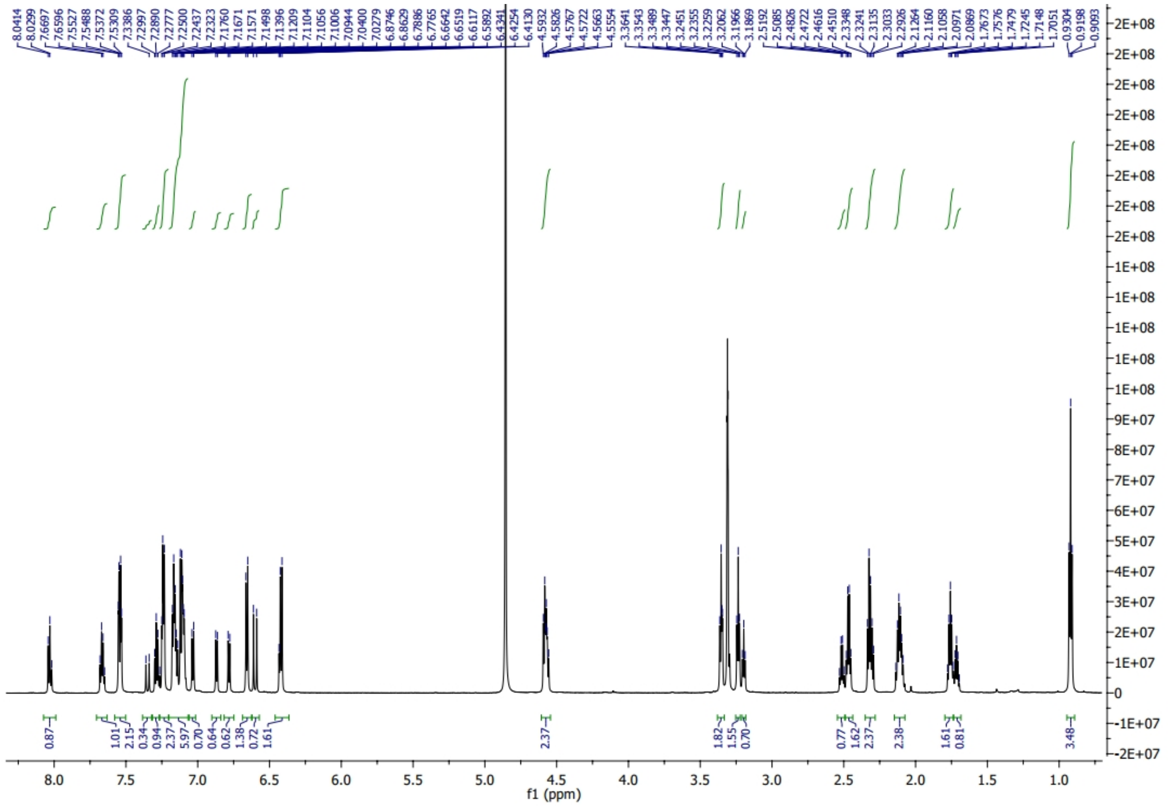

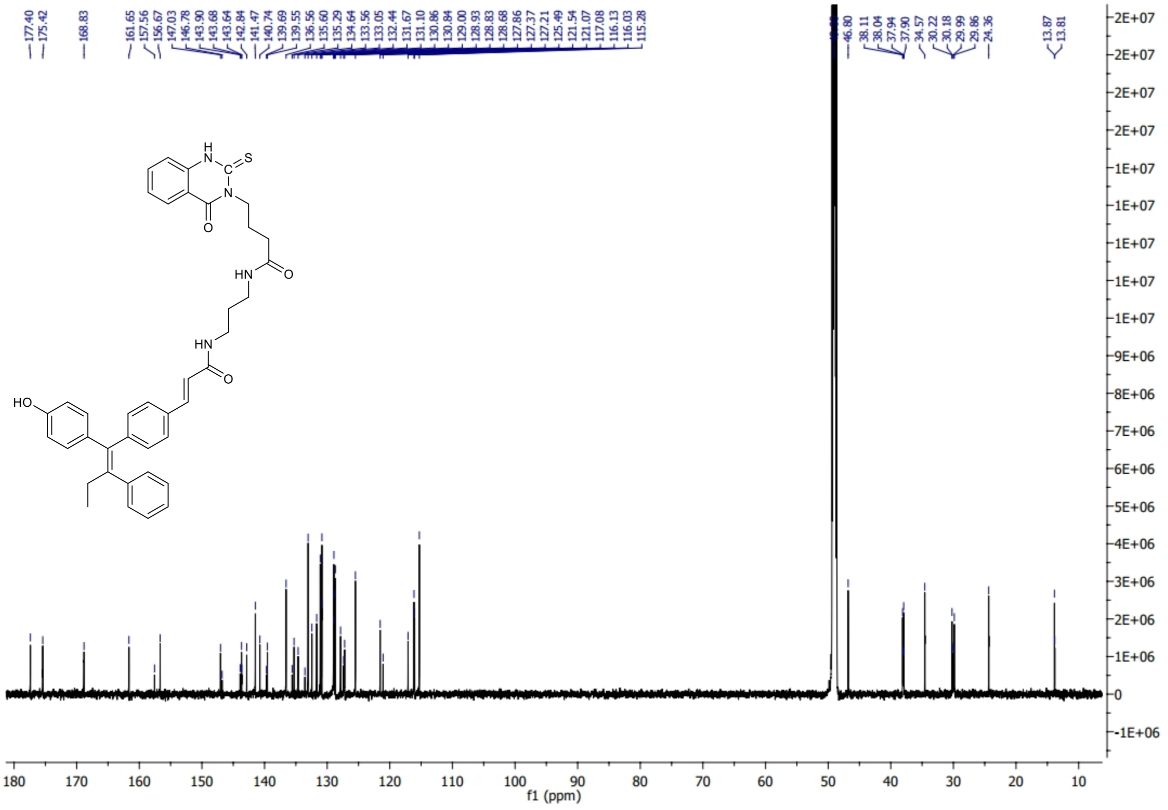

Figure S1: ^{1}H (700 MHz) and ^{13}C NMR (176 MHz) of compound **15** in CD_3OD.

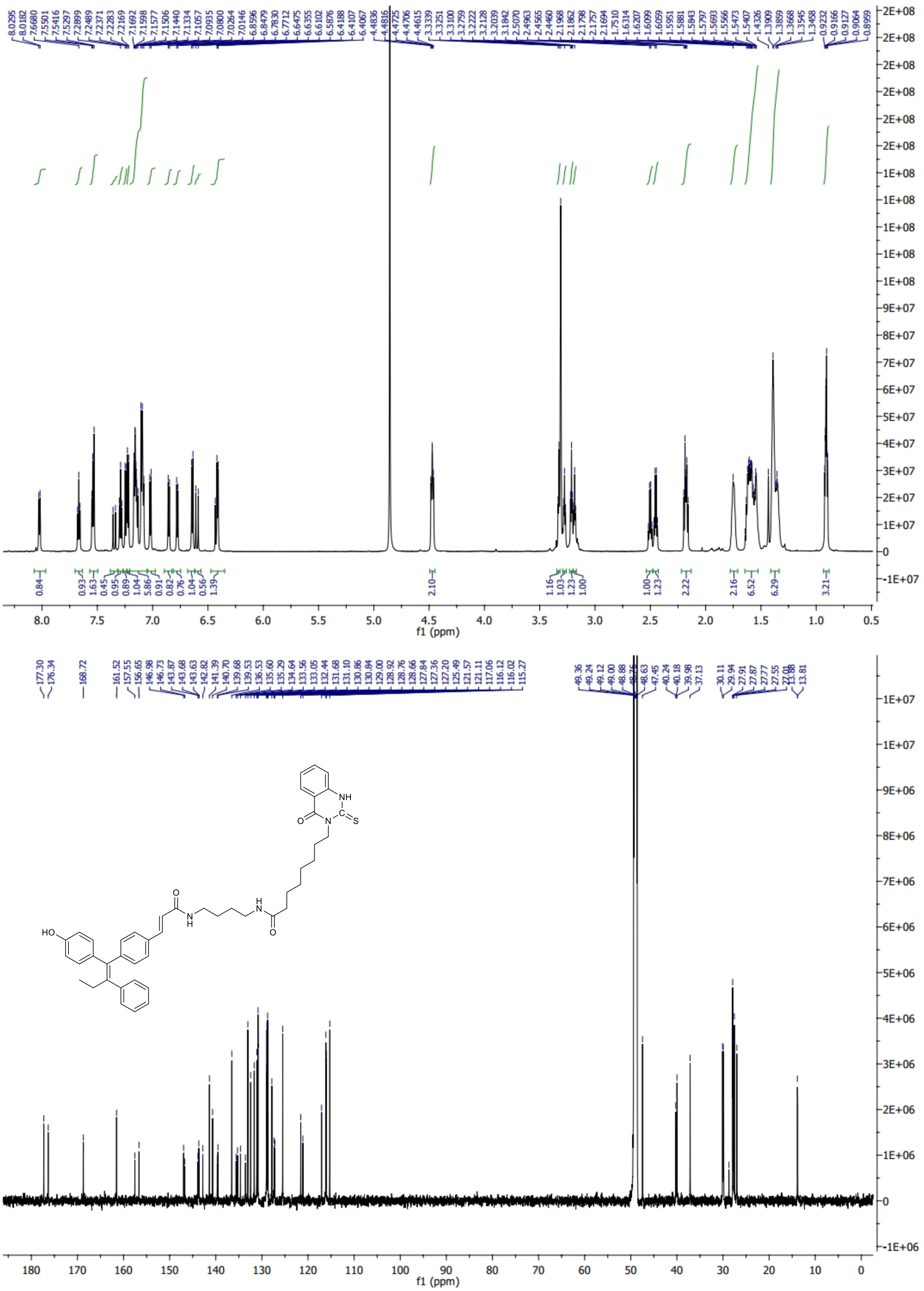

Figure S2: ^{1}H (700 MHz) and ^{13}C NMR (176 MHz) of compound **16** in CD_3OD.

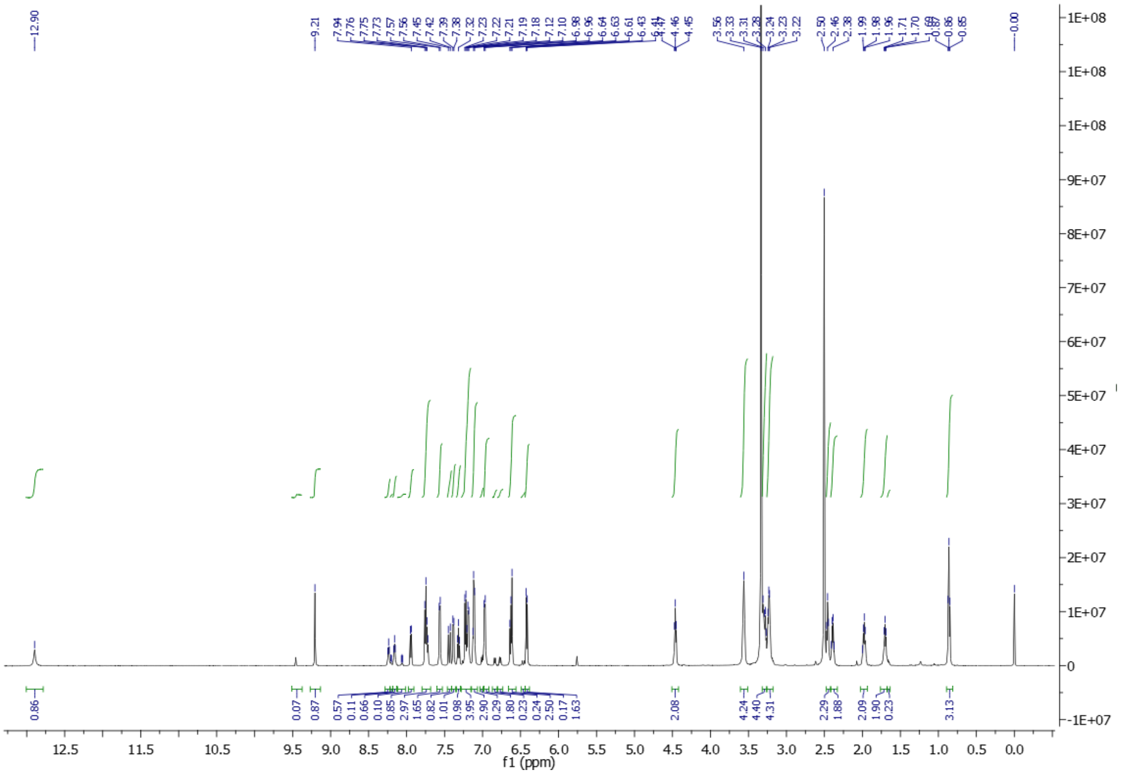

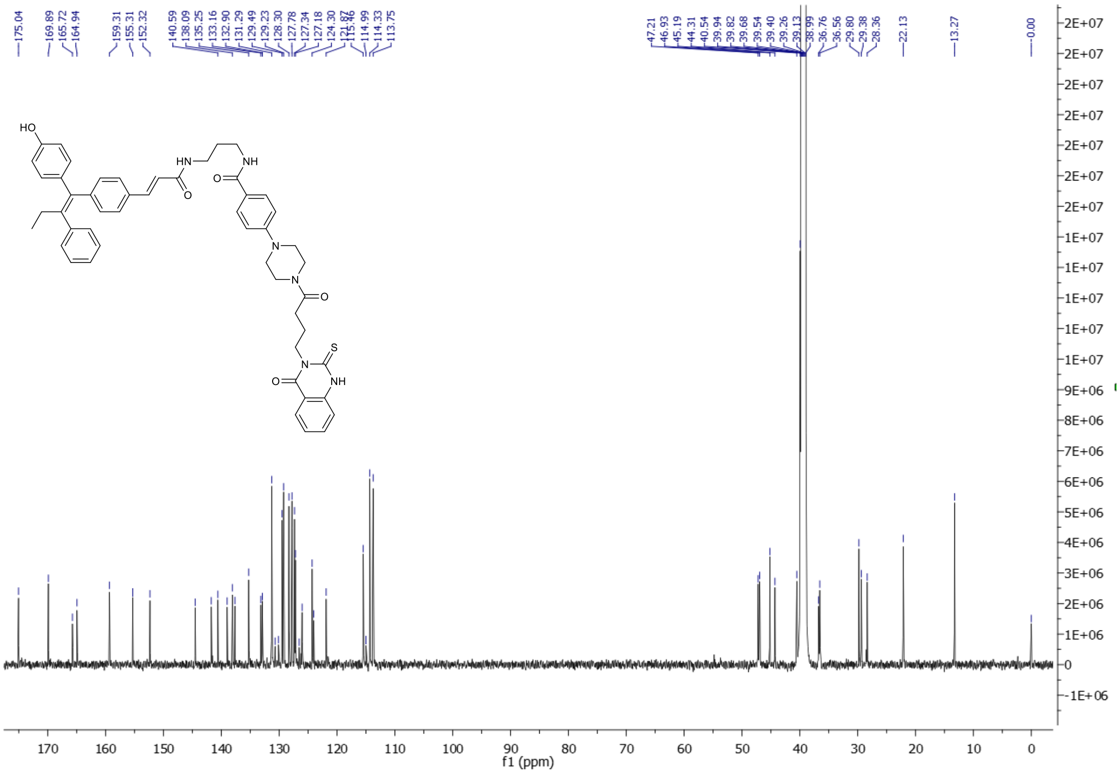

Figure S3: ^{1}H (600 MHz) and ^{13}C NMR (151 MHz) of compound **17** in DMSO-d_6

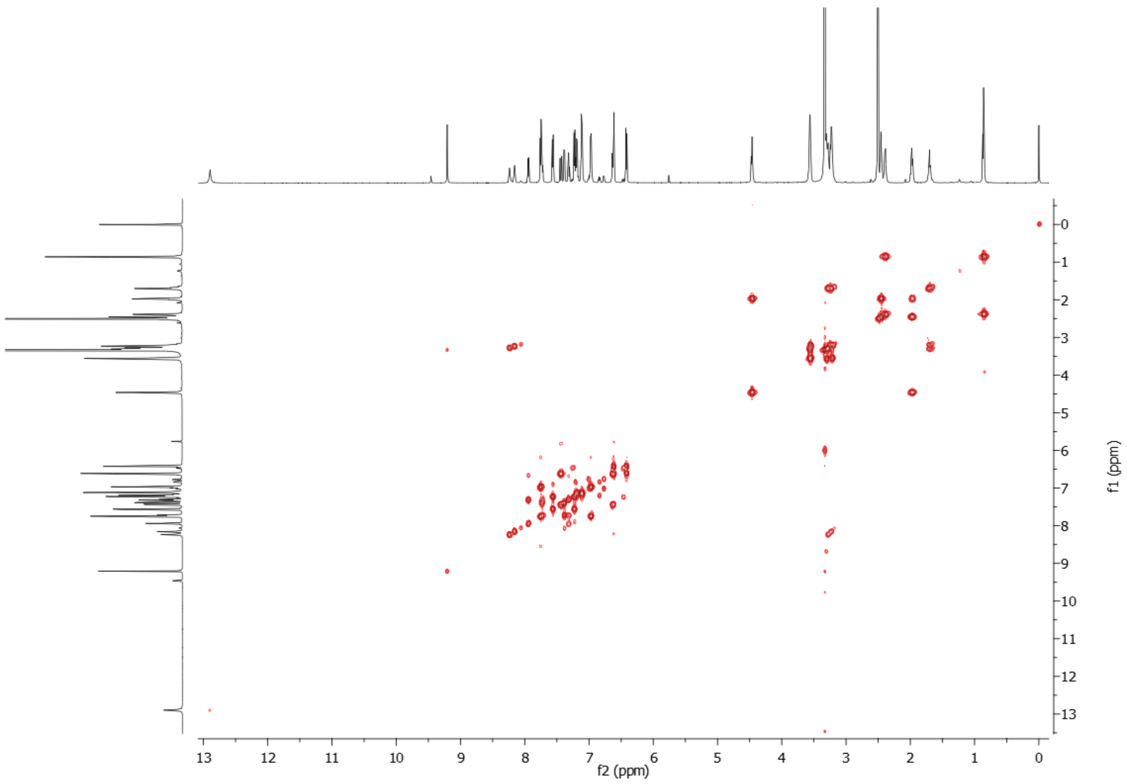

Figure S4: ^{1}H-^{1}H COSY NMR spectrum of compound **17**.

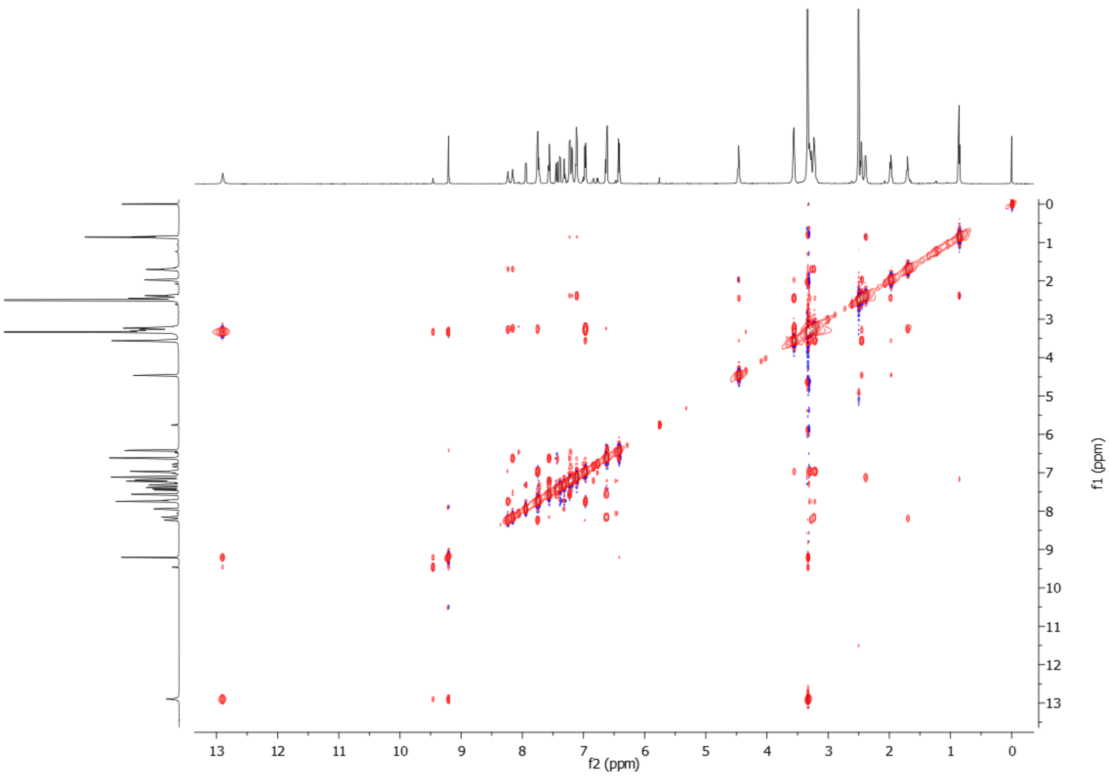

Figure S5: ^{1}H-^{1}H NOESY NMR spectrum of compound **17**.

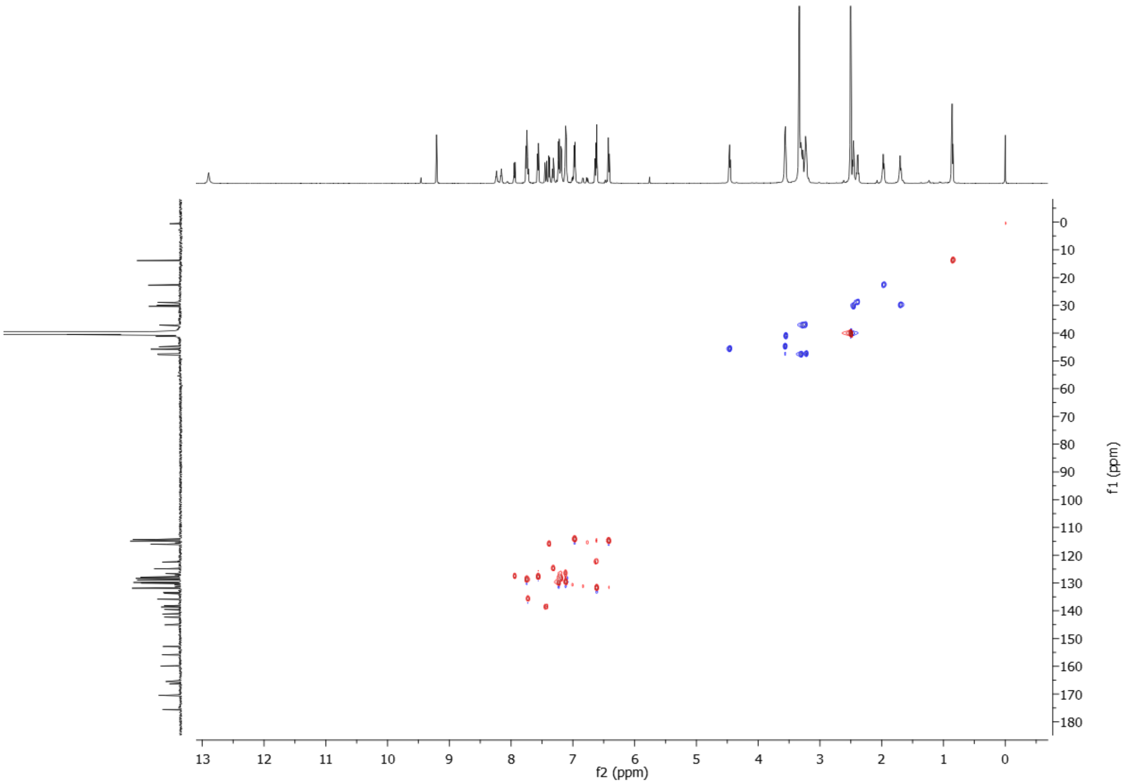

Figure S6: ^1H-^{13}C HSQC NMR spectrum of compound **17**.

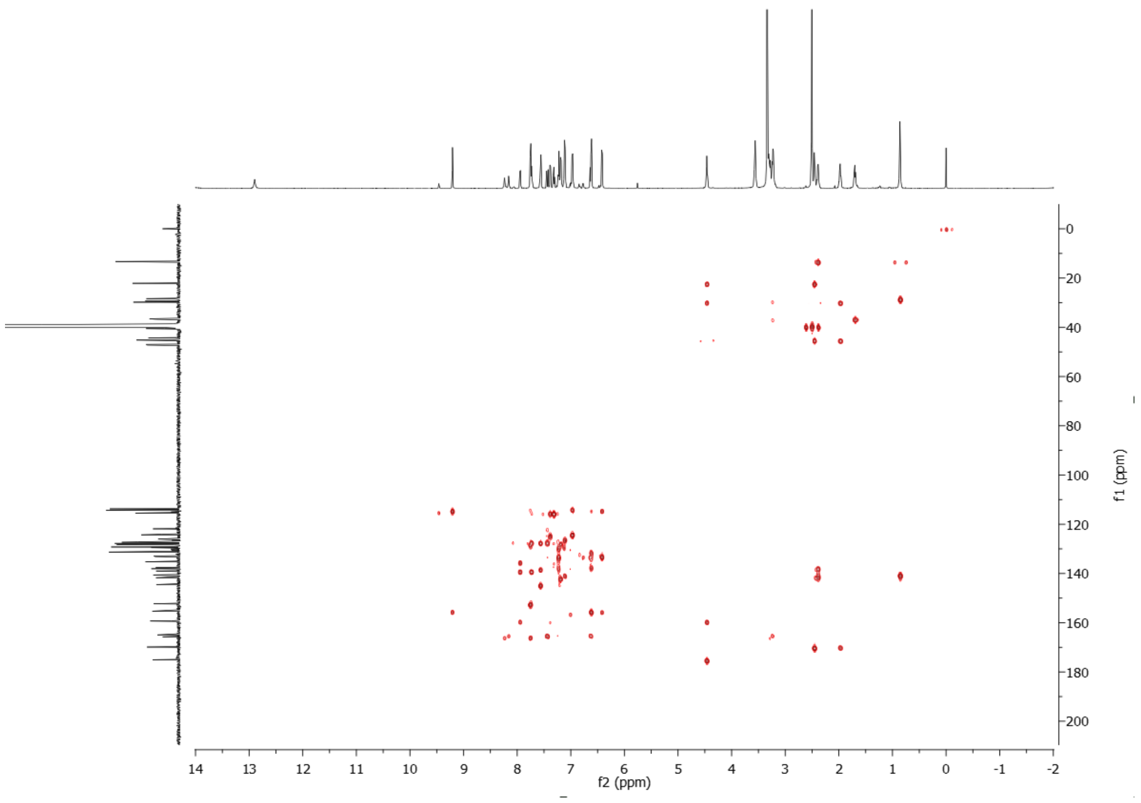

Figure S7: ^{1}H-^{13}C HMBC NMR spectrum of compound **17**.

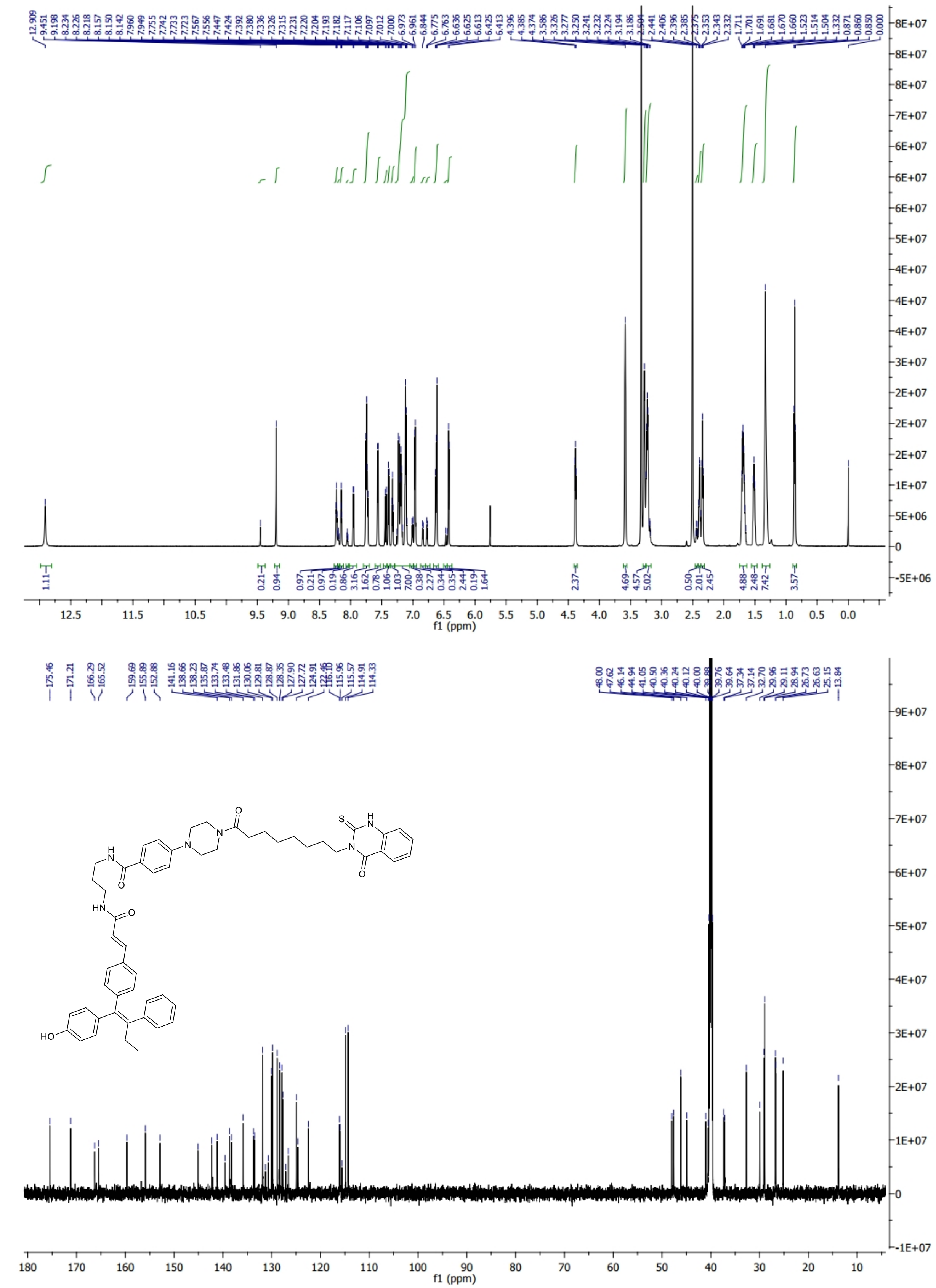

Figure S8: ^{1}H (700 MHz) and ^{13}C NMR (176 MHz) of compound **18** in DMSO-d_6.

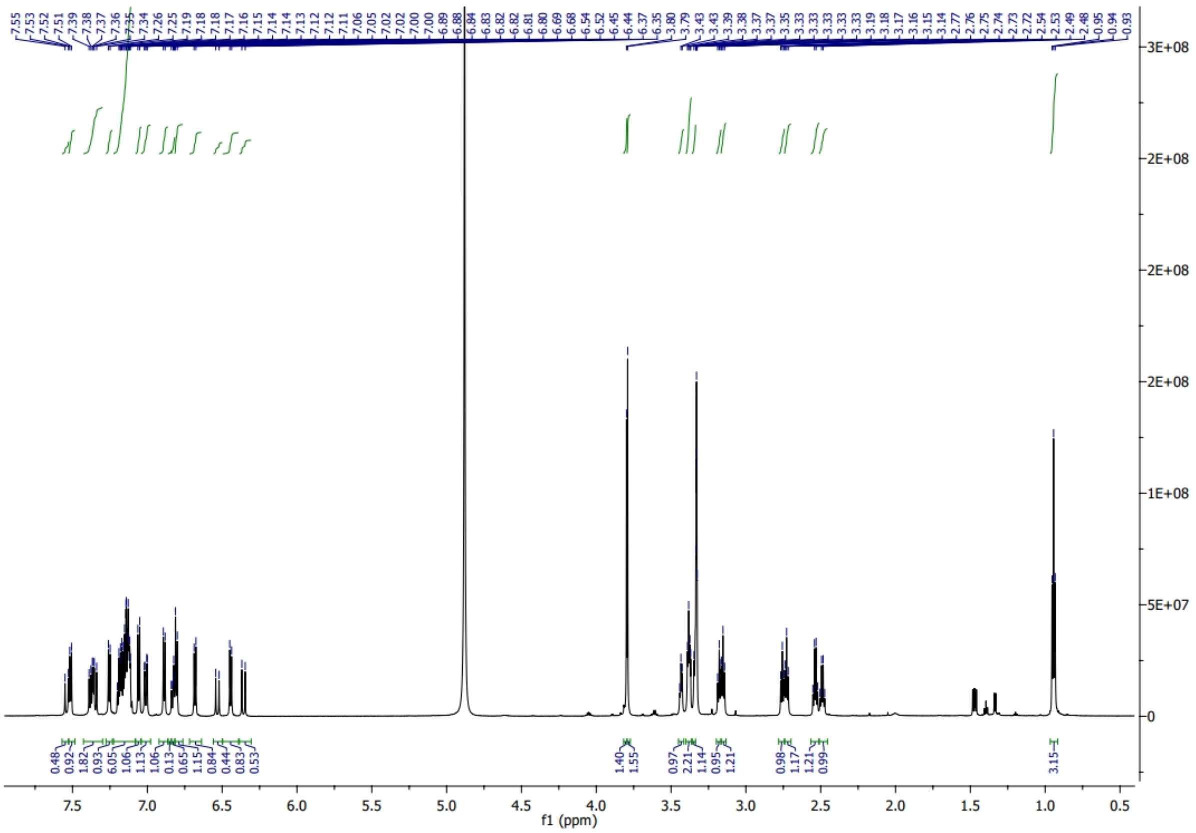

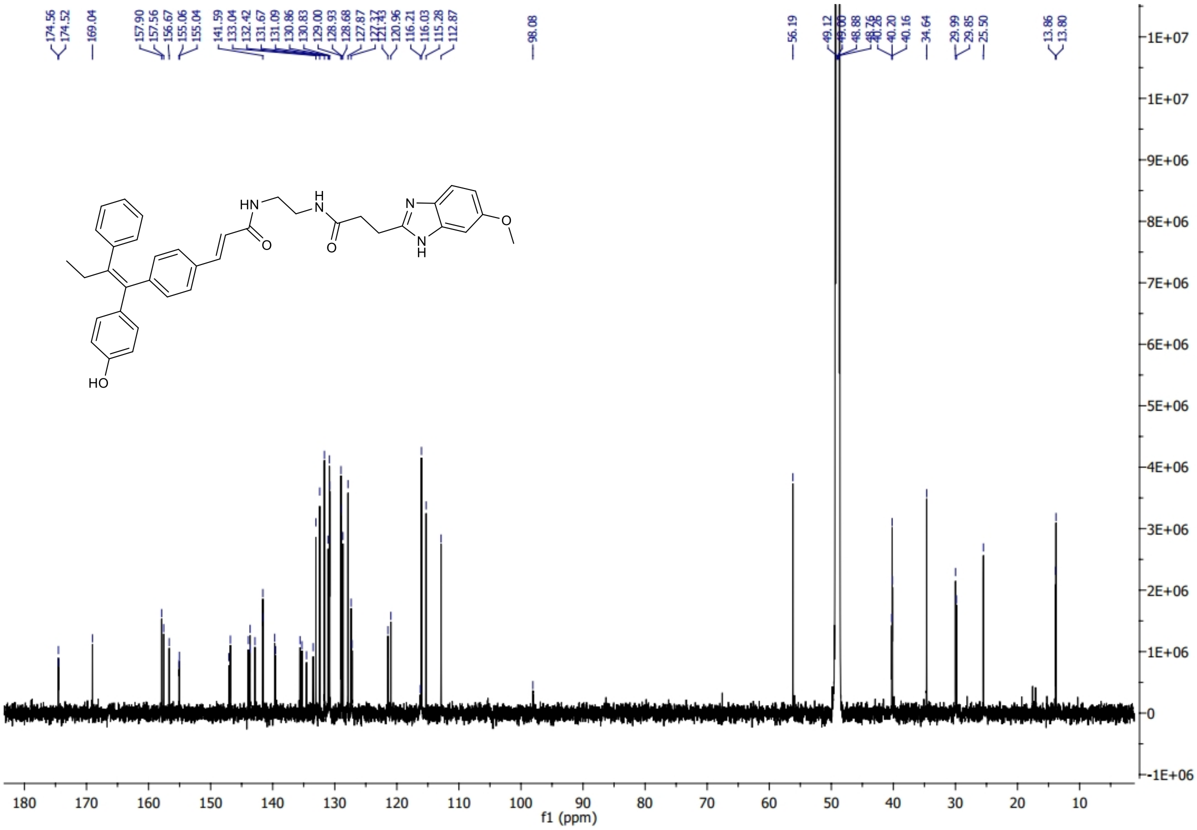

Figure S9: ^{1}H (700 MHz) and ^{13}C NMR (176 MHz) of compound **31** in CD_3OD.

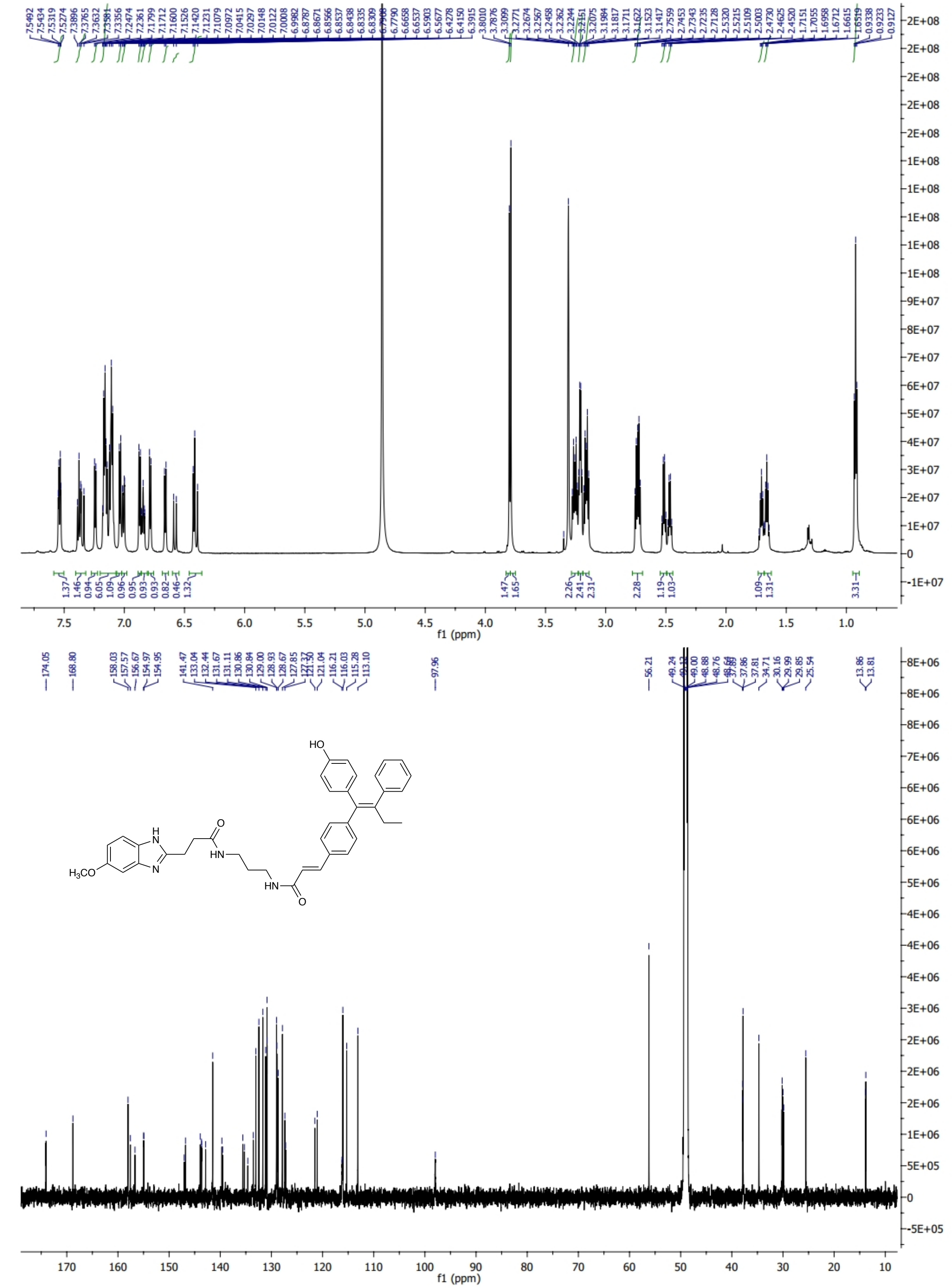

Figure S10: ^{1}H (700 MHz) and ^{13}C NMR (176 MHz) of compound **32** in CD_3OD.

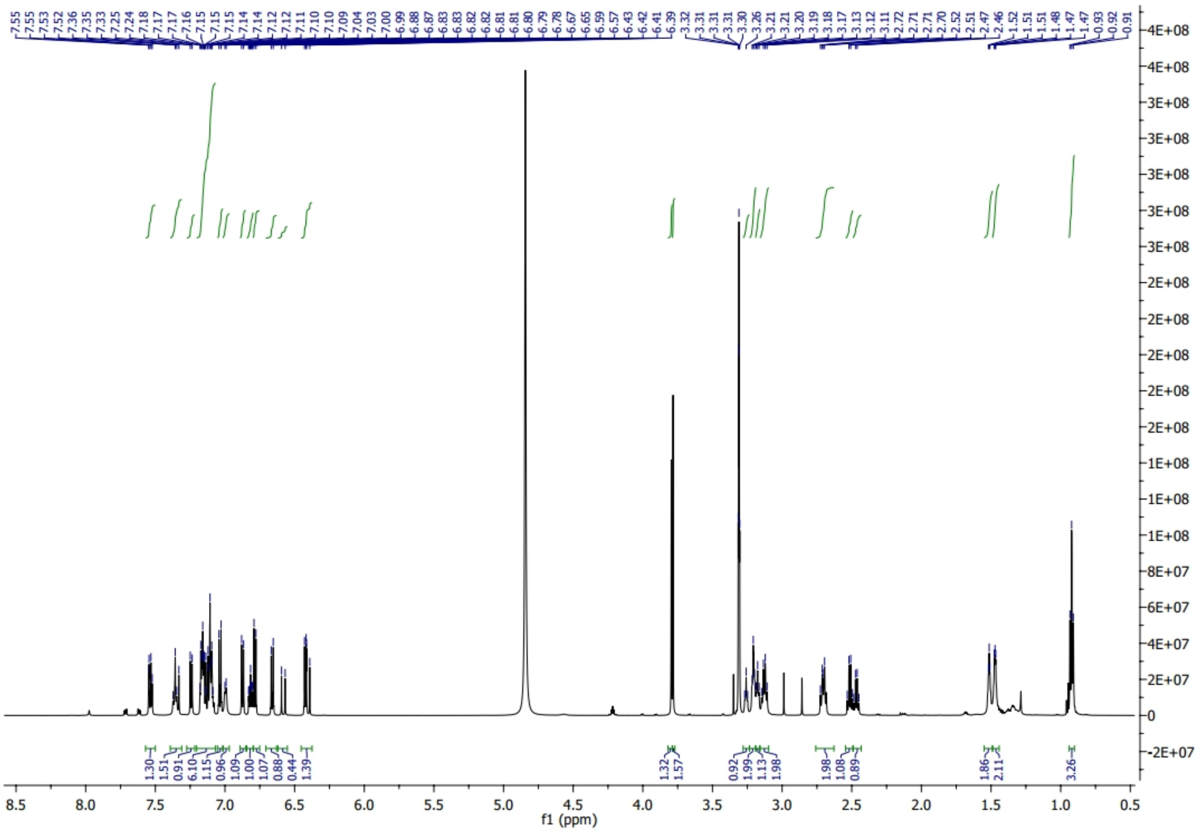

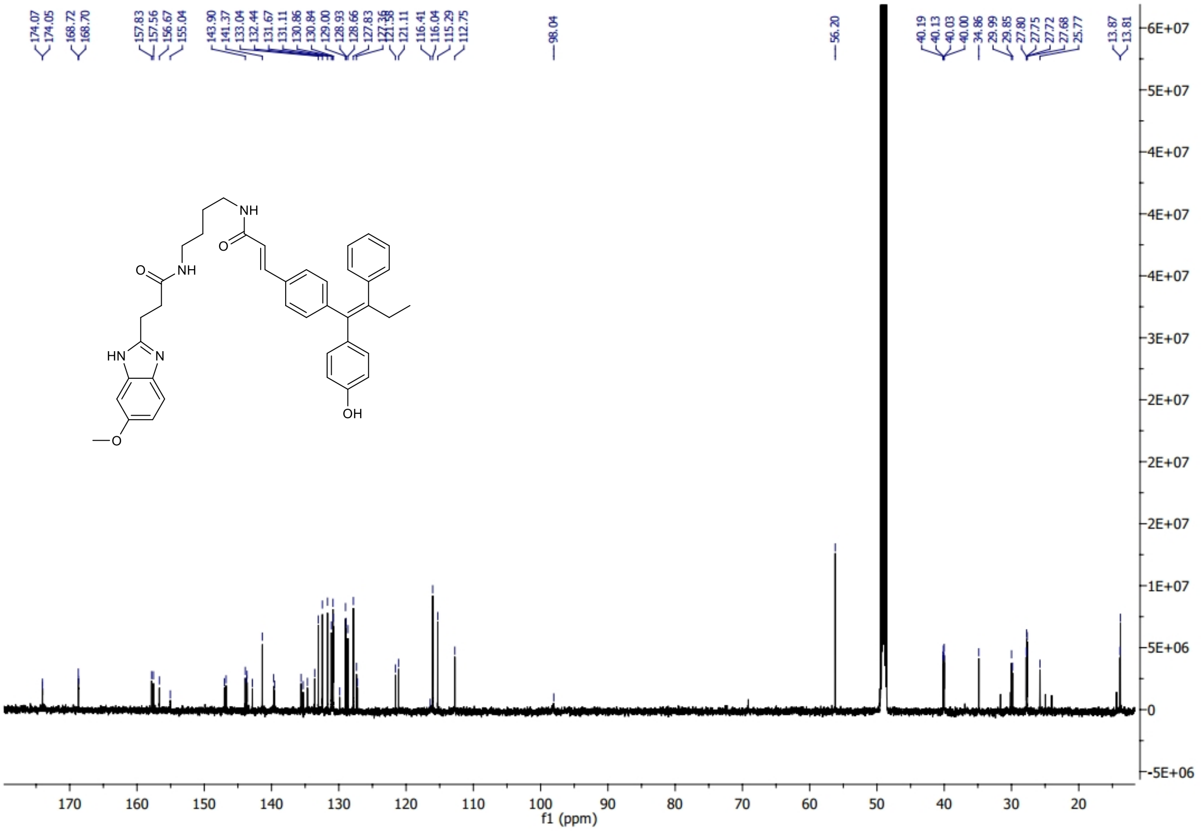

Figure S11: ^{1}H (600 MHz) and ^{13}C NMR (151 MHz) of compound **34** in CD_3OD.

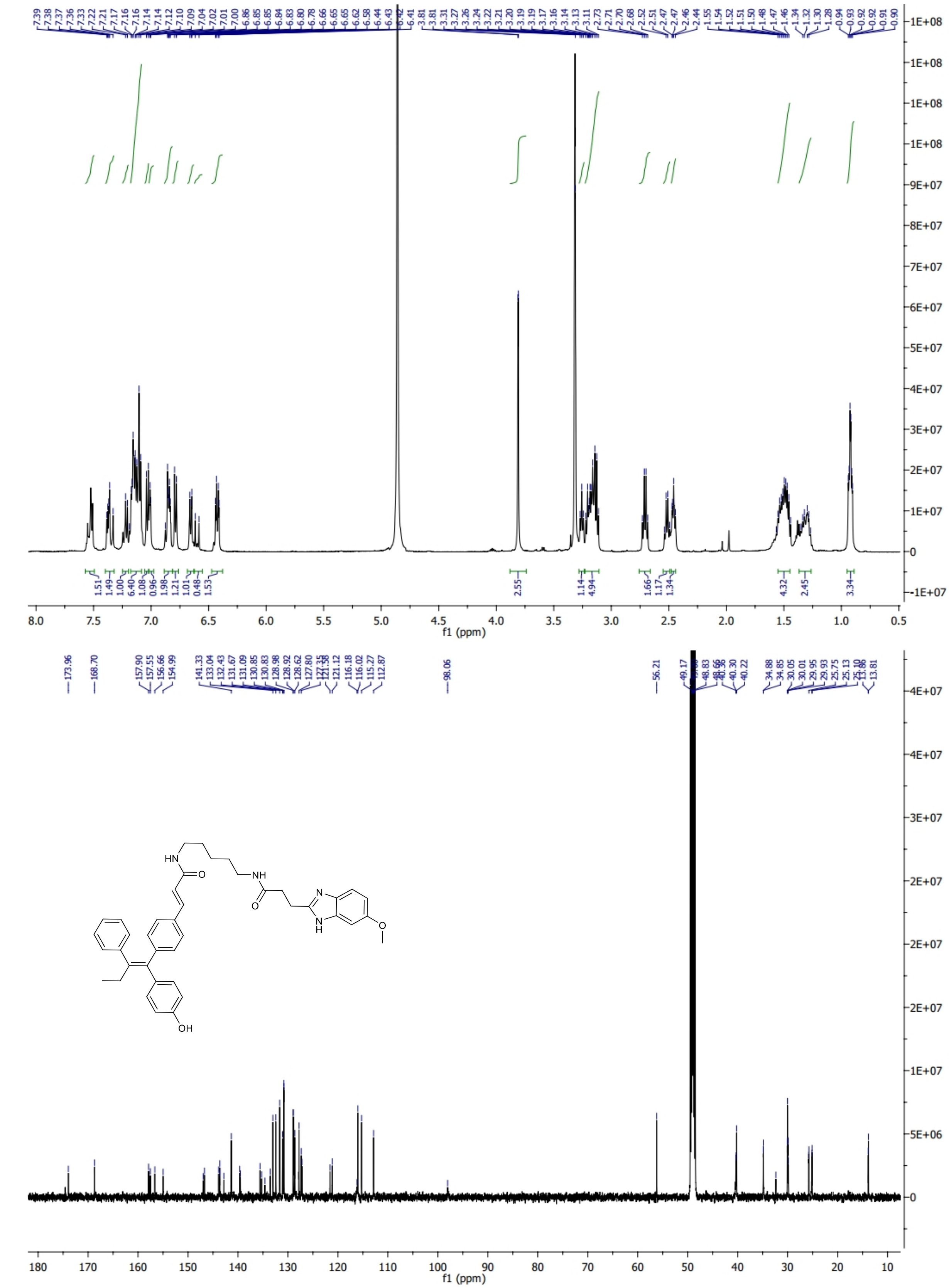

Figure S12: ^{1}H (500 MHz) and ^{13}C NMR (126 MHz) of compound **36** in CD_3OD.

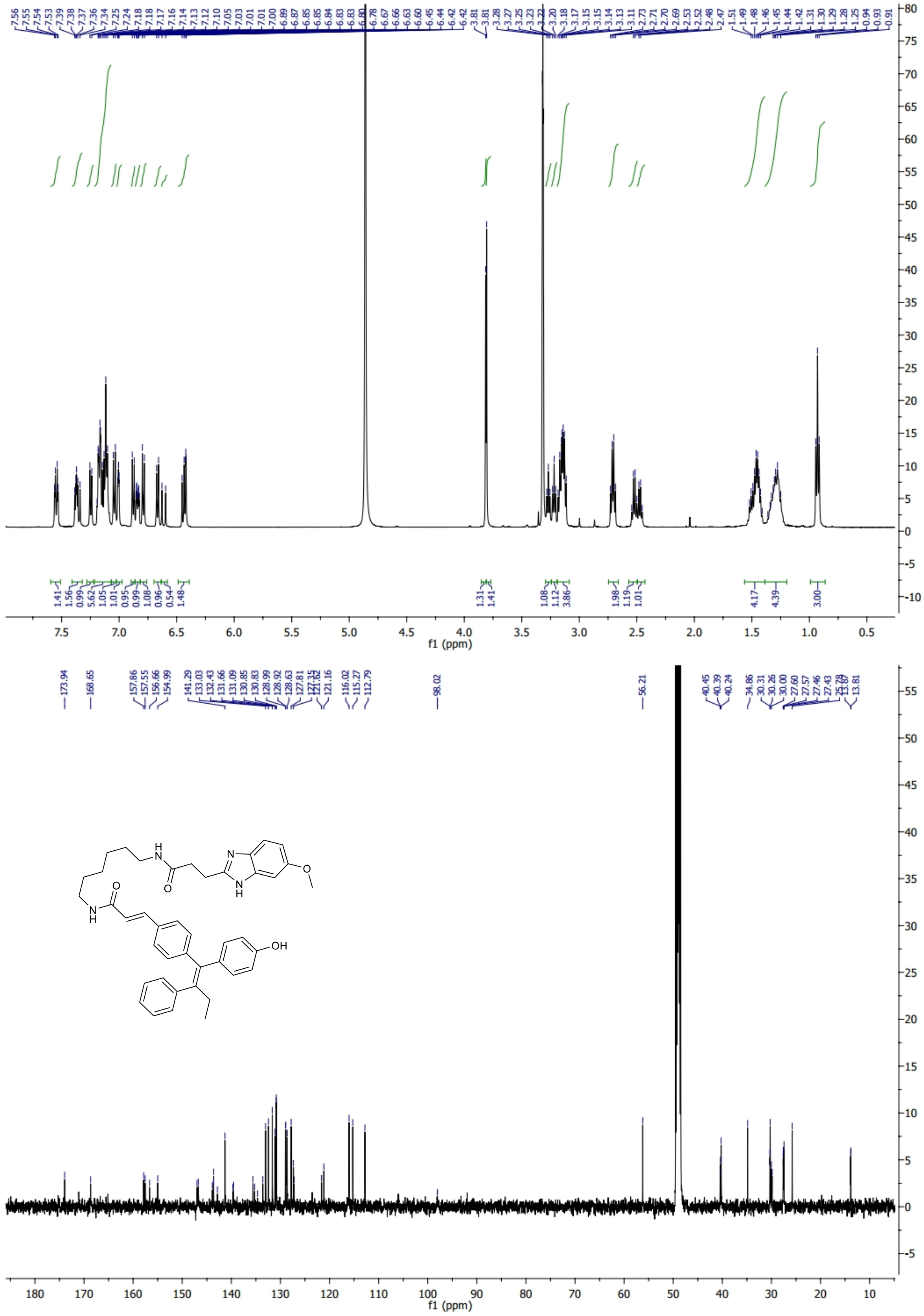

Figure S13: ^{1}H (500 MHz) and ^{13}C NMR (126 MHz) of compound **38** in CD_3OD.

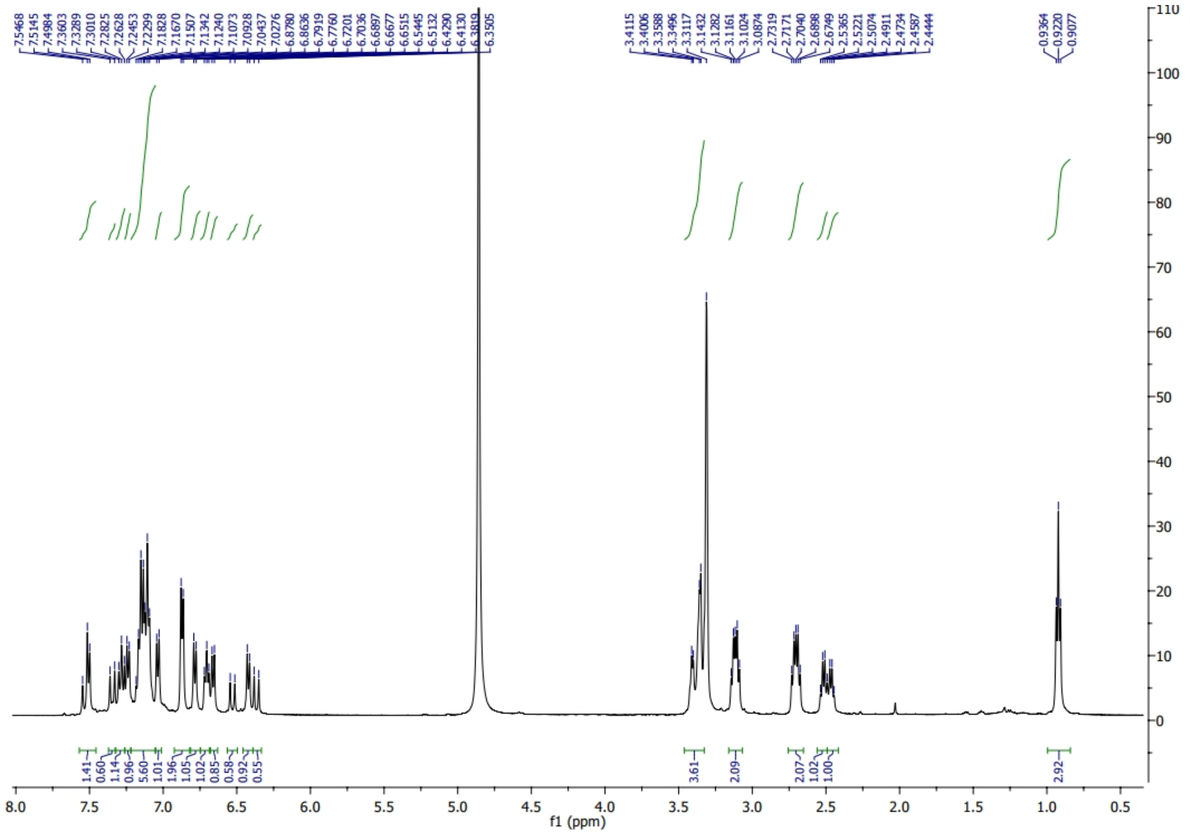

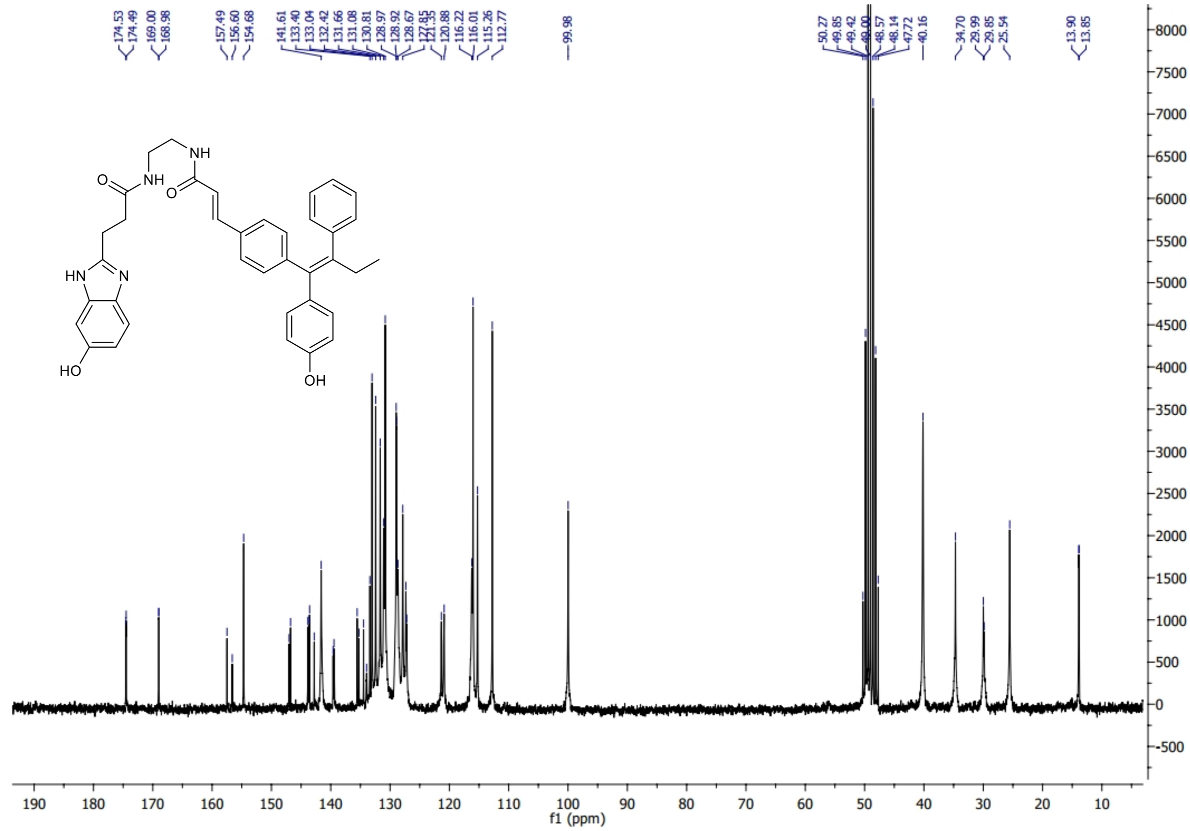

Figure S14: ^{1}H (500 MHz) and ^{13}C NMR (126 MHz) of compound **39** in CD_3OD.

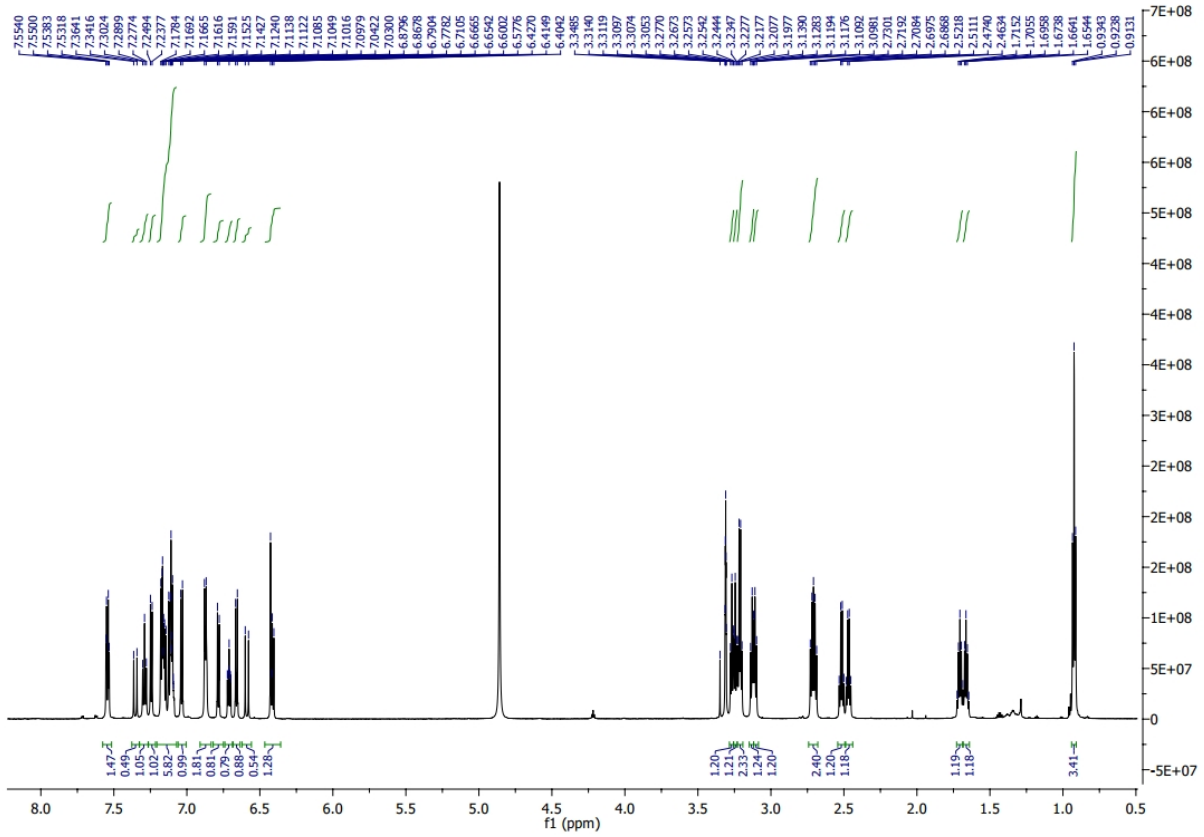

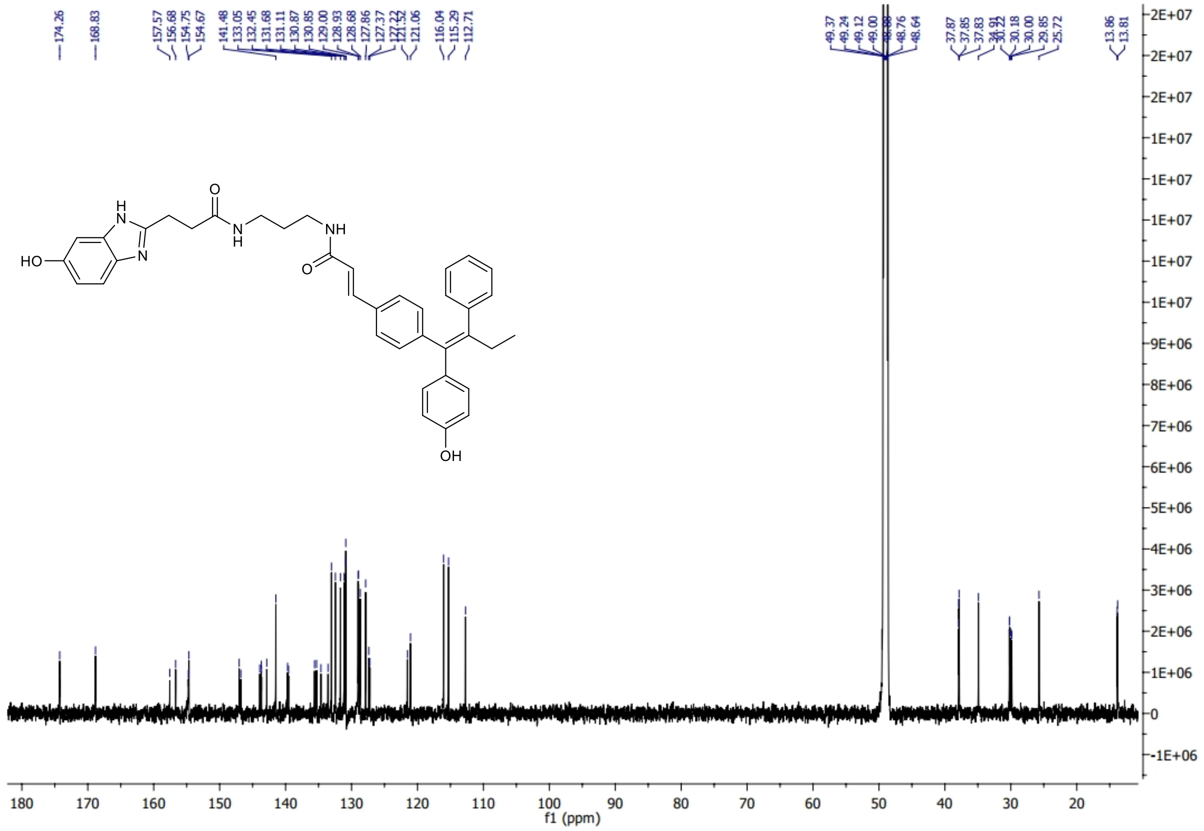

Figure S15: ^{1}H (700 MHz) and ^{13}C NMR (176 MHz) of compound **40** in CD_3OD.

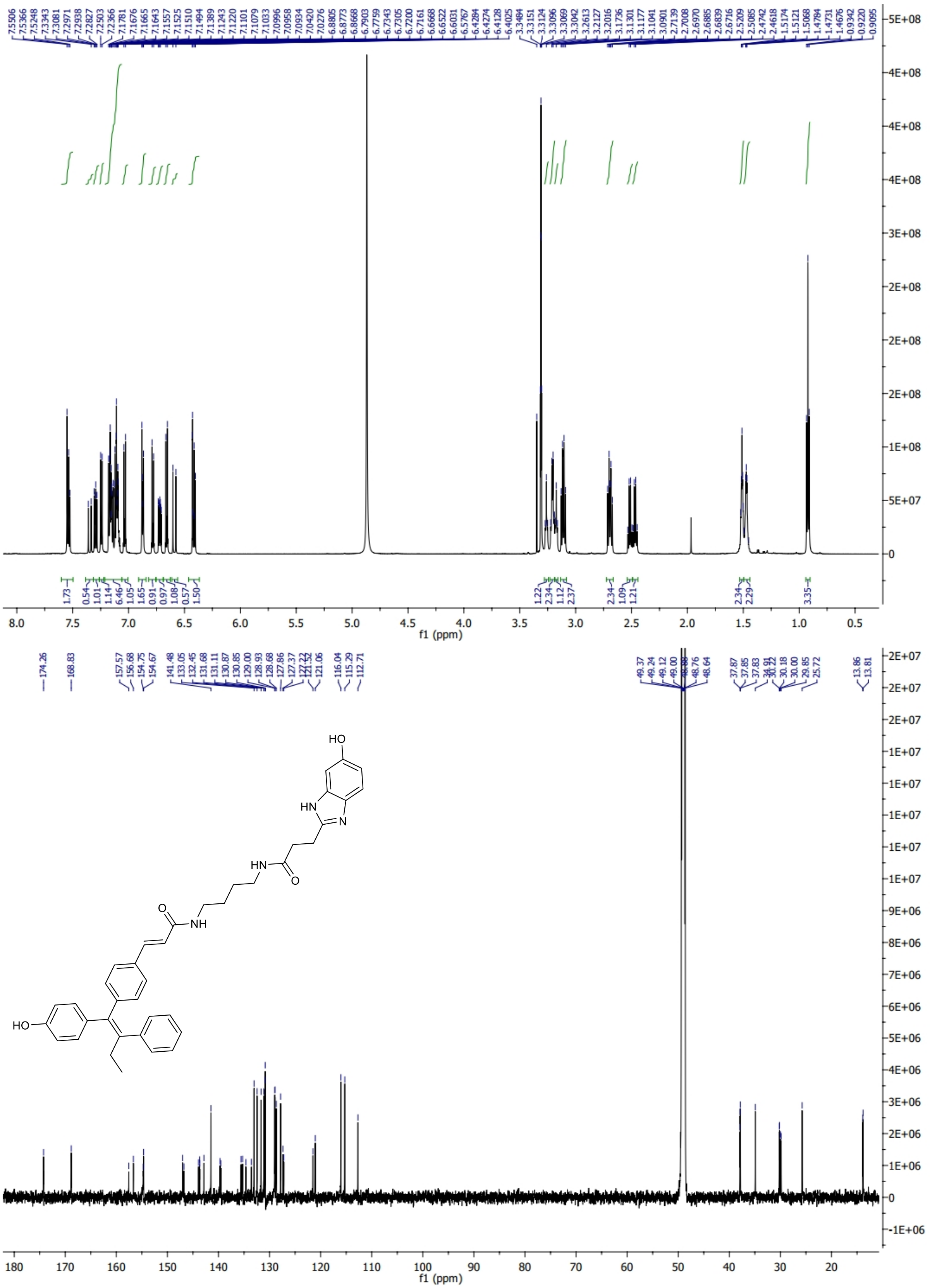

Figure S16: ^{1}H (600 MHz) and ^{13}C NMR (151 MHz) of compound **41** in CD_3OD.

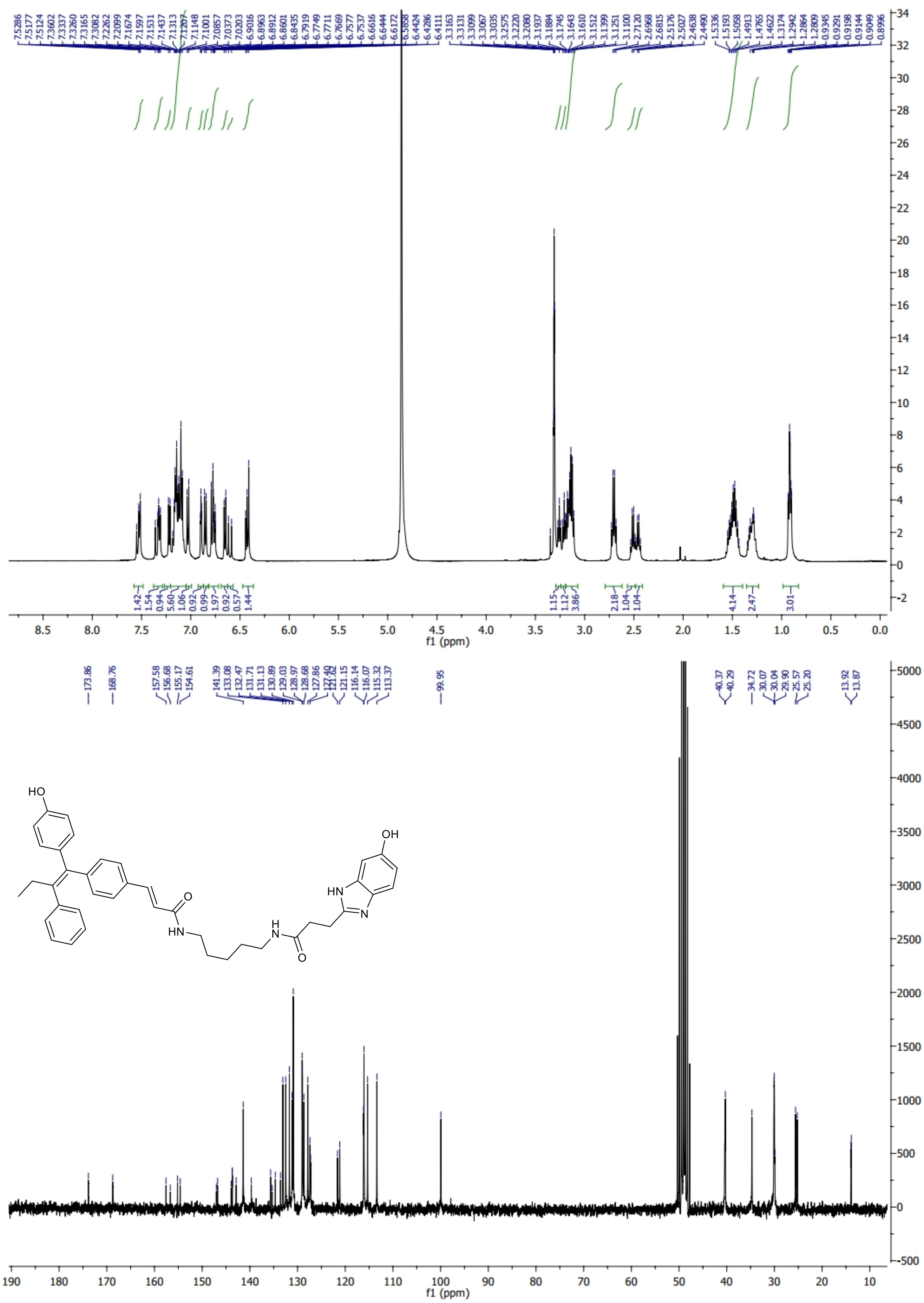

Figure S17: ^{1}H (500 MHz) and ^{13}C NMR (126 MHz) of compound **42** in CD_3OD.

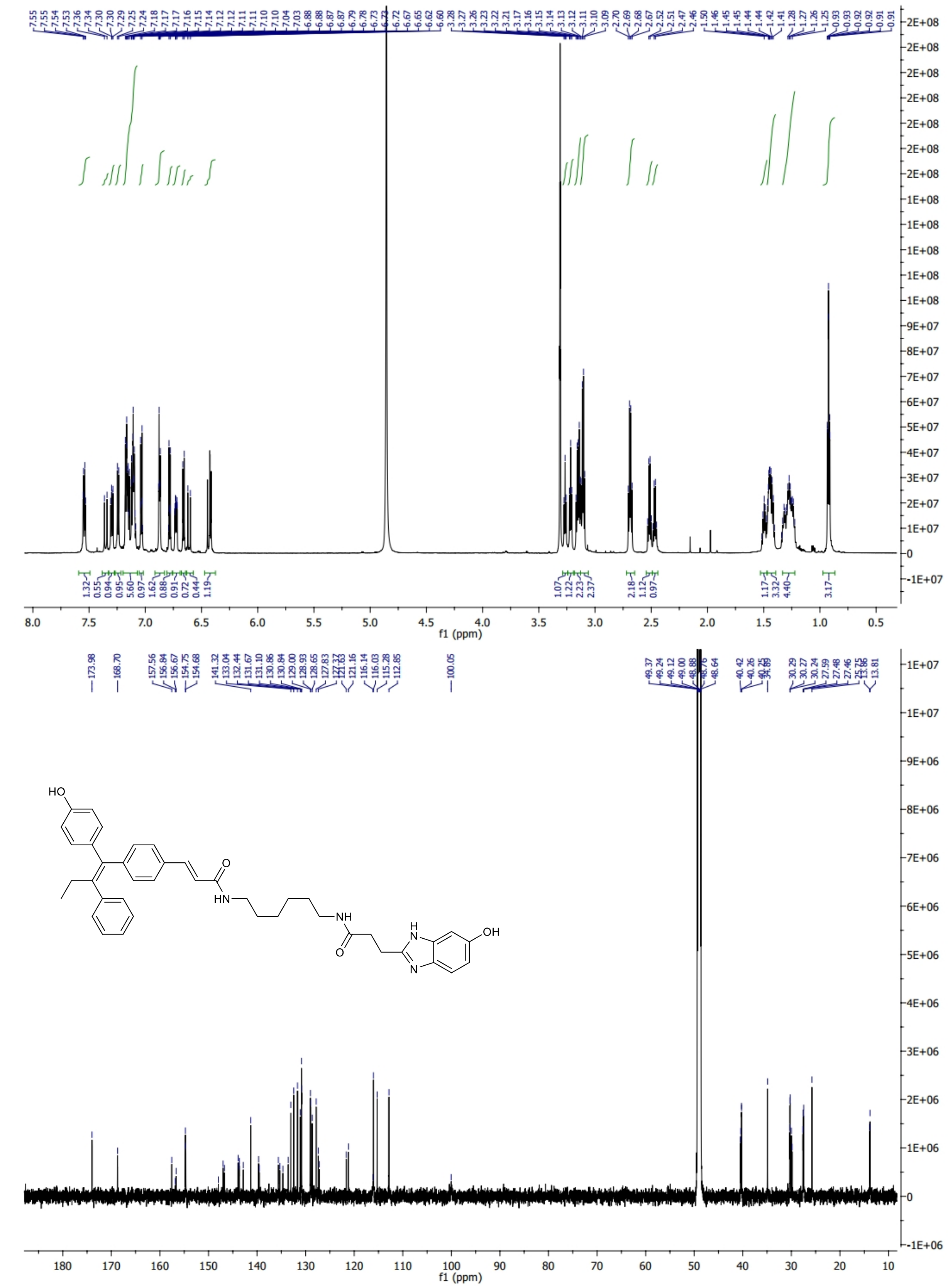

Figure S18: ^{1}H (700 MHz) and ^{13}C NMR (176 MHz) of compound **43** in CD_3OD.

3. HPLC chromatograms of final compounds

HPLC-Methods:

Compounds **15-18**: ACN/water (0.1% TFA) gradient, flow rate: 1.6 mL/min, oven temperature: 30 °C.

Compound **31**: ACN/water (Na_2SO_4, 20 mM (pH 3)) gradient, flow rate: 1.0 mL/min, oven temperature: 30 °C.

Compounds **32**, **34**, **36**, **38**: ACN/water (Na_2SO_4, 20 mM (pH 3)) gradient, flow rate: 1.2 mL/min, oven temperature: 30 °C.

Compound **39**: ACN/water (0.1% TFA) gradient, flow rate: 1.2 mL/min, oven temperature: 30 °C.

Compound **40**: (ACN/MeOH (1:1))/water (Na_2SO_4, 20 mM (pH 3)) gradient, flow rate: 1.0 mL/min, oven temperature: 30 °C.

Compounds **41-43**: (ACN/MeOH (1:1))/water (Na_2SO_4, 20 mM (pH 3)) gradient, flow rate: 1.2 mL/min, oven temperature: 30 °C.

<Chromatogram>

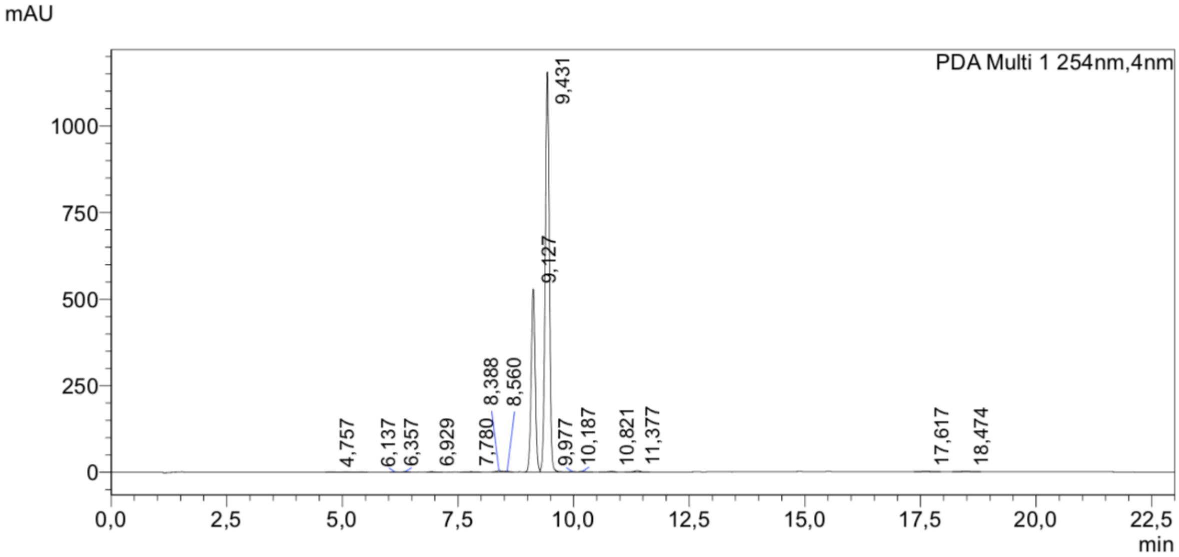

<Peak Table>

PDA Ch1 254nm

Peak#	Ret. Time	Area	Height	Conc.	Unit	Area%
1	4,757	18584	1201	0,000		0,177
2	6,137	4111	653	0,000		0,039
3	6,357	9440	1480	0,000		0,090
4	6,929	8242	1290	0,000		0,079
5	7,780	7927	1000	0,000		0,076
6	8,388	38136	3865	0,000		0,364
7	8,560	10851	1829	0,000		0,103
8	9,127	3225421	527900	0,000		30,755
9	9,431	7072161	1152985	0,000		67,435
10	9,977	10181	1387	0,000		0,097
11	10,187	13168	1788	0,000		0,126
12	10,821	15242	1778	0,000		0,145
13	11,377	28749	3652	0,000		0,274
14	17,617	11062	801	0,000		0,105
15	18,474	14123	873	0,000		0,135
Total		10487400	1702480			100,000

Figure S19: HPLC chromatogram of compound **15**.

<Chromatogram>

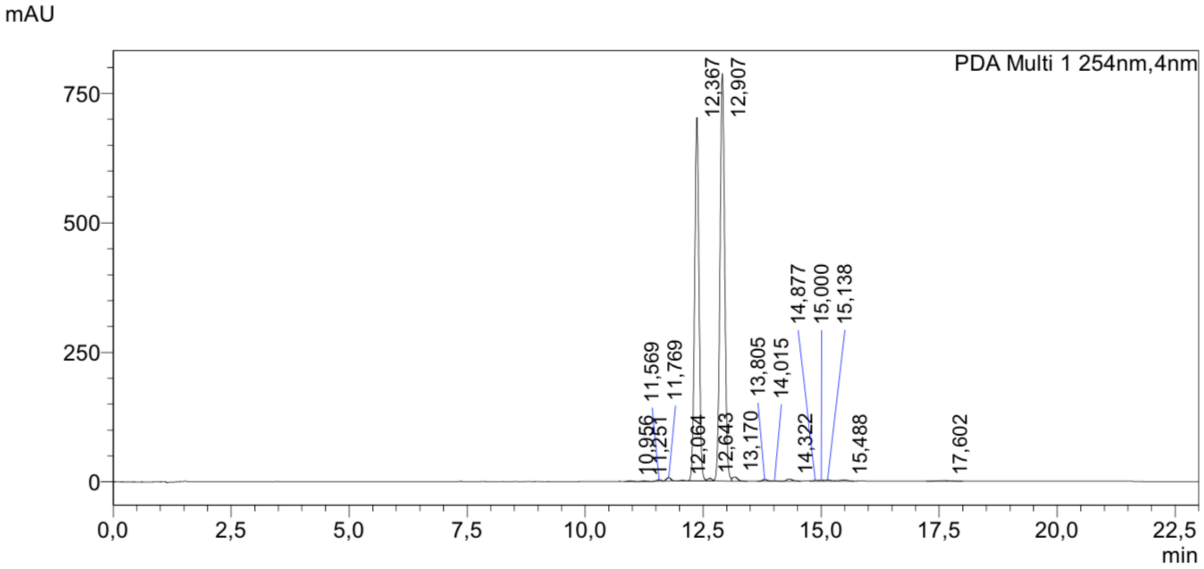

<Peak Table>

PDA Ch1 254nm

Peak#	Ret. Time	Area	Height	Conc.	Unit	Area%
1	10,956	7633	1076	0,000		0,073
2	11,251	4526	758	0,000		0,043
3	11,569	16477	2707	0,000		0,158
4	11,769	43060	6772	0,000		0,413
5	12,064	8570	1293	0,000		0,082
6	12,367	4561344	701240	0,000		43,769
7	12,643	33141	5152	0,000		0,318
8	12,907	5517245	784681	0,000		52,941
9	13,170	62713	8241	0,000		0,602
10	13,805	27100	3572	0,000		0,260
11	14,015	7822	928	0,000		0,075
12	14,322	37835	4310	0,000		0,363
13	14,877	11037	1529	0,000		0,106
14	15,000	8499	1421	0,000		0,082
15	15,138	21275	2089	0,000		0,204
16	15,488	30217	2259	0,000		0,290
17	17,602	22921	1078	0,000		0,220
Total		10421416	1529106			100,000

Figure S20: HPLC chromatogram of compound **16**.

<Chromatogram>

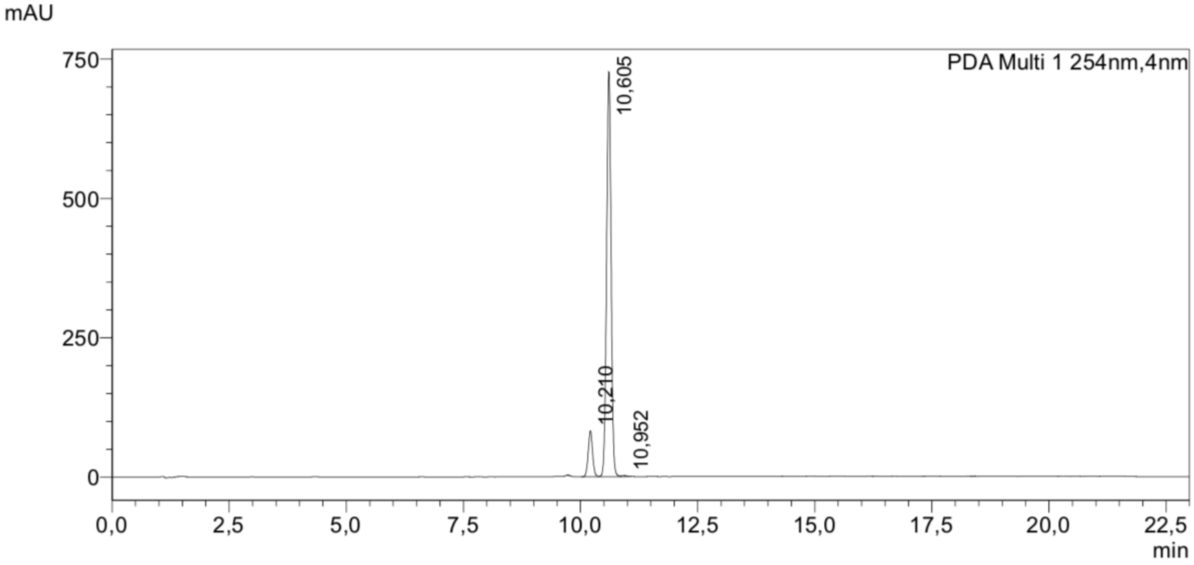

<Peak Table>

PDA Ch1 254nm

Peak#	Ret. Time	Area	Height	Conc.	Unit	Area%
1	10,210	536504	81730	0,000		9,881
2	10,605	4879566	725645	0,000		89,871
3	10,952	13465	1599	0,000		0,248
Total		5429535	808974			100,000

Figure S21: HPLC chromatogram of compound **17**.

<Chromatogram>

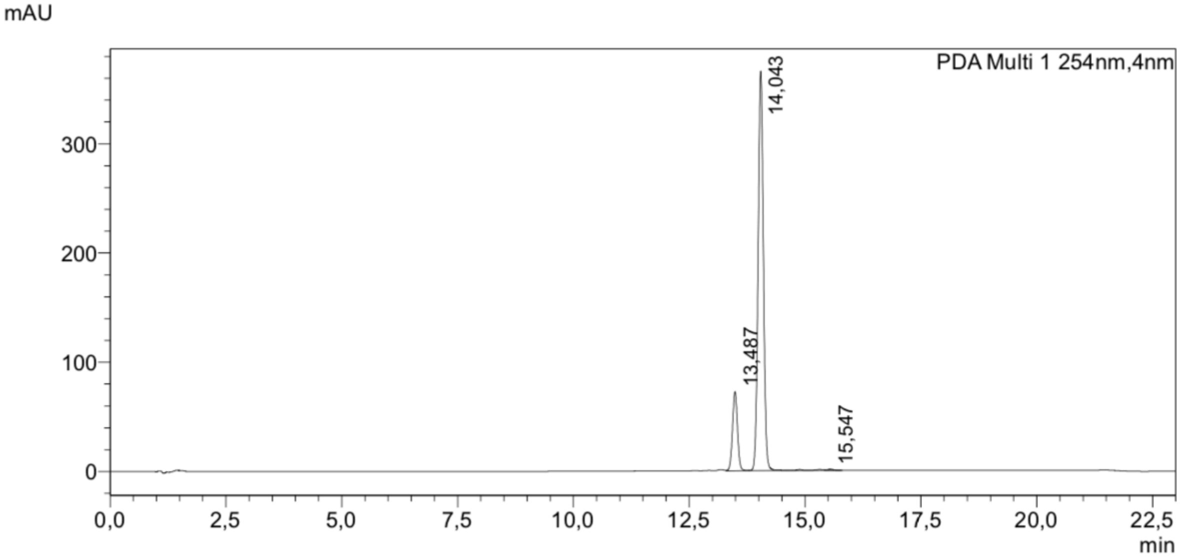

<Peak Table>

PDA Ch1 254nm

Peak#	Ret. Time	Area	Height	Conc.	Unit	Area%
1	13,487	540677	72203	0,000		15,611
2	14,043	2911616	364654	0,000		84,069
3	15,547	11053	1094	0,000		0,319
Total		3463346	437951			100,000

Figure S22: HPLC chromatogram of compound **18**.

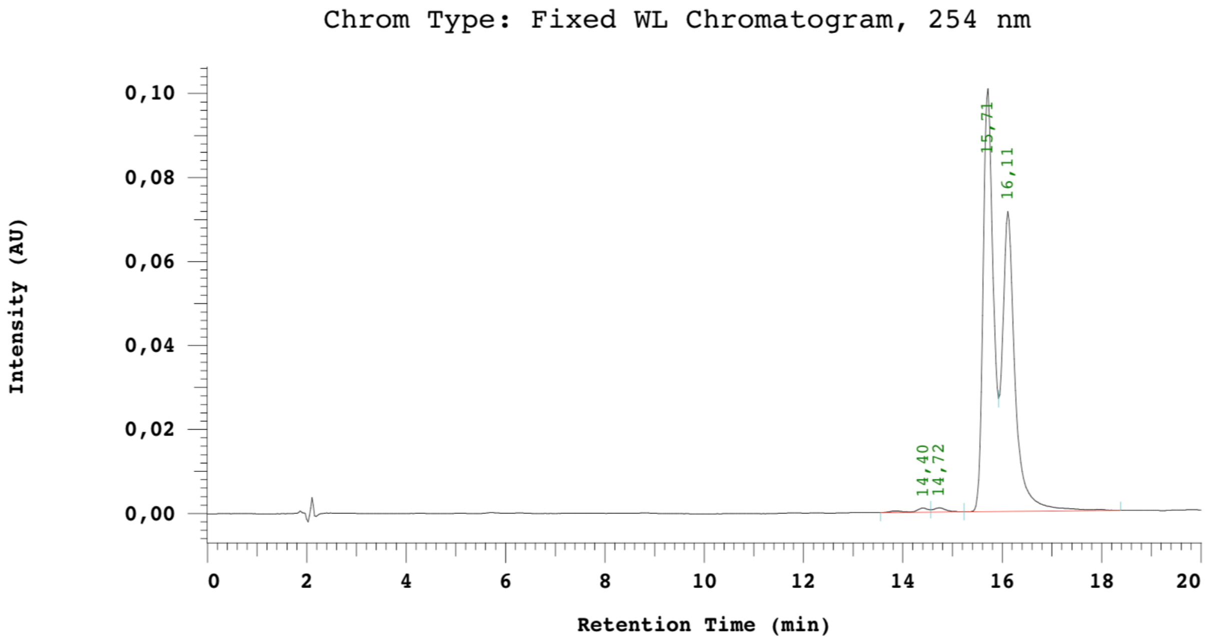

No.	RT	Area	Conc 1
1	14,40	11023	0,798
2	14,72	9381	0,679
3	15,71	694343	50,243
4	16,11	667228	48,281
		1381975	100,000

Figure S23: HPLC chromatogram of compound **31**.

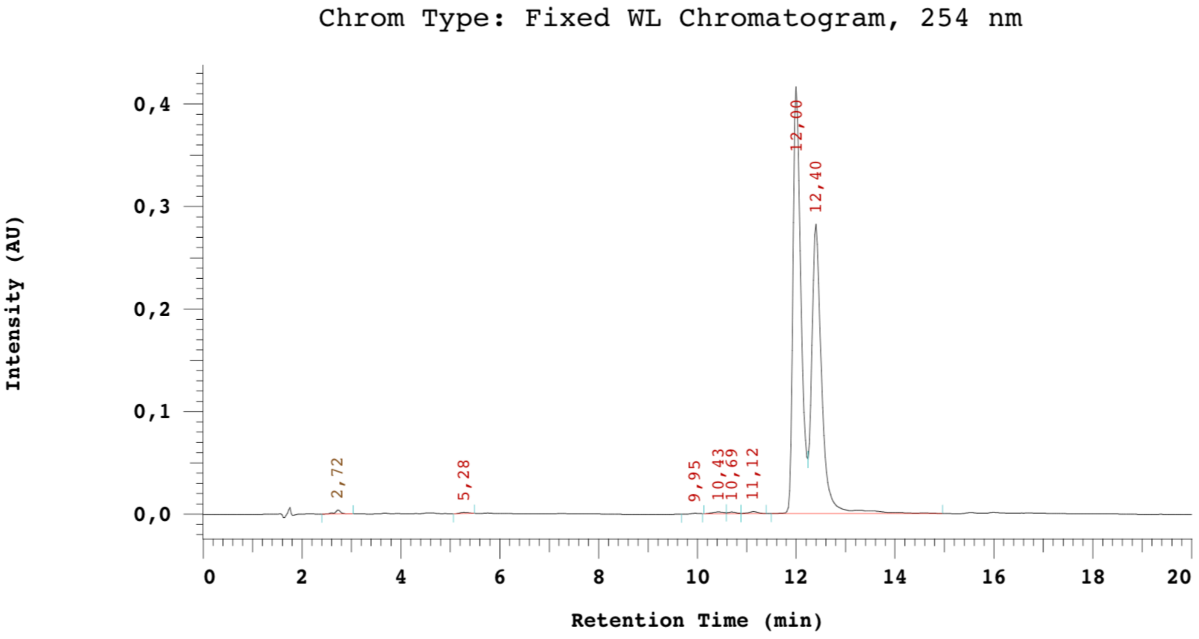

No.	RT	Area	Conc 1	BC
1	2,72	16793	0,387	MC
2	5,28	6900	0,159	MC
3	9,95	0	0,000	
4	10,43	12946	0,298	MC
5	10,69	8469	0,195	MC
6	11,12	11620	0,268	MC
7	12,00	2288033	52,681	MC
8	12,40	1998415	46,013	MC
		4343176	100,000	

Figure S24: HPLC chromatogram of compound **32**.

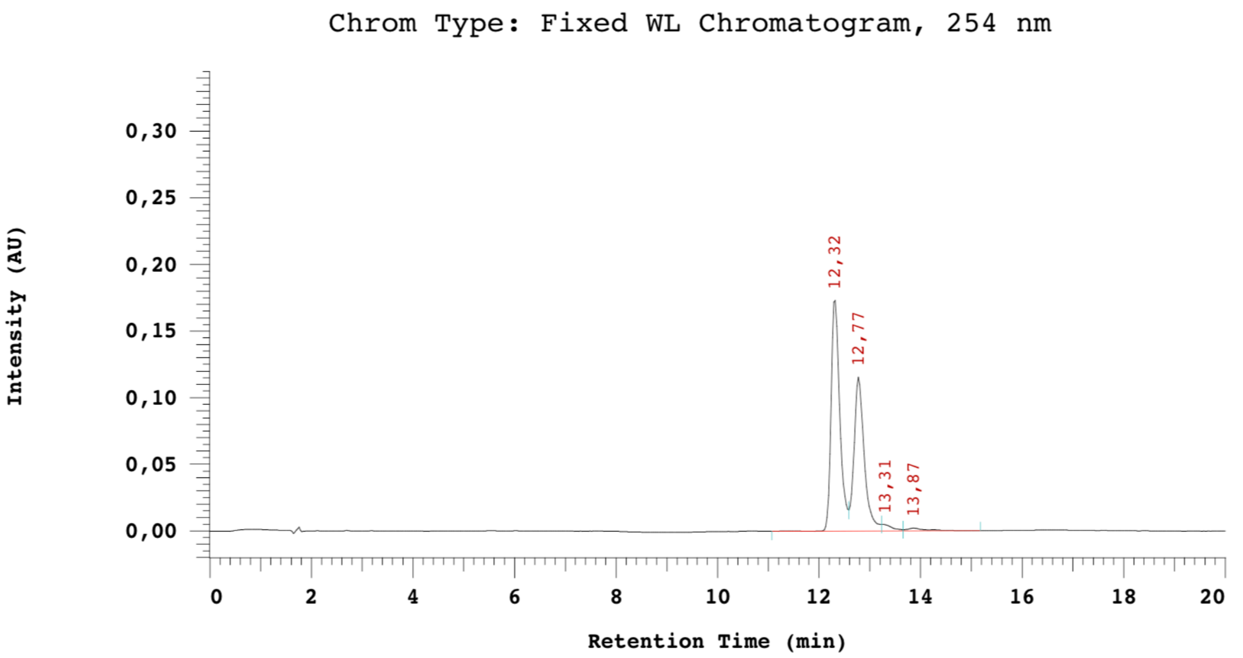

No.	RT	Area	Conc 1	BC
1	12,32	1027105	53,835	MC
2	12,77	811802	42,550	MC
3	13,31	37617	1,972	MC
4	13,87	31345	1,643	MC
		1907869	100,000	

Figure S25: HPLC chromatogram of compound **34**.

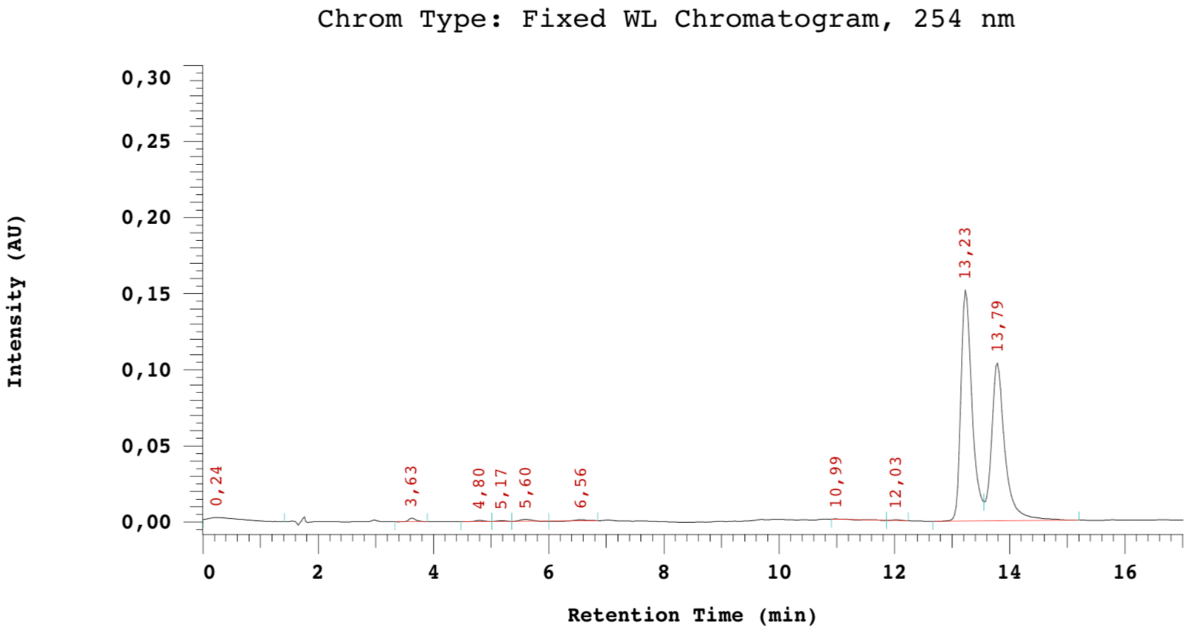

No.	RT	Area	Conc 1	BC
1	0,24	0	0,000	
2	3,63	9623	0,507	MC
3	4,80	3875	0,204	MC
4	5,17	2187	0,115	MC
5	5,60	6664	0,351	MC
6	6,56	2079	0,109	MC
7	10,99	2420	0,127	MC
8	12,03	2517	0,133	MC
9	13,23	1006054	52,967	MC
10	13,79	863974	45,487	MC
		1899393	100,000	

Figure S26: HPLC chromatogram of compound **36**.

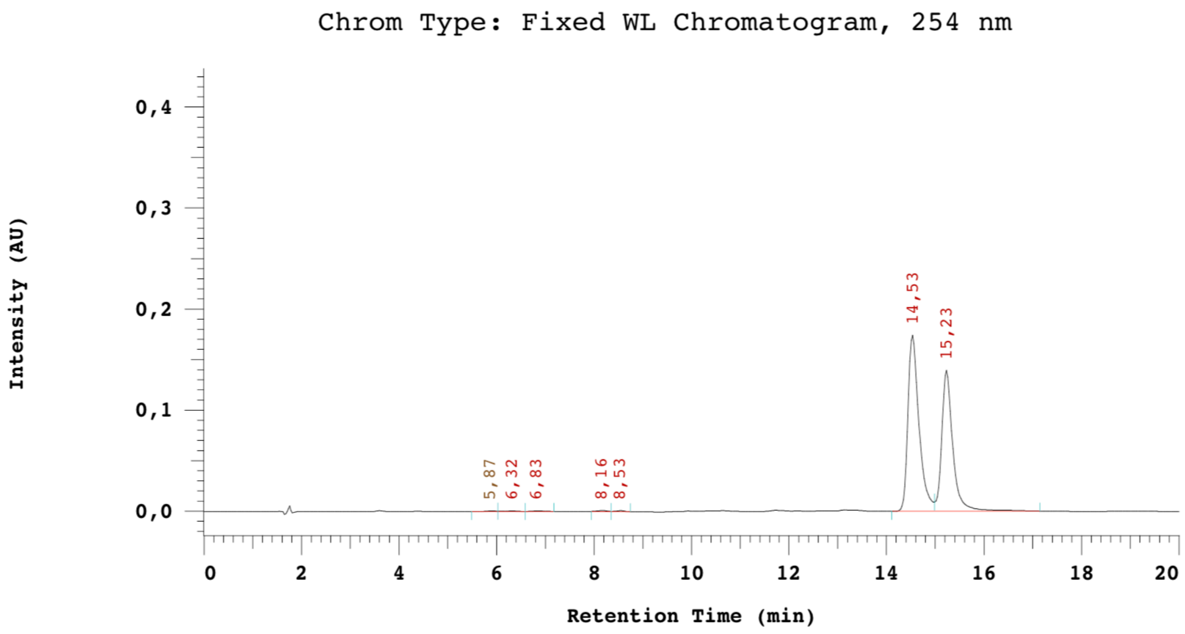

No.	RT	Area	Conc 1	BC
1	5,87	3418	0,137	MC
2	6,32	4013	0,161	MC
3	6,83	4413	0,177	MC
4	8,16	6036	0,242	MC
5	8,53	6043	0,242	MC
6	14,53	1368410	54,764	MC
7	15,23	1106416	44,279	MC
		2498749	100,000	

Figure S27: HPLC chromatogram of compound **38**.

<Chromatogram>

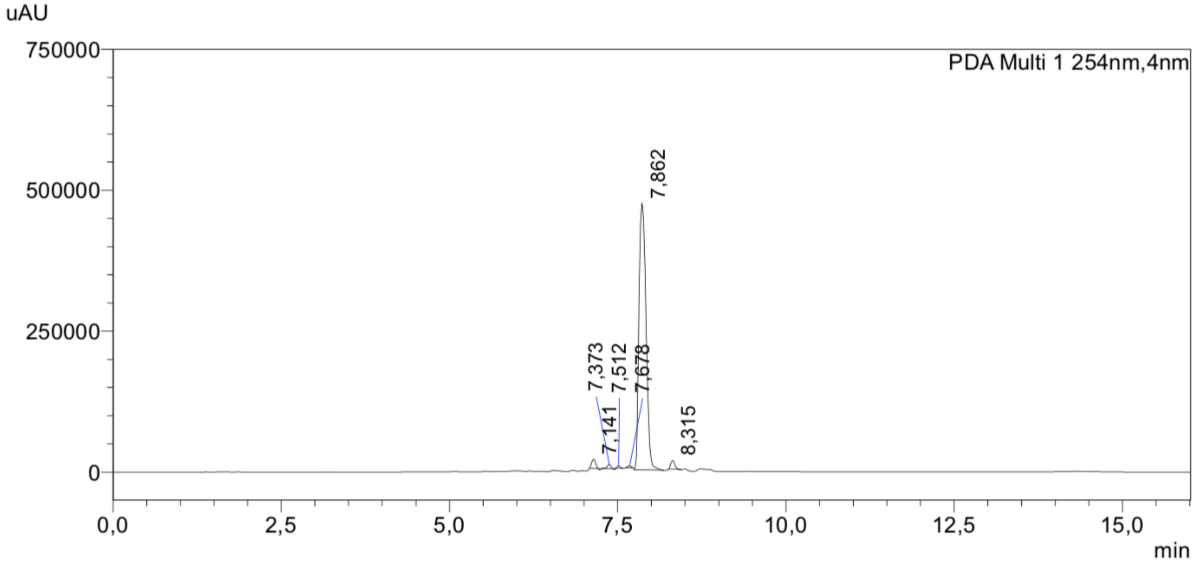

<Peak Table>

PDA Ch1 254nm

Peak#	Ret. Time	Area	Height	Conc.	Unit	Area%
1	7,141	62403	15858	0,000		1,723
2	7,373	28360	7433	0,000		0,783
3	7,512	12173	4046	0,000		0,336
4	7,678	12942	3127	0,000		0,357
5	7,862	3447684	472342	0,000		95,180
6	8,315	58698	15009	0,000		1,620
Total		3622261	517814			100,000

Figure S28: HPLC chromatogram of compound **39**.

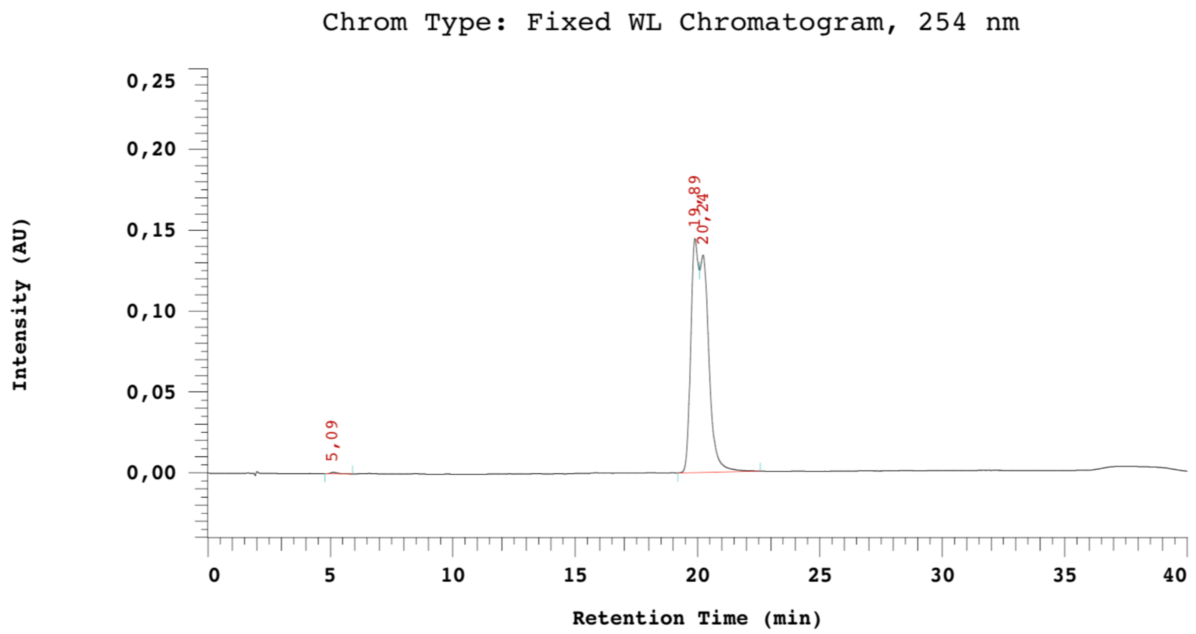

Calculation Method: AREA%

No.	RT	Area	Conc 1	BC
1	5,09	10731	0,317	MC
2	19,89	1544131	45,624	MC
3	20,24	1829574	54,058	MC
		3384436	100,000	

Figure S29: HPLC chromatogram of compound **40**.

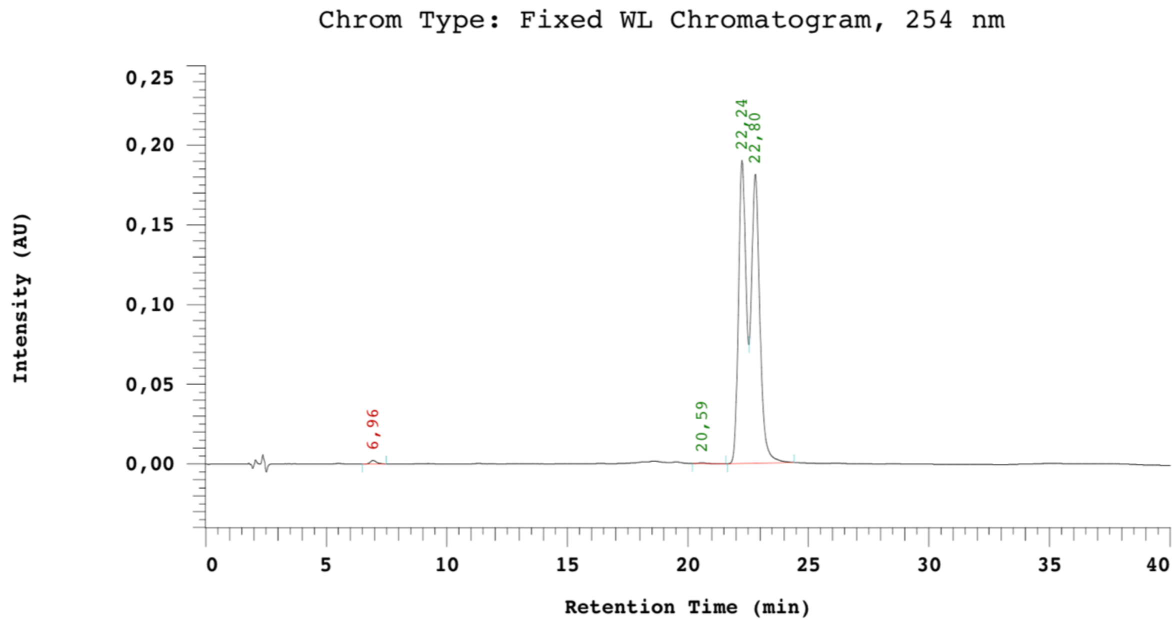

Calculation Method: AREA%

No.	RT	Area	Conc 1	BC
1	6,96	20966	0,482	MC
2	20,59	5209	0,120	BB
3	22,24	2056492	47,231	BV
4	22,80	2271487	52,168	VB
		4354154	100,000	

Figure S30: HPLC chromatogram of compound **41**.

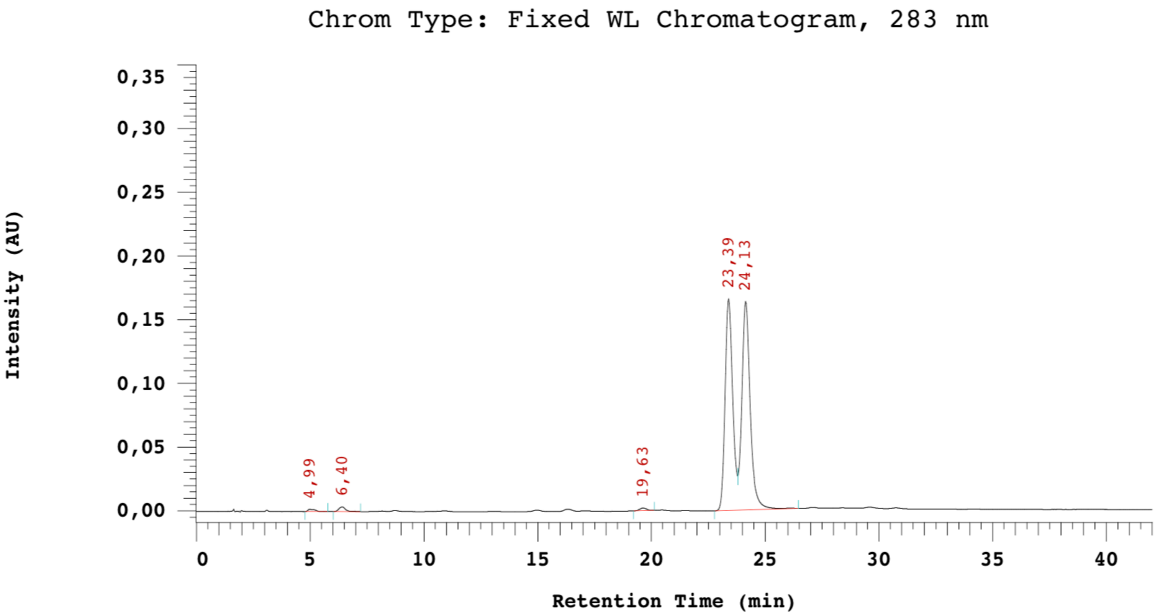

No.	RT	Area	Conc 1	BC
1	4,99	19170	0,469	MC
2	6,40	39251	0,961	MC
3	19,63	21748	0,532	MC
4	23,39	1894752	46,385	MC
5	24,13	2109939	51,653	MC
		4084860	100,000	

Figure S31: HPLC chromatogram of compound **42**.

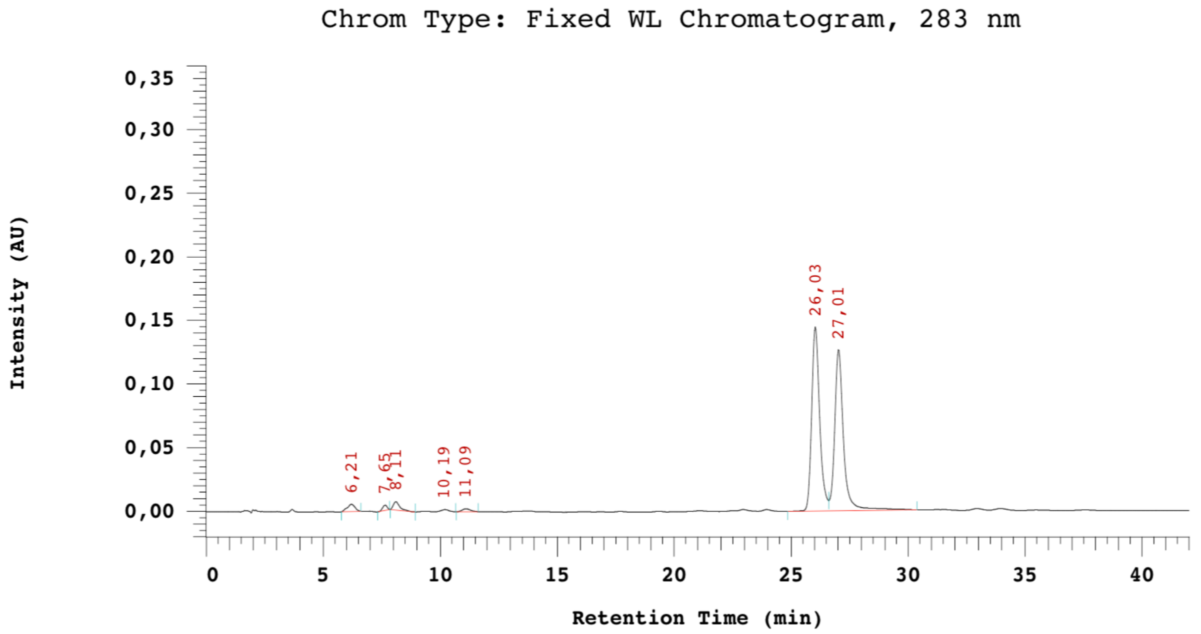

No.	RT	Area	Conc 1	BC
1	6,21	66081	1,872	MC
2	7,65	26026	0,737	MC
3	8,11	61148	1,732	MC
4	10,19	0	0,000	
5	11,09	27917	0,791	MC
6	26,03	1696827	48,070	MC
7	27,01	1651895	46,797	MC
		3529894	100,000	

Figure S32: HPLC chromatogram of compound **43**.

4. Coactivator recruitment

Coactivator recruitment was tested using the LanthaScreen® TR-FRET ERα coactivator assay. The binding of the fluorescein-labeled PGC1a coactivator peptide to the ERα LBD protein labeled with terbium was investigated. As a control, the recruitment upon E2 treatment was assessed.

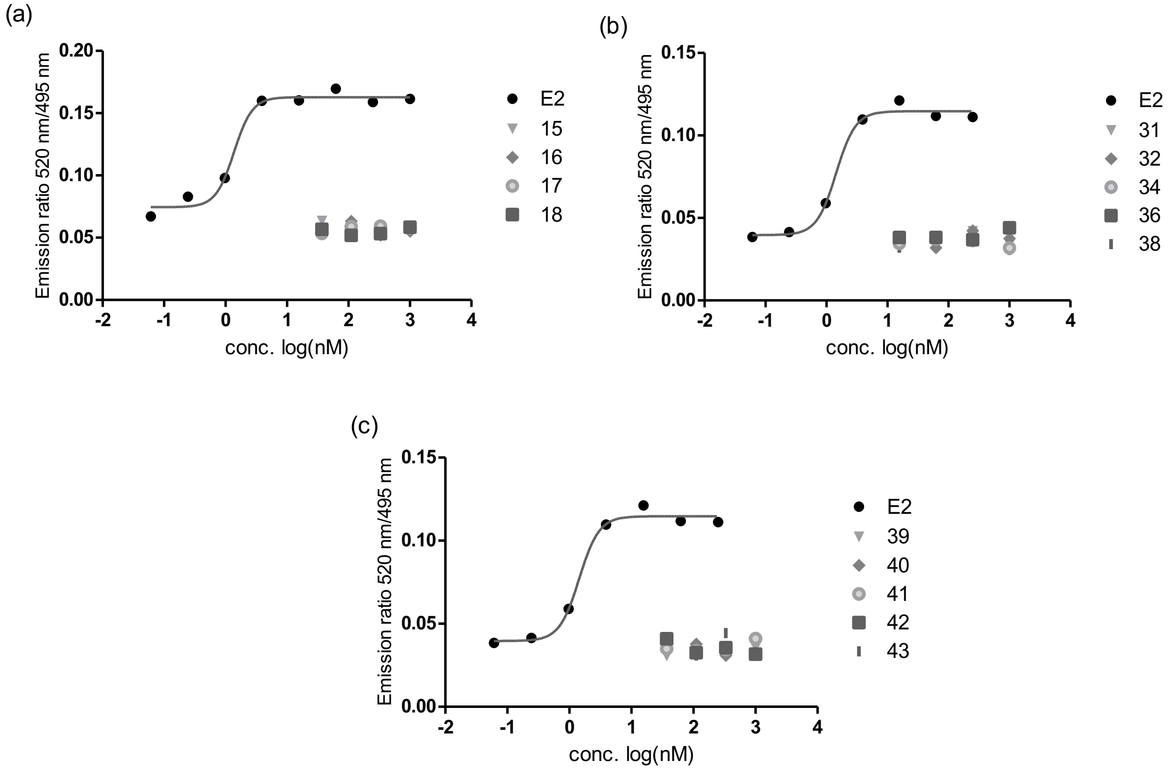

Figure S33: Coactivator recruitment measured by TR-FRET of (a) thioxo-quinazolinones, (b) 5-methoxybenzimidazoles and (c) 5-hydroxybenzimidazoles. The synthesized compounds displayed no coactivator recruitment.

5. Crystal violet assay

To investigate the antiproliferative effects of the heterodimeric ligands, a crystal violet assay was performed using ER-positive (MCF-7), ER-negative (MDA-MB-231), and tamoxifen-resistant breast cancer (MCF-7TamR) cells.

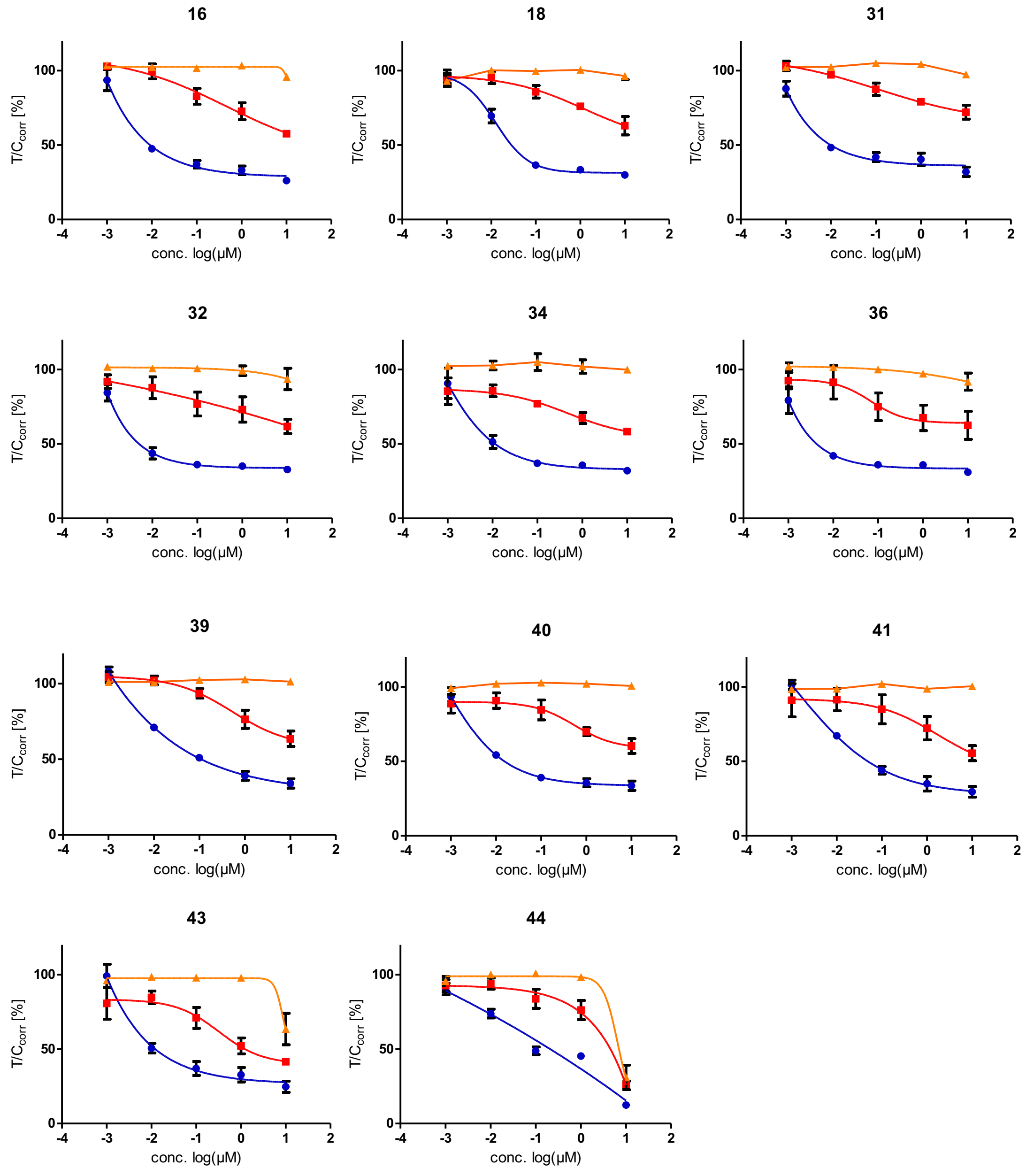

Figure S34: Antiproliferative effects against ER-positive MCF-7 (blue), tamoxifen-resistant MCF-7TamR (red) and ER-negative MDA-MB-231 (orange) breast cancer cells. Values represent the mean ± SD of ≥ 3 independent experiments.

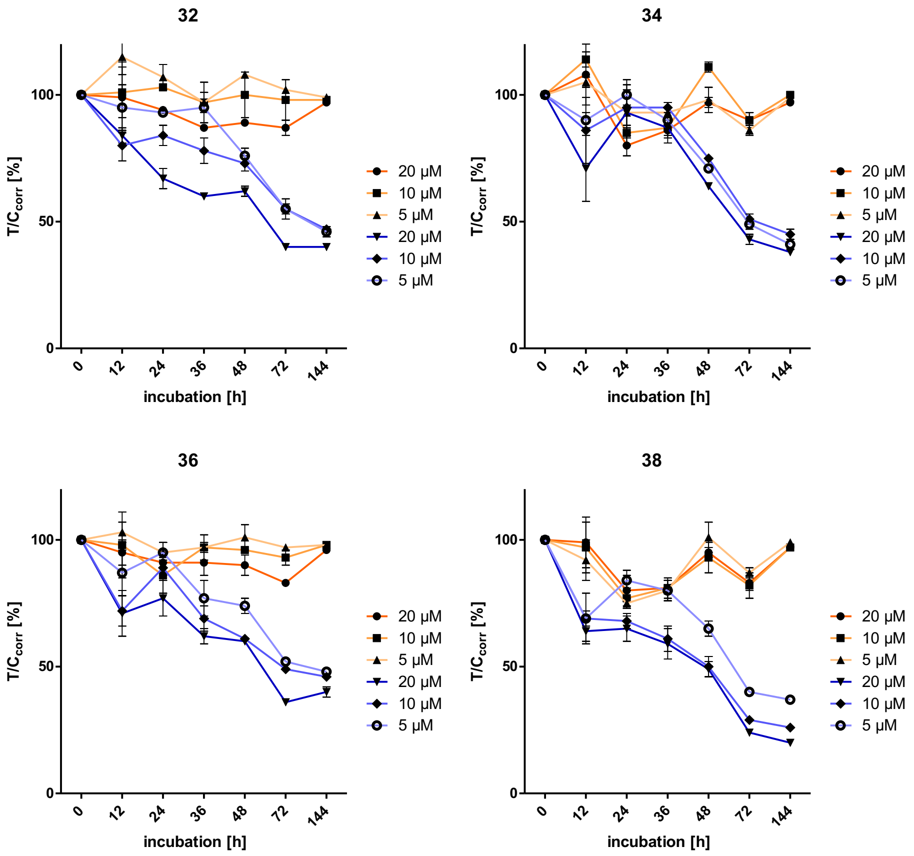

Figure S35: Time-dependent antiproliferative effects against ER-positive MCF-7 (blue) and ER-negative MDA-MB-231 (orange) breast cancer cells. Values represent the mean ± SD of ≥ 3 independent experiments.

6. Transactivation assay

The influence on the signal transduction upon ER binding was investigated with U2OS cells transiently transfected with pSG5-ERα (1 ng) or pSG5-ERβ (1 ng), the reporter plasmid p(ERE)2-luc+ (50 ng) and pRenilla-CMV (0.5 ng) for standardization. The compounds displayed no agonistic activity at 10 µM or 0.1 µM

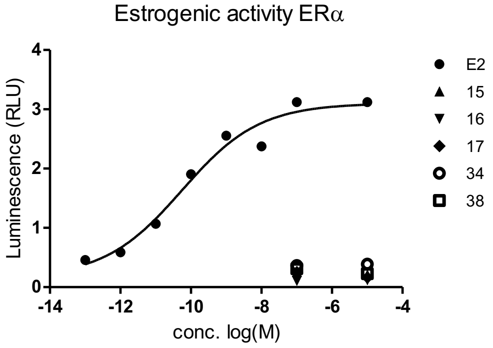

Figure S36: Luciferase reporter gene assay of selected heterodimeric compounds.

7. References

1. Knox, A.; Kalchschmid, C.; Schuster, D.; Gaggia, F.; Manzl, C.; Baecker, D.; Gust, R., Development of bivalent triarylalkene- and cyclofenil-derived dual estrogen receptor antagonists and downregulators. *Eur. J. Med. Chem.* **2020**, *192*, 112-191.
2. Sun, A.; Moore, T. W.; Gunther, J. R.; Kim, M.-S.; Rhoden, E.; Du, Y.; Fu, H.; Snyder, J. P.; Katzenellenbogen, J. A., Discovering small-molecule estrogen receptor α/coactivator binding inhibitors: High-throughput screening, ligand development, and models for enhanced potency. *ChemMedChem* **2011**, *6*, 654-666.
3. Zeng, Q.; Toro, A.; Patterson, J. B.; Wade, W. S.; Zubovics, Z.; Yang, Y.; Wu, Z., Benzoheterocyclecarboxaldehyde derivatives as IRE-1α inhibitors and their preparation and use for the treatment of diseases. WO2011127070A2, **2011**.
4. Hack, S.; Wörlein, B.; Höfner, G.; Pabel, J.; Wanner, K. T., Development of imidazole alkanoic acids as mGAT3 selective GABA uptake inhibitors. *Eur. J. Med. Chem.* **2011**, *46*, 1483-1498.
5. Shan, M., Design, synthesis, and evaluation of bivalent estrogen ligands. PhD-Thesis, Freie Universität Berlin, Berlin, **2011**.
6. Lin, L.-H.; Lee, L.-W.; Sheu, S.-Y.; Lin, P.-Y., Study on the stevioside analogues of steviolbioside, steviol, and isosteviol 19-alkyl amide dimers: Synthesis and cytotoxic and antibacterial activity. *Chem. Pharm. Bull.* **2004**, *52*, 1117-1122.
7. Coste, J.; Le-Nguyen, D.; Castro, B., PyBOP®: A new peptide coupling reagent devoid of toxic by-product, *Tetrahedron Lett.* **1990**, *31*, 205-208.
8. Rivero-Buceta, E.; Carrero, P.; Doyagüez, E. G.; Madrona, A.; Quesada, E.; Camarasa, M. J.; Peréz-Pérez, M. J.; Leyssen, P.; Paeshuyse, J.; Balzarini, J.; Neyts, J.; San-Félix, A., Linear and branched alkyl-esters and amides of gallic acid and other (mono-, di- and tri-) hydroxy benzoyl derivatives as promising anti-HCV inhibitors. *Eur. J. Med. Chem.* **2015**, *92*, 656-671.
9. Vu, C. B.; Bemis, J. E.; Jirousek, M. R.; Milne, J. C.; Smith, J. J., Preparation of fatty acid acylated salicylates for the treatment of inflammatory disorders. US20100184730A1, **2010**.
